# Novel Sulfonamide-Based Carbamates as Selective Inhibitors of BChE

**DOI:** 10.3390/ijms22179447

**Published:** 2021-08-31

**Authors:** Pratibha Magar, Oscar Parravicini, Šárka Štěpánková, Katarina Svrčková, Adriana D. Garro, Izabela Jendrzejewska, Karel Pauk, Jan Hošek, Josef Jampílek, Ricardo D. Enriz, Aleš Imramovský

**Affiliations:** 1 Institute of Organic Chemistry and Technology, Faculty of Chemical Technology, University of Pardubice, Studentska 573, 532 10 Pardubice, Czech Republic; partibha.magar@gmail.com (P.M.); karel.pauk@upce.cz (K.P.); 2 Facultad de Química, Bioquímica y Farmacia, Universidad Nacional de San Luis, Instituto Multidisciplinario de Investigaciones Biológicas (IMIBIO-SL), Chacabuco 915, 5700 San Luis, Argentina; oparravicini@gmail.com (O.P.); adrianagarrosl@gmail.com (A.D.G.); 3 Department of Biological and Biochemical Sciences, Faculty of Chemical Technology, University of Pardubice, Studentska 573, 532 10 Pardubice, Czech Republic; sarka.stepankova@upce.cz (Š.Š.); katarina.svrckova@upce.cz (K.S.); 4 Institute of Chemistry, University of Silesia, Szkolna 9, 40007 Katowice, Poland; izabela.jendrzejewska@us.edu.pl; 5 Department of Pharmacology and Toxicology, Veterinary Research Institute, Hudcova 296/70, 621 00 Brno, Czech Republic; hosek.jan@vri.cz; 6 Department of Analytical Chemistry, Faculty of Natural Sciences, Comenius University, Ilkovicova 6, 842 15 Bratislava, Slovakia; josef.jampilek@gmail.com; 7 Institute of Neuroimmunology, Slovak Academy of Sciences, Dubravska Cesta 9, 845 10 Bratislava, Slovakia

**Keywords:** bioassays, carbamates, cholinesterase inhibitors, molecular modeling, sulfonamides, synthesis

## Abstract

A series of 14 target benzyl [2-(arylsulfamoyl)-1-substituted-ethyl]carbamates was prepared by multi-step synthesis and characterized. All the final compounds were tested for their ability to inhibit acetylcholinesterase (AChE) and butyrylcholinesterase (BChE) in vitro, and the selectivity index (SI) was determined. Except for three compounds, all compounds showed strong preferential inhibition of BChE, and nine compounds were even more active than the clinically used rivastigmine. Benzyl {(2*S*)-1-[(2-methoxybenzyl)sulfamoyl]-4-methylpentan-2-yl}carbamate (**5k**), benzyl {(2*S*)-1-[(4-chlorobenzyl)sulfamoyl]-4-methylpentan-2-yl}carbamate (**5j**), and benzyl [(2*S*)-1-(benzylsulfamoyl)-4-methylpentan-2-yl]carbamate (**5c**) showed the highest BChE inhibition (IC_50_ = 4.33, 6.57, and 8.52 µM, respectively), indicating that derivatives **5c** and **5j** had approximately 5-fold higher inhibitory activity against BChE than rivastigmine, and **5k** was even 9-fold more effective than rivastigmine. In addition, the selectivity index of **5c** and **5j** was approx. 10 and that of **5k** was even 34. The process of carbamylation and reactivation of BChE was studied for the most active derivatives **5k**, **5j**. The detailed information about the mode of binding of these compounds to the active site of both BChE and AChE was obtained in a molecular modeling study. In this study, combined techniques (docking, molecular dynamic simulations, and QTAIM (quantum theory of atoms in molecules) calculations) were employed.

## 1. Introduction

Alzheimer’s disease (AD) is a neurodegenerative brain disorder with characteristic clinical and pathological symptoms [1,2,3]. It represents the leading cause of dementia and affects almost 50 million people worldwide [4]. The exact pathology of AD is still unknown; however, several hypotheses of AD pathogenesis have been suggested, such as deficits in cholinergic neurotransmission, accumulation of β-amyloid outside the neurons and τ-protein inside the neurons, oxidative stress, and inflammation [5,6,7,8,9,10]. Early studies performed on patients suffering from AD found an altered cholinergic activity, which resulted in cognitive and functional symptoms. AD is connected with the decreased concentration of the neurotransmitter acetylcholine (ACh), which is caused by its excessive degradation [5,6]. At the neuronal level, ACh is hydrolyzed by cholinesterases (ChEs) to choline and acetic acid. There are two types of ChEs in vertebrates: acetylcholinesterase (AChE) and butyrylcholinesterase (BChE). AChE plays a crucial role in ACh hydrolysis in cholinergic brain synapses and neuromuscular junctions [11]. BChE is able to hydrolyze ACh as well as other esters and plays probably only a supportive role [12,13,14]. These two enzymes display diverse kinetic characteristics depending on ACh concentrations. AChE is very effective in hydrolysis at low ACh concentrations, while BChE is more efficient in the hydrolysis at high ACh concentrations [15].

In patients with AD, the role of BChE in ACh hydrolysis progressively increases, while AChE activity remains unchanged; its concentration drops down to 90% compared to the healthy brain [14,16]. Clinical studies are in accordance with the lack of effectiveness of AChE inhibitors in the latest stages of AD. Moreover, experiments performed on mouse models demonstrated the role of BChE to maintain the cholinesterase function even in the absence of AChE [17]. This finding is in agreement with earlier reports that showed an inversion of AChE and BChE relative expressions during AD progression [18,19]. In vivo data supporting this hypothesis include the observation that specific BChE inhibitors are able to restore ACh levels in mice [20] and improve the cognitive performance of mice treated with the amyloid-β peptide [21,22] yet without peripheral (parasympathomimetic) adverse effects, [13,20,23] which are known to limit the dosing of AChE inhibitors [13,24,25,26,27,28].

Recently, the treatment of AD has been based on the inhibition of ChEs in order to maintain the proper level of ACh. So far, the U.S. Food and Drug Administration (FDA) has approved four cholinesterase inhibitors for therapy of AD, but tacrine was discontinued due to the aforementioned adverse effects and liver toxicity [29]. Since 2003, no new drugs and even those currently available in clinical practice have been approved. Therefore, the selective inhibition of BChE might well constitute a therapeutic target for clinical use in progressed AD [12,13,29], where AChE inhibitors fail. Due to a number of failures in clinical trials of new candidates for the treatment of AD, ChE inhibitors remain a time-tested therapeutic option for palliative treatment of AD with the aim to improve patients’ cognitive functions [28].

Additionally, the selective inhibition of BChE might be of clinical interest not only for the latest stages of AD. It was found that BChE is involved in the regulation of the serum metabolism in the context of cardiovascular risk factor [30,31], insulin resistance and diabetes mellitus [32,33], and obesity [32,34].

AChE and BChE share 65% amino acid sequence homology [30,31,35]. Their structure is overall similar, although their catalytic active sites (CAS), where the hydrolysis of AChE and BChE is mediated, are quite similar and their active sites, composed of a catalytic triad and a choline binding pocket, are both buried at the bottom of a ~20 Å deep gorge. Two enzymes show differences in the space they provide for a substrate or inhibitor. These differences are notably seen in the amino acids forming the gorge of the binding site and the acyl binding pocket (detailed comparison can be found in ref. [36]). However, selective inhibition of BChE can be achieved by targeting the CAS with carbamate-based inhibitors. These inhibitors generally feature a carrier scaffold guiding a carbamate moiety into the correct position in the enzyme, successively followed by the transfer of the carbamate moiety onto the serine of the CAS under the release of the carrier scaffold. Several carbamate-based selective BChE inhibitors were described [37,38,39,40,41,42].

**Scheme 1:**
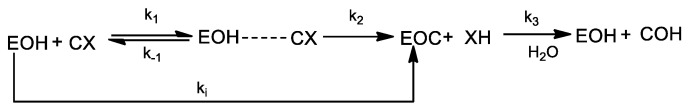
Carbamylation and decarbamylation of AChE [47,48] (EOH is the free enzyme, CX is the carbamate, EOH---CX is the non-covalent complex of enzyme and carbamate, EOC is the carbamoylated enzyme, k_1_ is the rate constant for the formation of Michaelis complex (reversible complex), k_-1_ is the rate constant for dissociation of Michaelis complex, k_2_ is the carbamylation rate constant, k_3_ is dissociation constant, k_i_ is the apparent bimolecular rate constant governing the overall rate of inhibition and neglecting the formation of the reversible complex.

The inhibitors may cause reversible, irreversible or pseudo-reversible inhibition of the enzyme. It is possible to distinguish between the individual types by monitoring the effect of the inhibitor on the active enzyme over time. The reversible inhibitor reduces the activity of the enzyme immediately. However, because it binds to the enzyme only by weak binding, it is released in a short time and the enzyme activity is restored. In the case of irreversible inhibition, the reaction between the enzyme and the inhibitor is not immediate and instead there is a time-dependent decrease in enzymatic activity. The inhibitor binds permanently to the enzyme, and there is no spontaneous restoration of enzyme activity. Pseudo-irreversible inhibitors (e.g., carbamates) bind to the enzyme for a longer period, but unlike irreversible inhibitors, they do not block the enzyme permanently. After some time, they are released from binding, but significantly slower compared to reversible inhibitors. Reactivation of the enzyme inhibited by pseudo-reversible inhibitor can be achieved by dilution or dialysis in buffer [43,44] [A,B]. Wilson and co-workers demonstrated that carbamates serve as slow substrates of AChE, with rapid carbamylation being followed by much slower decarbamylation [45,46] [C,D]. Carbamates form a carbamylated complex with the serine residue of the catalytic triad of AChE. This complex is hydrolysed at slower rate than the acylated form resulting from the interaction between enzyme and substrate (i.e., acetylcholine). The reaction scheme of carbamylation and decarbamylation of AChE is shown in Scheme 1.

A number of recent studies have investigated various modifications of sulfonamides, for example, multitarget agents for the inhibition of AChE enzyme [49], multifunctional agents for Alzheimer’s disease [50], and/or an in vivo active reversible BChE inhibitor [51]. Based on these published data and our previous knowledge (e.g., [41,42,52,53]), we decided to modify chiral sulfonyl chlorides to their benzyl or phenyl derivatives of carbamate-based inhibitors of ChEs. In order to better understand at the molecular level the mode of binding of the compounds reported here to their molecular targets, a molecular modeling study was carried out. In this study, we used different combined techniques such as molecular docking and molecular dynamics simulations using analysis per residue. In addition, in order to evaluate in more details the molecular interactions of different molecular complexes we used quantum theory of atoms in molecules (QTAIM) calculations [54].

## 2. Results and Discussion

### 2.1. Chemistry

**Scheme 2:**
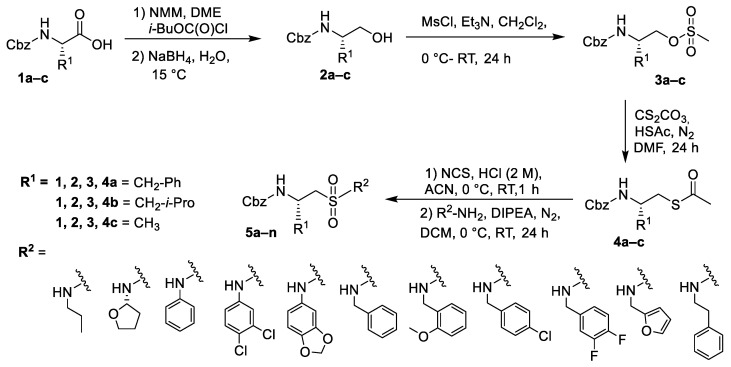
Synthesis of Cbz-N protected chiral sulfonylamides **5a**–**n**.

*N*-Benzyloxycarbonyl (Cbz)-protected chiral benzyl and phenyl sulfonamides were synthesized as outlined in Scheme 2. The Cbz-protected amino acid starting materials were transformed into appropriate alcohols (Cbz-l-alaninol, Cbz-l-phenylalaninol, Cbz-l-leucinol) according to the known procedure [55]. The mesylation of the obtained alcohols with methanesulfonyl chloride in the presence of triethylamine gave methansulfonates **3a**–**c**, which were further transformed using thioacetic acid into thioacetates **4a**–**c** in the presence of cesium carbonate in DMF. *N*-Protected thioacetates reacted with NCS under acidic conditions and provided sulfonyl chlorides **5a–n**. This approach was successfully applied for similar compounds, as evidenced by the literature [56]. Chlorides **5a–n** were used for the last step of the synthesis of targeted sulfonylamides in the presence of a base and a chosen aniline or benzylamine in DCM. The isolated yields of targeted chiral sulfonylamides were approx. 65%.

### 2.2. In Vitro Evaluation of AChE- and BChE-Inhibiting Activity

The ability of newly synthesized derivatives to inhibit AChE from electric eel (AChE) and BChE from equine serum (BChE) was screened in vitro using modified Ellman´s method [57] and compared with that of the standard rivastigmine (RIV) [58]. The inhibitory activity was expressed as IC_50_, representing the concentration of the inhibitor causing 50% inhibition of the enzyme. Based on IC_50_ values for both cholinesterases, selectivity indexes (SI) as the ratio of IC_50_ for AChE/IC_50_ for BChE to quantify the preference for BChE were calculated. The value over 1 indicates more potent inhibition of BChE, whereas SI lower than 1 indicates preferential AChE inhibition. The obtained IC_50_ values and selectivity indexes are summarized in Table 1.

In the series of 14 derivatives, 11 were shown to be more effective in the inhibition of BChE than of AChE (see SI indexes in Table 1). Focusing on inhibition of BChE, IC_50_ values were found in a range from 4.33 μM (**5k**) to 112.21 μM (**5i**). Table 1 shows that nine derivatives (compounds **5c–f**, **5g**, **5h**, **5j**, **5k**, **5m**, **5n**) are more effective in inhibiting BChE than the standard RIV. Derivatives **5c** and **5j** show approximately 5-fold higher inhibitory activity against BChE than RIV and **5k** is even 9-fold more effective than RIV (IC_50_ = 4.33 vs. 38.40 μM) under the given conditions. Derivatives **5c**, **5f**, **5j**, **5k,** and **5n** show significant selectivity for BChE. Specifically, derivative **5k** has a very high SI value (33.71).

In contrast, when comparing the IC_50_ values of the studied derivatives and the standard RIV, it is obvious that only three derivatives (compounds **5d**, **5h**, **5i**) are approximately as effective as RIV in inhibiting AChE. The IC_50_ values were found in a range from 55.64 μM (**5d**) to 150.29 μM (**5k**). Only three derivatives (compounds **5a**, **5b**, **5i**) show a slight selectivity to AChE, however their inhibitory activity is rather weak.

The differences in the inhibitory activity of several inhibitors against AChE and BChE can be explained on the basis of slightly different molecular structure of both enzymes. The different structure of the binding site and the amino acid sequence within the binding site [36] are certainly of great importance. These facts are discussed in detail in the following section.

### 2.3. Evaluation of Carbamylation and Reactivation of BChE

The inhibition process by carbamates is determined by two distinct species: the transient Michaelis complex and the carbamylated enzyme. These two species determine the rate of carbamylation and decarbamylation, respectively, and therefore, both contribute to the efficacy of the carbamate as drug [59]

Two derivatives (compounds **5k**, **5j**) were used for evaluation of carbamylation and decarbamylation of BChE from equine serum. The procedure is described in Section 3.4.

The evaluation of BChE carbamylation was performed on the basis of the measured dependences % residual activity vs. time in the presence of appropriate concentration of inhibitor. Figures showing these dependencies (Figures S2 and S3) are presented in the Supplementary Materials. The obtained dependences % residual activity vs. time in the presence of **5k** (**5j**) show very similar course. Initially the enzymatic activity decreased, but after some time the decrease stopped and a longer incubation time did not lead to any further decrease of the activity. On the contrary, after about 30 h, the activity began to rise slowly again. These dependences were evaluated by non-linear regression and the pseudo-first-order rate constants k_obs_ for progressive inhibition at several inhibitor concentrations were obtained. Subsequently, the dependence of k_obs_ vs. concentration of inhibitor was plotted and the apparent bimolecular rate constant (k_i_) was determined: 92.67 ± 5.62 M^−1^min^−1^ for **5k** and 65.04 ± 3.21 M^−1^min^−1^ for **5j**. The dependences k_obs_ vs. concentration of inhibitor are presented in the Supplementary Materials as Figures S4 and S5. Bar-On et al. [48] studied the carbamylation of several different enzymes by several inhibitors. The bimolecular rate constant for the carbamylation of human BChE by rivastigmine was 9 × 10^4^ M^−1^min^−1^, so 1000-fold higher than the results we have achieved.

Further, the reactivation of BChE from equine serum inhibited by derivatives **5k** (**5j**) was studied. The procedure is described in Section 3.4. The dependence % restored activity vs. time is shown in Figure S6 presented in the Supplementary Materials. From the obtained results it is evident that the reactivation of BChE from equine serum inhibited by **5k** is about 30% after 24 h, while upon inhibition by **5j** 37% reactivation was achieved after 24 h. Bar-On et al. [48] studied spontaneous decarbamylation of several enzymes inhibited by rivastigmine or *N,N*-ethylmethylcarbaryl chloride (EMCC). According them the reactivation of human BChE inhibited by rivastigmine is very slow (<10% after 24 h). In comparison, upon dilution complete and rapid reactivation of human BChE inhibited by EMCC was observed.

### 2.4. Molecular Modeling Studies

In order to explain the experimental results, we performed a molecular modeling study. Calculations were carried out in three stages and led us to the identification of intermolecular interactions involved in the formation of different complexes. In this regard, successive docking calculations, molecular dynamics (MD) simulations, and a per-residue free energy decomposion analysis of each enzyme-ligand complex of both AChE and BChE were carried out. In order to evaluate in more details the molecular interactions of different molecular complexes, in the last stage of our study we carried out QTAIM calculations. This methodology was successfully applied in previous works [60,61,62,63,64,65]. Indeed, we have formerly reported and described the molecular interactions established in the active site of AChE and BChE when complexed with the well-known cholinesterase inhibitors RIV [66,67] and galantamine [68,69,70,71] and other ligands with great structural variability, including alkaloids [69,70], carbamates [67], 4-[(alkoxycarbonyl)amino]benzoates [66], and *N*-benzyl-2-phenylethanamine derivatives [72].

From Table 1, it is noticeable that most sulfonamide derivatives studied here displayed a strong BChE inhibitory activity. In fact, some of them showed lower IC_50_ values than those obtained for RIV, the reference compound. However, some differences should be noted considering the molecular structure of these compounds. As can be seen, inhibitory activity increases as the substituent at R^1^ position varies from 5a to 5c. It appears that the presence of an isobutyl substituent at R^1^ (**5c**, IC_50_ = 8.52 µM) has a significant importance for biological effects. Notice that IC_50_ increases in compounds **5a** and **5b** (IC_50_ = 100.25 and 86.12 µM, respectively), bearing methyl and benzyl substituents, respectively. Nevertheless, these considerations might not be sufficient to explain the inhibitory activity, since similar IC_50_ values were obtained for compounds **5g** and **5n** (with a benzyl and an isobutyl group at R^1^, respectively). Accordingly, substituents at R^2^ are worth discussing. The most active compounds, **5c**, **5j** and **5k** (IC_50_ < 10 µM), bear a benzyl or a monosubstituted benzyl ring at R^2^. On the other hand, the presence of a phenyl (**5d**, **5g** and **5n**), a ethylphenyl (**5f**), a benzo[1,3]dioxol (**5h**), or a furanyl (**5m**) substituent at R^2^, leads to a slightly lower inhibitory activity than that of the previous compounds. Note that these derivatives displayed lower IC_50_ values than RIV as well. Lastly, either non-aromatic substituents (**5e** and **5i**) or disubstituted benzyl rings (**5l**) led to a loss of the inhibitory effects (IC_50_ > 58 µM).

**Figure 1:**
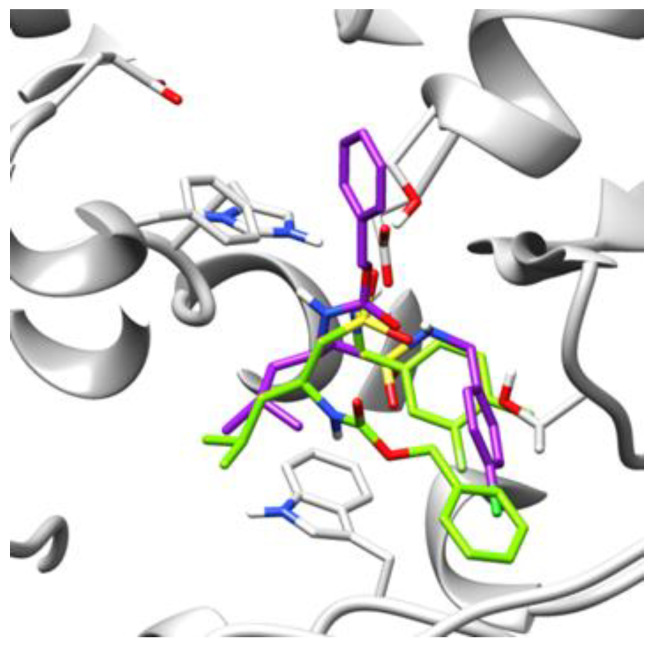
View of spatial configuration adopted by **5j** (violet) and **5l** (green) within the active site of BChE. Notice the extended conformation of **5j** (IC_50_ = 6.57 µM) and the V-shaped conformation of **5l** (IC_50_ = 55.64 µM).

From docking studies, we could infer that, while active compounds adopt an extended molecular conformation within the BChE active site, derivatives with high IC_50_ values are folded adopting a V-shaped conformation (Figure 1). This conformation would favor the formation of intramolecular bonds, while reducing the possibility of establishing intermolecular interactions with residues from the active site of the enzyme. Consequently, either stronger or larger interactions could be expected for active compounds.

From MD simulations, the root mean square deviations (RMSDs) of backbone atoms of protein and ligand atoms in BChE-**5k** and BChE-RIV complexes are illustrated in Figure S1A,B (both in the Supplementary Materials), respectively. For all systems, the low deviations of RMSD revealed the stabilities of the ligand-BChE complexes throughout the entire trajectory. Moreover, the RMSDs were distributed around 1.3–1.8 Å for backbone atoms of BChE and 1.7–2.7 Å for ligand atoms. These stable trajectories were then used to calculate the root mean square fluctuation (RMSF) for BChE Cα atoms (Figure S1C). The RMSF patterns were similar for both systems. Notice that residues with large fluctuations are located in loop regions, whereas residues from the CAS were found to be stable. Both RMSD and RMSF results suggested the stabilities of the studied systems.

**Figure 2:**
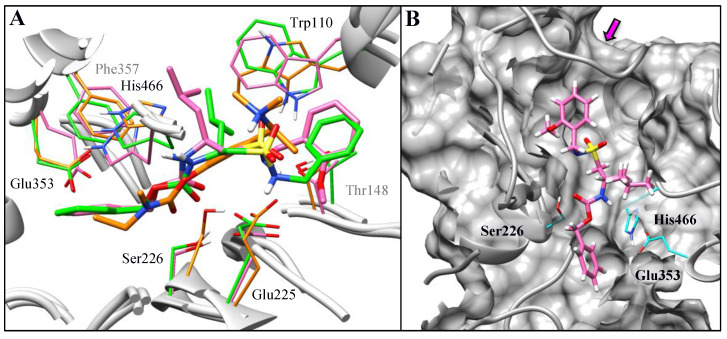
(**A**) Spatial view of BChE-**5c** (green), BChE-**5k** (pink) and BChE-RIV (orange) overimposed for comparison; (**B**) Active site of *Equus caballus* BChE when complexed with compound **5k** (pink).

Our calculations suggest that sulfonamide derivatives studied here and the commercial drug RIV interact at the same region of the BChE active site (Figure 2A), which can be described as a narrow, 20 Å long pocket. On the base of this pocket, the triad Ser226, Glu353, and His466, termed catalytic anionic site (CAS), is located. towards the reactive Ser226. Similar results were obtained for all active ligands. Catalytic sites of cholinesterases have been studied in detail and it is well known that this serine residue plays a key role in the inhibition of cholinesterases mediated by carbamate-type inhibitors [48,73,74,75].

From Figure 2B, it is possible to observe that active compound **5k** is deeply buried into the active site gorge. It is important to note that the carbamate moiety is favorably oriented. 

**Figure 3:**
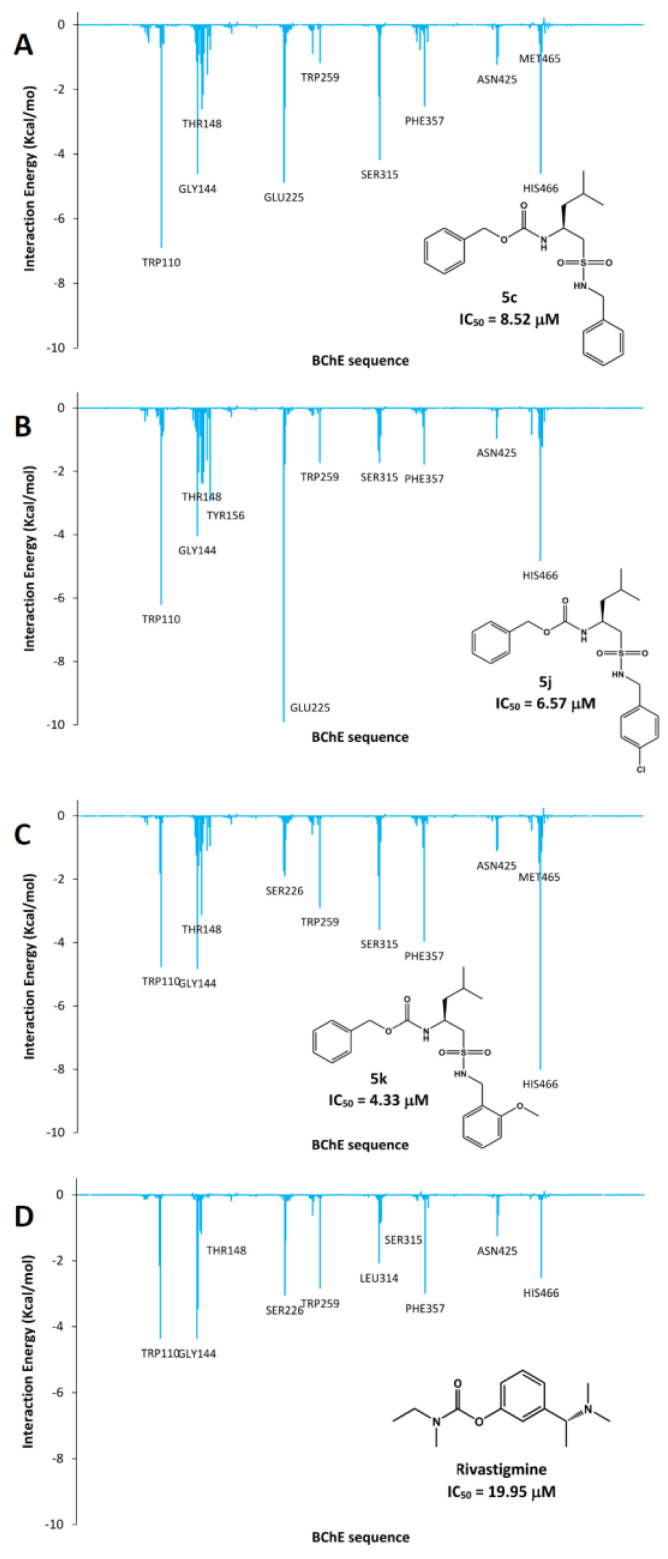
Histogram of interaction energies partitioned with respect to BChE amino acid sequence when complexed with compounds **5c** (**A**), **5j** (**B**), **5k** (**C**), and RIV (**D**). The X-axis denotes the residue number of BChE, and the Y-axis denotes the interaction energy between the compound and a specific residue. Negative and positive values are favorable or unfavorable for binding, respectively.

Figure 3 shows the histogram of the interaction energies obtained for BchE-**5c**, BChE-**5j,** and BChE-**5k** complexes. The histogram obtained for BChE-RIV is also included for comparison. As it can be seen, the main interactions stabilizing these complexes are with the following amino acid residues: Trp110, Gly144, Thr148, Glu225, Ser226, Trp259, Ser315, Phe357, Asn425, and His466. These interactions have been previously reported by our group for other carbamate derivatives showing butyrylcholinesterase inhibitory activity [67,72]. Notice that interactions that contribute to the BChE complexes formation of the most active ligands are identical to those established by RIV at the active site of BChE. In fact, **5c**, **5j,** and **5k** displayed interaction energy values with Ser226 ranging from −2.5 to −2 Kcal/mol. These values are comparable to that obtained for BChE-RIV complex.

**Figure 4:**
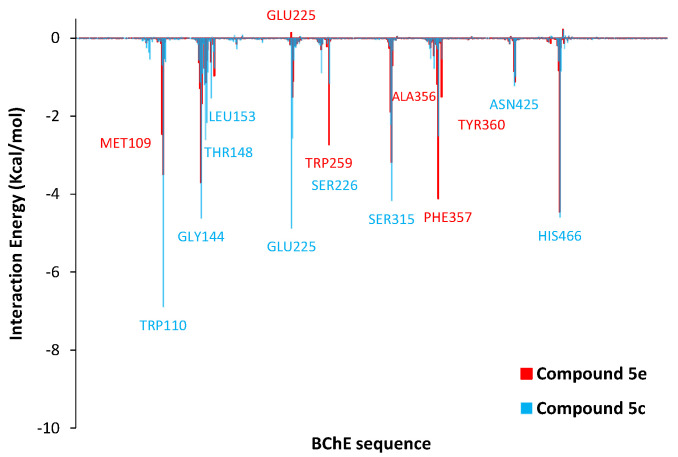
Histogram of interaction energies partitioned with respect to BChE amino acid sequence when complexed with compounds **5c** (blue) and **5e** (red) overlaid for comparison. The X-axis denotes the residue number of BChE, and the Y-axis denotes the interaction energy between the compound and a specific residue. Negative and positive values are favorable or unfavorable for binding, respectively.

Conversely, less active compounds established weaker interactions with BChE than those discussed above. This behavior can be evaluated in Figure 4, which shows the histograms of compounds **5c** (IC_50_ = 8.52 µM) and **5e** (IC_50_ = 58.94 µM) overlaid for comparison. Remarkable differences can be observed for interaction with Trp110, Thr148, Glu225, Ser226, and Ser315, some of the most important interactions displayed by active compounds. In addition, interactions with Trp259 and Phe357, located near the surface of the pocket, increased in the case of compound **5e**. An interesting detail that appears from the analysis of the partitioned free energy is that inactive compound **5e** interacts with residues Met109, Ala356, and Tyr360 that are not present in BChE-**5c**. Analogously, interactions with Thr148, Gly149, Thr150, and Leu153 from BChE-**5c** may either have higher values or be absent in BChE-**5e**. The lack of the last interactions might be related to the V-shaped conformation adopted by inactive compounds, which causes interactions of the sulfonamide moiety of these derivatives (R^2^) with different regions of the enzyme active site. Similar results were obtained for the rest of the ligands with IC_50_ values higher than those of rivastigmine.

Considering AChE inhibition, all ligands displayed higher IC_50_ values than RIV and only compound **5d** exhibited a comparable inhibition. These results could be expected, because large compounds, bearing bulky substituents and exhibiting inhibitory activity against BChE, usually do not display biological effects on AChE. This could be explained, since, although both enzymes share almost 60% of their amino acid sequence, their active sites differ in some key residues [48]. Particularly, the bulkier aromatic residues Tyr121 and Phe330 in AChE are playing a similar role as Gln147 and Ala356 of the BChE active site. These two aromatic amino acids constitute the bottleneck region of the AChE active site, resulting in a very narrow area in the gorge that limits the size of ligands that can access the bottom of the active site. As a result, since the BChE active site presents a lower number of aromatic residues in its binding pocket, this enzyme possesses a larger accessible area than AChE. This explains, at least in part, why despite the fact that cholinesterases show similar responses to classical inhibitors, in cases of bulkier compounds, selectivity for BChE over AChE generally appears.

**Figure 5:**
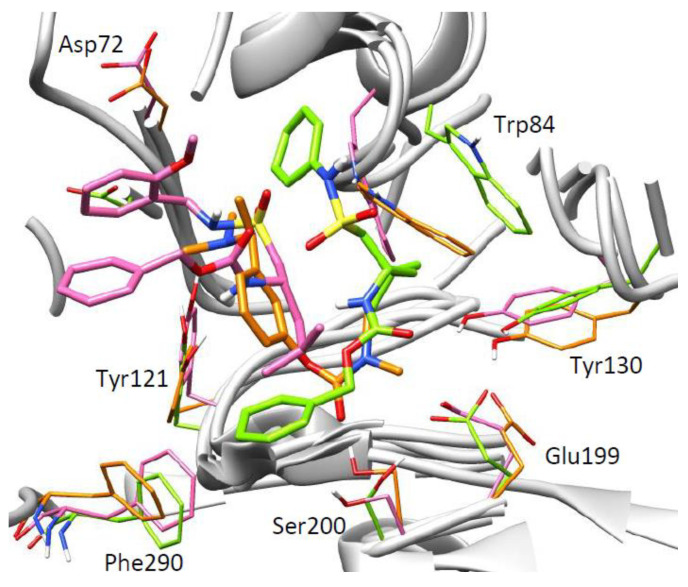
Spatial view of AChE-**5d** (green), AChE-**5k** (pink), and AChE-RIV (orange) complexes overimposed for comparison.

In Figure 5, the spatial view of compounds **5d** and **5k** within the active site of AChE is shown. These derivatives represent the most and the least active compounds of the whole series. For comparison, we have also included a spatial view of RIV in this figure; the data for the coordinates of this molecule were taken from our previously reported article [66]. It is evident that compound **5d** interacts with active site residues in a completely different manner than **5k**. Similarly to the results obtained for BChE, active compound **5d** adopts an extended conformation, being able to reach the bottom of the active site thus efficiently interacting with the catalytic triad. As can be seen, even though the spatial conformations of **5d** and RIV are not exactly the same, the carbamate group is located in the same region. Thus, the carbamate group of **5d** is properly positioned to generate an interaction with the catalytic serine residue, Ser200. On the contrary, **5k** adopts a V-shape conformation as discussed for BChE inactive ligands. Furthermore, **5k** is shifted towards the surface of the pocket and, therefore, it interacts with residues from the peripheral anionic site and the bottleneck region.

**Figure 6:**
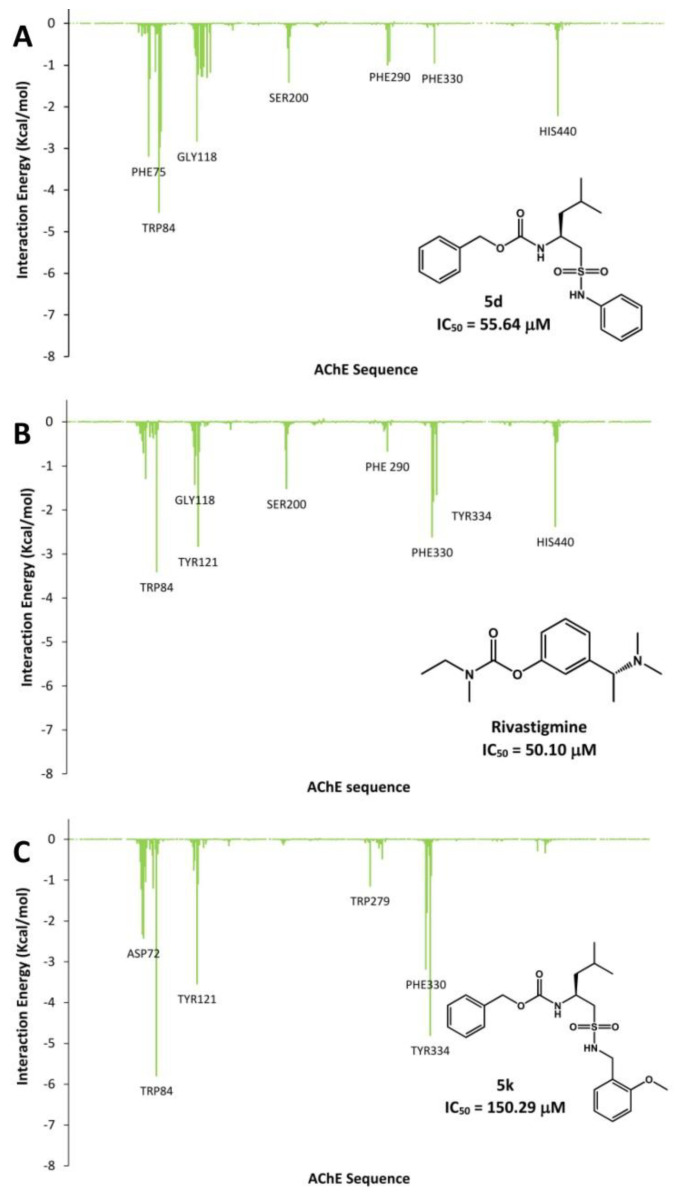
Histogram of interaction energies partitioned with respect to AChE amino acid sequence when complexed with compound **5d** (**A**), RIV (**B**), and compound **5k** (**C**). The X-axis denotes the residue number of BChE, and the Y-axis denotes the interaction energy between the compound and a specific residue. Negative and positive values are favorable or unfavorable for binding, respectively.

This behavior might be better evaluated in Figure 6. The histogram of the interaction energy of compound **5d** shows an interaction pattern similar to that of the reference compound. In particular, interactions with Trp84, Gly118, Ser200, Phe290, Phe330, and His440 are the main interactions observed in AChE-**5d** and AChE-RIV complexes (compare Figure 6A,B). In contrast, the histogram of **5k** demonstrates that no interaction with Ser200 takes place in the active site (Figure 6C). Furthermore, the main stabilizing interactions in AChE-**5k** complex are those with Asp72, Trp279, Tyr334 (peripheral anionic site), Tyr121, and Phe330 (bottleneck region). Identical results were obtained for the rest of the compounds.

**Figure 7:**
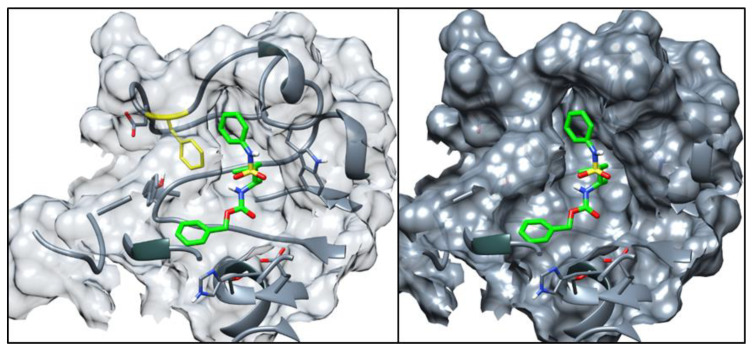
Two different representations of the active site of *Torpedo californica* AChE when complexed with compound **5d** (green). The gorge of the enzyme is depicted by its molecular surface in (semi-transparent) gray. The Phe75 residue is depicted in yellow. The amino acid residues from the catalytic triad are also shown.

One of the major differences between the histograms in Figure 6A,B is the presence of a strong interaction of residue Phe75 with the sulfonamide derivative, which is notably weaker for AChE-RIV (−3.19 Kcal/mol vs. −0.13 Kcal/mol). This interaction might be of significant importance for the sulfonamide compounds studied here and could be associated to a π-stacking interaction between the aromatic ring of Phe75 and the phenyl substituent at R^2^ in **5d** (Figure 7). This interaction might be crucial for the ligands to adopt a biologically active conformation within the active site. This could explain the dissimilar IC_50_ value obtained for compound **5e**. Notice that this compound, despite being structurally much smaller, is about twice less active than **5d**. Furthermore, the *N*-phenylsulfonamide moiety is located in a spatial region with very limited available space due to steric factors (Figure 7). Consequently, any substituent that increases the size of this portion of the ligand molecule will cause the inability of the aromatic ring to interact on this hindered zone, especially with Phe75. In this way, a decrease in the inhibitory activity would occur. This might be the case of compound **5k**, bearing an o-methoxybenzyl ring at R^2^.

#### QTAIM Calculations

Previously we have used QTAIM calculations as a complementary technique for dynamic approaches. We have proved that QTAIM calculations are a useful tool in the study of enzyme-ligand complexes with different structural complexity, since they provide accurate information of molecular interactions that take part in complexes formation [62]. In addition, we have recently applied this technique to the study of different complexes of both AChE and BChE [66,67]. Based on those results, we carried out a QTAIM study for compounds **5c** and **5k**, both with high inhibitory effect against BChE.

**Figure 8:**
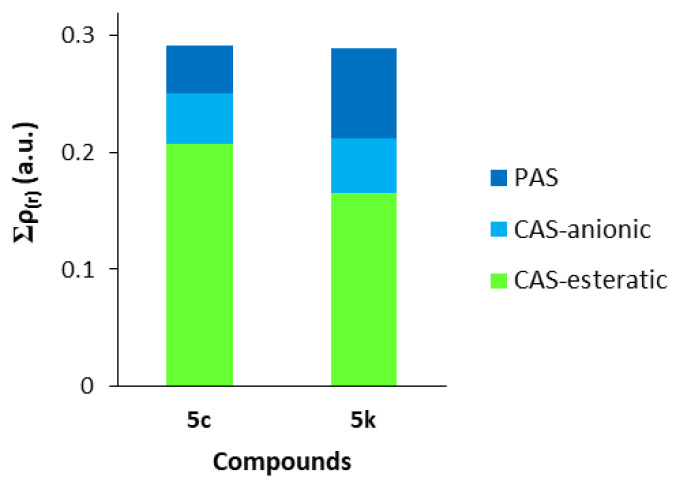
Sum of charge density values at intermolecular bond critical points between BChE and the selected compound. The total height of stacked bars indicates the overall anchoring strength of compounds within the active site. The CAS is devided into the catalytic esteratic subsite (CAS-esteratic) and the catalytic anionic subsite (CAS-anionic).

The active site of cholinesterases has two binding sites: the catalytic anionic site (CAS, at the bottom of the gorge) and the peripheral anionic site (PAS, near the surface). Furthermore, CAS can be sub-divided into two regions termed esteratic and anionic subsites. As reaction occurs, the substrate interacts with residues that form the esteratic subsite (CAS-esteratic), where the catalytic triad is located. At the same time, interactions with the anionic subsite (CAS-anionic), which borders the gorge leading to the active site, are also formed. In a recent article [67], we proposed that a combination of both interactions with the catalytic site and strong interactions with the peripheral anionic site might be beneficial for the inhibitory effect of carbamate-type ligands. In this regard, the stacked bars in Figure 8 show a dissected view of the anchoring of sulfonamide derivatives to the different sites within the BChE pocket. In both cases, it is possible to observe that the main stabilizing interactions are with amino acids from the CAS-esteratic. In turn, interactions with the CAS-anionic residues resulted in similar Ʃρ values. On the contrary, **5k** displayed stronger interactions with residues from the PAS than **5c**. This behaviour might be related to the higher inhibitory effect displayed by **5k**. Although a slightly higher total Ʃρ value was obtained for **5c**, the molecular structure of **5k** and its conformation within the complex would lead to more effective interactions, since a considerable anchoring to both CAS and PAS is possible.

**Figure 9:**
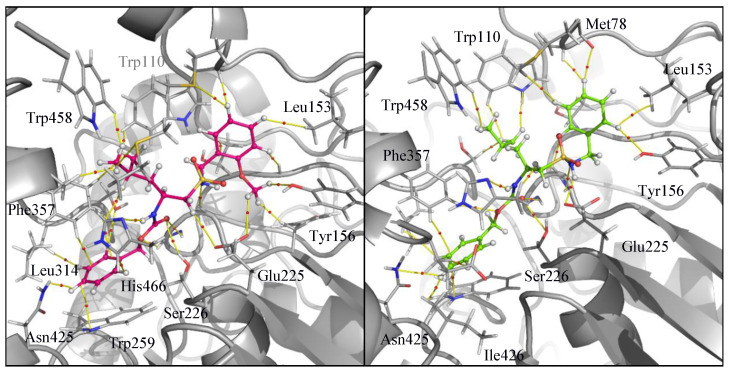
Charge density molecular graph of compounds **5c** (A, pink) and **5k** (B, green) at the gorge of BChE. Residues from active site are shown in grey. Topological elements of charge density associated with intermolecular interactions are depicted with yellow lines (bond paths) and small red spheres (bond critical points).

Charge density molecular graphs of **5c** and **5k** complexed with BChE are shown in Figure 9. As can be seen, both ligands are capable of reaching the bottom of the gorge, thus interacting within the active site of BChE in a similar manner. The benzyl carbamate moiety is located at the bottom of the gorge. Therefore, this aromatic ring establishes several hydrophobic interactions with neighbouring residues, such as Gly145, Leu314, Ser315, Val316, Phe357, and Asn425. Additionally, a strong π-stacking interaction with Trp259 is formed. Simultaneously, the second aromatic ring from the sulfonamide moiety (R^2^) is surrounded by Trp110, Tyr142, Gly144, Thr148, Leu153, Tyr156, and Glu225. Consequently, several hydrophobic O···H and S···H interactions with non-polar hydrogen atoms can be observed. Considering the isobutyl chain at R^1^, similar hydrophobic interactions are established with Ala356, Phe357, Trp458, and Met465.

All these interactions lead to anchoring of compounds **5c** and **5k** within the active site of the enzyme. Furthermore, it should be pointed out that this conformation results in the favourable orientation of the carbamate group, which faces the catalytic Ser226. Notice that carbamoyl oxygen atom is H-bonded to the serine residue. Moreover, a second H-bond is formed between the NH of the carbamate group and the N atom of the imidazole ring from His466. Our calculations agree with cholinesterase inhibition data, which enable us to explain the biological activity of compounds considered in this work.

### 2.5. Cytotoxicity Evaluation and ADME Predictions

To evaluate the general safety of the tested compounds, the cytotoxic effect on human monocytic leukemia THP-1 cell line was measured. Although this cell line is relatively sensitive to different types of xenobiotics, used AChE and BChE inhibitors did not significantly influence the relative cell viability at the concentration of 20 µM. At this concentration, the relative cell viability was still above 85%. It indicates that the most potent BChE inhibitors with IC_50_ < 10 µM could be safe for further cell-based in vitro and in vivo analysis.

The ADME (the abbreviation for absorption, distribution, metabolism, and excretion) properties of compounds characterizing pharmacokinetics are as important as the biological effect of a drug [76,77,78,79]. Physicochemical properties affecting permeability, bioaccumulation in cells, binding to the target site, and biotransformation belong to the area of quantitative structure–property relationships (QSPR) and are influenced by chemical composition [78,79,80,81,82]. In this context, it is necessary to mention Lipinsky’s rule of five (Ro5), which has become one of the most important rules used in the design of bioactive agents, and the parameters listed in it are among the recognized criteria affecting ADME and bioactivity [78,79,82,83,84].

Ro5 contains the limits of specific molecular descriptors (see Table 2) determined on the basis of experimentally and statistically obtained results [83,84]. A biologically active compound that meets these criteria has a higher chance of becoming a drug (it meets the concept of druglikeness). Table 2 lists the Ro5 parameters of selected most pronounced BChE inhibitors **5c**, **5f**, **5j**, **5k**, and **5n** as well as some other most used criteria. All the parameters were predicted using ACD/Percepta (Advanced Chemistry Development. Inc., Toronto, ON, Canada, 2012) and are compared with those of rivastigmine and galantamine.

Based on the predicted in silico data presented in Table 2, it can be stated that all the investigated compounds meet the Ro5 requirements. Compared to both drugs, the investigated compounds contain a greater number of heretoatoms, which is reflected in the significantly higher polar surface area (TPCA). On the other hand, they have a higher predicted lipophilicity (logP) than both drugs, so they are expected to show sufficient intestinal permeability when administered orally. RIV is a strong base, and drugs of this type are mostly bound to α1-acid glycoprotein in plasma; but some also interact with human serum albumin. Galantamine is a zwitterionic compound, and such molecules bind to most plasma proteins. On the other hand, all the investigated compounds are acidic compounds; in plasma, this type of molecules binds predominantly to albumin, which corresponds to the values of log*K*_a_^HSA^ (the parameter represents the binding constant between the compound and human serum albumin (HSA)) and %PPB. It should be noted that the calculated data suggest that the studied compounds may bind to plasma proteins with a high binding ratio. As is known, serum protein prefers the binding of weakly acidic compounds. These compounds are hydrophobic in nature, so they are expected to bind mainly to fat-soluble proteins but would not interact well with serum albumin. According to the predicted indicators of permeation through the blood-brain barrier (logPS, logBB), it is assumed that the penetration of the tested compounds into the brain will be sufficient for the CNS activity.

## 3. Materials and Methods

### 3.1. General

All reagents and solvents were purchased from commercial sources (TCI Europe, Sigma-Aldrich, Acros Organics, Fluorochem, Merck, Lach-Ner). Commercial grade reagents were used without further purification. Reactions were monitored by thin layer chromatography plates coated with 0.2 mm silica gel 60 F254 (Merck). TLC plates were visualized by UV irradiation (254 nm) or in a 5% solution of phosphomolybdic acid in ethanol. Melting points were determined on a Melting Point B-540 apparatus (Büchi, Switzerland) and are uncorrected. The IR spectra were recorded on a Nicolet 6700 FT-IR spectrometer (Thermo Fisher Scientific, Wal-tham, MA, USA) over the range of 400-4000 cm-1 using the ATR technique. The NMR spectra were measured in CDCl3 solutions at ambient temperature on a Bruker AvanceTM III 400 spectrometer at frequencies of 1H (400 MHz) and 13C (100.26 MHz) or Bruker AscendTM 500 spectrometer at frequencies 1H (500.13 MHz), 13C (125.76 MHz). The chemical shifts, δ, are given in ppm, related to the residual solvent peak CDCl3 - 7.27. The coupling constants (J) are reported in [Hz]. Elemental analyses (C, H, N) were performed on an automatic microanalyser Flash 2000 Organic elemental analyser. High-resolution mass spectrometry was performed by the “dried droplet” method using an LTQ Orbitrap XL MALDI mass spectrometer (Thermo Fisher Scientific) equipped with a nitrogen UV laser (337 nm, 60 Hz). Spectra were measured in positive ion mode and in regular mass extent with a resolution of 100 000 at m/z = 400. 2,5-Dihydrobenzoic acid (DBH) was used as the matrix.

### 3.2. Chemistry

#### 3.2.1. General Experimental Procedure for the Synthesis of N-Cbz Alcohols **2a–c**

To a solution of *N*-Cbz α-amino acid 1 (2 g, 6.68 mmol) in 1,2-dimethoxyethane (10 mL), *N*-methyl morpholine (0.68 g, 6.68 mmol) and isobutylchloroformate (0.913 g, 6.68 mmol) were added at −15 °C. After some time, a white precipitate was generated, which was removed by vacuum filtration and washed with DME (5 × 2 mL). The filtrate and washings were combined in a large beaker in an ice salt bath. A solution of NaBH_4_ (0.379 g, 10.02 mmol) in 5 mL water was added in one portion to the beaker, which produced a strong evolution of gas, and immediately water (250 mL) was added. After some time, a white precipitate occurred and was collected by vacuum filtration and washed with water and *n*-hexane to afford the product **2a** in 96% yield. In other cases (compounds **2b, 3b**), the compound was extracted with ethyl acetate and purified by a classical aqueous work-up to afford 91% and 88% yields, respectively. *Benzyl (S)-(1-hydroxypropan-2-yl)carbamate* (**2a**): White solid; yield 96%; mp 93–95 °C; R_f_ (hex/EtOAc–1/1) = 0.53. IR (ATR): 3347, 3035, 2956, 2879, 1690, 1533, 1464, 1454, 1311, 1251, 1215, 1140, 1012, 741, 698 cm^−1^. ^1^H-NMR (400 MHz, CDCl_3_): δ 7.44–7.34 (7H, m, Ar-*H*), 7.29 (3H, q, *J* = 7.2 Hz, Ar-*H*), 5.32–5.14 (3H, ABq, *J* = 8.8 Hz, O-C*H_2_*-Ph, N*H*-CH-CH_2_-Ph), 4.02 (1H, s, NH-C*H*-CH_2_-Ph), 3.73 (1H, d, *J* = 8.8 Hz, C*H*H-OH), 3.63 (1H, d, *J* = 9.6 Hz, CH*H*-OH), 2.92 (2H, d, *J* = 6.8 Hz, NH-CH-C*H_2_*-Ph), 2.44 (1H, s, CH2-O*H*). ^13^C-NMR (100 MHz, CDCl_3_): δ 156.7, 137.8, 136.5, 129.5, 128.8, 128.7, 128.4, 128.3, 126.8, 67.0, 64.1, 54.3, 37.5. CHN analysis: Calc. for C_17_H_19_NO_3_ (285.34): C, 71.56; H, 6.71; N, 4.91. Found: C, 71.87 ± 0.03; H, 6.82 ± 0.02; N, 4.73 ± 0.02. HRMS: *m*/*z* calc. C_17_H_19_NO_3_: 286.14377 [M + H]^+^; 308.12571 [M + Na]^+^; found: 286.14440 [M + H]^+^; 308.12640 [M + Na]^+^.

*Benzyl (S)-(1-hydroxy-3-phenylpropan-2-yl)carbamate* (**2b**): Colorless oil; yield 91%; R_f_ (hex/EtOAc–1/1) = 0.58. IR (ATR): 3329, 3065, 3035, 2952, 2871, 1686, 1586, 1530, 1466, 1453, 1384, 1333, 1288, 1263, 1225, 1172, 1079, 1007, 966, 750, 695 cm^−1^. ^1^H-NMR (400 MHz, CDCl_3_): δ 7.34–7.29 (5H, m, Ar-*H*), 5.11–5.02 (2H, ABq, *J* = 12 Hz, O-C*H_2_*-Ph), 4.89 (1H, s, N*H*-CH-CH_2_-CH-(CH_3_)_2_), 3.78–3.73 (1H, m, NH-C*H*-CH_2_-CH-(CH_3_)_2_), 3.64 (1H, dd, *J* = 3.6 Hz, *J* = 11.2, C*H*H-OH), 3.49 (1H, dd, *J* = 5.6 Hz, *J* = 10.8 Hz, CH*H*-OH), 2.64 (1H, t, *J* = 36 Hz, CH_2_-O*H*), 1.68–1.58 (1H, m, CH_3_-C*H*-CH_3_), 1.37–1.24 (2H, m, NH-CH-C*H_2_*-CH-(CH_3_)_2_), 0.90 (6H, d, *J* = 6.8 Hz, C*H_3_*-CH-C*H_3_* ). ^13^C-NMR (100 MHz, CDCl_3_): δ 157.0, 136.5, 128.7, 128.4, 128.3, 67.1, 66.2, 51.7, 40.7, 24.9, 23.2, 22.3. CHN analysis: Calc. for C_14_H_21_NO_3_ (251.32): C, 66.91; H, 8.42; N, 5.57. Found: C, 66.77 ± 0.04; H, 8.56 ± 0.01; N, 5.77 ± 0.01. HRMS: *m*/*z* calc. C_14_H_21_NO_3_: 252.15942 [M + H]^+^; 274.14136 [M + Na]^+^; found: 252.15975 [M + H]^+^; 274.14171 [M + Na]^+^.

*Benzyl (S)-(1-hydroxy-4-methylpentan-2-yl)carbamate* (**2c**): Faint yellow solid; yield 88%; mp 80–82 °C; R_f_ (hex/EtOAc–1/1) = 0.53. IR (ATR): 3451, 3322, 2978, 2960, 2906, 1726, 1691, 1658, 1540, 1499, 1473, 1332, 1251, 1131, 1083, 965, 748, 692 cm^−1^. ^1^H-NMR (400 MHz, CDCl_3_): δ 7.35–7.27 (5H, m, Ar-*H*), 5.06 (3H, t, *J* = 12.4 Hz, O-C*H_2_*-Ph, N*H*-CH-CH_3_), 3.83–3.76 (1H, m, NH-C*H*-CH_3_), 3.61 (1H, dd, *J* = 3.2 Hz, *J* = 11.2 Hz, C*H*H-OH), 3.47 (1H, dd, *J* = 5.6 Hz, *J* = 10.4 Hz, CH*H*-OH), 2.79 (1H, ABs, C*H_2_*-OH), 1.12 (3H, d, *J* = 6.8 Hz, NH-CH-C*H_3_*). ^13^C-NMR (100 MHz, CDCl_3_): δ 156.8, 136.5, 128.8, 128.7, 128.3, 67.0, 66.9, 49.2, 17.4. CHN analysis: Calc. for C_11_H_15_NO_3_ (209.24): C, 63.14; H, 7.23; N, 6.69. Found: C, 62.75 ± 1.85; H, 7.18 ± 0.24; N, 6.40 ± 0.20. HRMS: *m*/*z* calc. C_11_H_15_NO_3_: 210.11247 [M + H]^+^; 232.09441 [M + Na]^+^; found: 210.11275 [M + H]^+^; 332.09474 [M + Na]^+^.

#### 3.2.2. General Experimental Procedure for the Synthesis of Mesylates **3a–c**

To the solution of compound **2** (1.6 g, 5.60 mmol) in DCM, Et_3_N (0.680 g, 6.72 mmol) was added. The reaction mixture was cooled down to 0 °C; then methane sulfonyl chloride (0.770 g, 6.72 mmol) was added dropwise; and the reaction mixture was stirred at room temperature for 24 h. The solution was washed successively with 5% citric acid (10 mL), water (10 mL) and brine (10 mL). The organic layer was dried over Na_2_SO_4_ and concentrated. The crude product was purified by column chromatography using *n*-hexane:ethyl acetate (3:1) as eluent to obtain the products in 81–90% yield. 

*(S)-2-{[(benzyloxy)carbonyl]amino}propyl methanesulfonate* (**3a**): White solid; yield 90%; mp 120–123 °C; R_f_ (hex/EtOAc–1/1) = 0.58. IR (ATR): 3339, 3063, 3026, 2943, 1692, 1602, 1533, 1494, 1452, 1440, 1346, 1270, 1241, 1182, 985, 850, 745, 698 cm^−1^. ^1^H-NMR (400 MHz, CDCl_3_): δ 7.32–7.26 (7H, m, Ar-*H*), 7.22–7.20 (1H, m, Ar-*H*), 7.15 (2H, d, *J* = 7.2 Hz, Ar-*H*), 5.03 (3H, s, O-C*H_2_*-Ph, N*H*-CH-CH_2_-Ph), 4.22–4.19 (1H, m, NH-C*H*-CH_2_-Ph), 4.12–4.06 (2H, m, C*H_2_*-O-SO_2_-CH_3_), 2.89 (3H, s, SO_2_-C*H_3_*), 2.87–2.79 (2H, m, NH-CH-C*H_2_*-Ph). ^13^C- NMR (100 MHz, CDCl_3_): δ 155.9, 136.5, 136.4, 129.4, 129.0, 128.8, 128.5, 128.3, 128.3, 69.7, 67.1, 51.6, 37.4, 37.3. CHN analysis: Calc. for C_18_H_21_NO_5_S (363.43): C, 59.49; H, 5.82; N, 3.85, S, 8.82. Found: C, 60.16 ± 0.04; H, 6.04 ± 0.02; N, 4.78 ± 0.07, S, 8.41 ± 0.29. HRMS: *m*/*z* calc. C_18_H_21_NO_5_S: 364.12132 [M + H]^+^; 386.10326 [M + Na]^+^; found: 364.12232 [M + H]^+^; 386.10433 [M + Na]^+^.

*(S)-2-{[(benzyloxy)carbonyl]amino}-3-phenylpropyl methanesulfonate* (**3b**): Colorless oil; yield 86%; R_f_ (hex/EtOAc–1/1) = 0.23. IR (ATR): 3027, 2957, 2934, 2871, 1723, 1511, 1468, 1452, 1356, 1332, 1251, 1168, 1065, 1027, 976, 896, 762, 669 cm^−1^. ^1^H-NMR (400 MHz, CDCl_3_): δ 7.34–7.28 (5H, m, Ar-*H*), 5.11–5.04 (2H, ABq, *J* = 12 Hz, O-C*H_2_*-Ph), 4.86 (1H, d, *J* = 8 Hz, N*H*-CH-CH_2_-CH-(CH_3_)_2_), 4.25 (1H, dd, *J* = 3.6 Hz, *J* = 10 Hz, C*H*H-O-SO_2_CH_3_), 4.13 (1H, dd, *J* = 4.4 Hz, *J* = 10.4 HZ, CH*H*-O-SO_2_-CH_3_), 4.01–3.94 (1H, s, NH-C*H*-CH_2_- CH-(CH_3_)_2_), 2.92 (3H, s, SO_2_-C*H_3_*), 1.69–1.61 (1H, m, CH_3_-C*H*-CH_3_), 1.47–1.40 (1H, m, NH-CH-C*H*H-CH-(CH_3_)_2_), 1.37–1.30 (1H, m, NH-CH-CH*H*-CH-(CH_3_)_2_), 0.91 (6H, d, *J* = 6.4 Hz, C*H_3_*-CH-C*H_3_*). ^13^C-NMR (100 MHz, CDCl_3_): δ 156.1, 136.5, 128.8, 128.4, 128.3, 71.5, 67.1, 48.7, 40.2, 37.4, 24.8, 23.1, 22.1. CHN analysis: Calc. for C_15_H_23_NO_5_S (329.41): C, 54.69; H, 7.04; N, 4.25, S, 9.73. Found: C, 55.00 ± 0.03; H, 7.14 ± 0.03; N, 4.02 ± 0.01, S, 8.94 ± 0.35. HRMS: *m*/*z* calc. C_15_H_23_NO_5_S: 330.13697 [M + H]^+^; 352.11891 [M + Na]^+^; 368.09285 [M + K]^+^; found: 330.13762 [M + H]^+^; 352.11966 [M + Na]^+^; 368.09367 [M + K]^+^.

*(S)-2-{[(benzyloxy)carbonyl]amino}-4-methylpentyl methanesulfonate* (**3c**): Faint yellow solid; yield 81%; mp 98–100 °C; R_f_ (hex/EtOAc–1/1) = 0.44. IR (ATR): 3370, 3034, 2991, 2942, 2849, 1689, 1655, 1519, 1469, 1452, 1342, 1243, 1169, 1081, 1055, 961, 931, 836, 750, 696 cm^−1^. ^1^H-NMR (400 MHz, CDCl_3_): δ 7.36–7.29 (5H, m, Ar-*H*), 5.07 (2H, s, O-C*H_2_*-Ph), 4.95 (1H, d, *J* = 6 Hz, N*H*-CH-CH_3_), 4.24–4.19 (1H, m, NH-C*H*-CH_3_), 4.13 (1H, dd, *J* = 4 Hz, *J* = 9.6 Hz, C*H*H-O-SO_2_-CH_3_), 4.02 (1H, d, *J* = 3.2 Hz, CH*H*-O-SO_2_-CH_3_), 2.94 (3H, s, SO_2_-C*H_3_*), 1.22 (3H, d, *J* = 6.8 Hz, NH-CH-C*H_3_*). ^13^C-NMR (100 MHz, CDCl_3_): δ 150.5, 131.1, 123.5, 123.2, 123.1, 66.6, 61.8, 41.0, 32.2, 12.0. CHN analysis: Calc. for C_12_H_17_NO_5_S (287.33): C, 50.16; H, 5.96; N, 4.87, S, 11.16. Found: C, 50.79 ± 0.11; H, 6.04 ± 0.01; N, 4.61 ± 0.07, S, 10.38 ± 0.05. HRMS: *m*/*z* calc. C_17_H_19_NO_3_: 310.07196 [M + Na]^+^; 326.04560 [M + K]^+^; found: 310.07250 [M + Na]^+^; 326.04649 [M + K]^+^.

#### 3.2.3. General Experimental Procedure for the Synthesis of Thioacetates **4a–c**

To a suspension of Cs_2_CO_3_ (0.550 g, 1.68 mmol) in DMF, thioacetic acid (0.232 g, 3.0 mmol) was added under an argon atmosphere; after some time, compound **3** (0.920 g, 2.53 mmol) was added in one portion to the reaction mixture. The mixture was stirred for overnight, during which the reaction flask was covered with aluminum foil. The reaction mixture was poured into distilled water and extracted with ethyl acetate (3 × 50 mL). The combined organic layers were washed with water (60 mL), NaHCO_3_(5% *w*/*w* 60 mL), and brine (30 mL) and dried over sodium sulfate. The crude product was purified using column chromatography (silica gel eluted with 25% ethyl acetate in *n*-hexane) to give compounds **4a–c** (yields: 75–90%).

*(S)-SO-(2-{[(benzyloxy)carbonyl]amino}propyl) ethane(thioperoxoate)* (**4a**): White solid; yield 87%; mp 86–89 °C; R_f_ (hex/EtOAc–1/1) = 0.46. IR (ATR): 3361, 3061, 2989, 2913, 1690, 1686, 1524, 1461, 1422, 1349, 1270, 1243, 1054, 959, 752, 698 cm^−1^. ^1^H-NMR (400 MHz, CDCl_3_): δ 7.35–7.25 (7H, m, Ar-*H*), 7.23–7.15 (3H, m, Ar-*H*), 5.05 (2H, s, O-C*H_2_*-Ph), 4.89 (1H, d, *J* = 8 Hz, N*H*-CH-CH_2_-Ph), 4.11–3.99 (1H, m, NH-C*H*-CH_2_-Ph), 3.06 (1H, dd, *J* = 4.8 Hz, *J* = 14 Hz, C*H*H-S), 2.98–2.89 (2H, m, NH-CH-C*H_2_*-Ph), 2.78 (1H, dd, *J* = 7.2 Hz, *J* = 13.6, CH*H*-S), 2.30 (3H, s, S-CO-C*H_3_*). ^13^C-NMR (100 MHz, CDCl_3_): δ 196.0, 156.0, 146.5, 137.3, 129.5, 128.8, 128.7, 128.3, 128.2, 127.0, 66.8, 52.7, 40.6, 32.9, 30.8. CHN analysis: Calc. for C_19_H_21_NO_3_S (343.44): C, 66.45; H, 6.16; N, 4.08, S, 9.34. Found: C, 66.38 ± 0.13; H, 6.39 ± 0.04; N, 3.72 ± 0.01, S, 9.00 ± 0.13. HRMS: *m*/*z* calc. C_19_H_21_NO_3_S: 344.13149 [M + H]^+^; 366.11344 [M + Na]^+^; 382.08737 [M + K]^+^; found: 344.13237 [M + H]^+^; 366.11438 [M + Na]^+^; 382.08841 [M + K]^+^.

*(S)-SO-(2-{[(benzyloxy)carbonyl]amino}-3-phenylpropyl) ethane(thioperoxoate)* (**4b**): Brown solid; yield 90%; mp 56–58 °C; R_f_ (hex/EtOAc–1/1) = 0.62. IR (ATR): 3347, 3063, 2958, 2912, 2843, 1683, 1650, 1530, 1466, 1330, 1289, 1272, 1249, 1123, 1100, 1032, 952, 744, 696 cm^−1^. ^1^H-NMR (400 MHz, CDCl_3_): δ 7.33–7.28 (5H, m, Ar-*H*), 5.11–5.02 (2H, ABq, *J* = 12.4, Hz, O-C*H_2_*-Ph), 4.64 (1H, d, *J* = 8.4 Hz, N*H*-CH-CH_2_-CH-(CH_3_)_2_), 3.92–3.84 (1H, m, NH-C*H*-CH_2_-CH-(CH_3_)_2_), 3.09 (1H, dd, *J* = 4.8 Hz, *J* = 14 Hz, C*H*H-S), 2.96 (1H, dd, *J* = 7.2 Hz, *J* = 13.6 Hz, CH*H*-S), 2.28 (3H, s, S- CO-C*H_3_*), 1.68–1.58 (1H, m, CH_3_-C*H*-CH_3_), 1.39–1.23 (2H, m, NH-CH-C*H_2_*-CH-(CH_3_)_2_), 0.89 (6H, d, *J* = 6.4 Hz, C*H_3_*-CH-C*H_3_*). ^13^C- NMR (100 MHz, CDCl_3_): δ 196.0, 156.2, 136.7, 128.7, 128.3, 128.2, 66.8, 49.5, 43.8, 34.4, 30.8, 25.1, 23.2, 22.3. CHN analysis: Calc. for C_16_H_23_NO_3_S (309.42): C, 62.11; H, 7.49; N, 4.53, S, 10.36. Found: C, 62.54 ± 0.12; H, 7.79 ± 0.03; N, 4.50 ± 0.02, S, 9.53 ± 0.13. HRMS: *m*/*z* calc. C_16_H_23_NO_3_S: 310.14714 [M + H]^+^; 332.12909 [M + Na]^+^; 348.10302 [M + K]^+^; found: 310.14768 [M + H]^+^; 332.12966 [M + Na]^+^; 348.10358 [M + K]^+^.

*(S)-SO-(2-{[(benzyloxy)carbonyl]amino}-4-methylpentyl) ethane(thioperoxoate)* (**4c**): Dark brown solid; yield 75%; mp 64–65 °C; R_f_ (hex/EtOAc–1/1) = 0.38. IR (ATR): 3311, 3056, 3035, 2977, 2926, 1679, 1532, 1466, 1453, 1327, 1262, 1117, 1075, 964, 775, 696 cm^−1^. ^1^H-NMR (400 MHz, CDCl_3_): δ 7.36–7.28 (5H, m, Ar-*H*), 5.10 (2H, s, O-C*H_2_*-Ph), 4.90 (1H, s, N*H*-CH-CH_3_), 3.93 (1H, d, *J* = 6 Hz, NH-C*H*-CH_3_), 3.06 (2H, s, C*H_2_*-S), 2.34 (3H, s, S-CO-C*H_3_*), 1.21 (3H, d, *J* = 6.4 Hz, NH-CH-C*H_3_*). ^13^C-NMR (100 MHz, CDCl_3_): δ 195.9, 155.9, 136.7, 128.7, 128.3, 128.3, 66.8, 47.3, 35.1, 30.8, 20.3. CHN analysis: Calc. for C_13_H_17_NO_3_S (267.34): C, 58.40; H, 6.41; N, 5.24, S, 11.99. Found: C, 58.49 ± 0.01; H, 6.54 ± 0.02; N, 4.14 ± 0.03; S, 13.61 ± 0.05. HRMS: *m*/*z* calc. C_13_H_17_NO_3_S: 290.08214 [M + Na]^+^; found: 290.08253 [M + Na]^+^.

#### 3.2.4. General Experimental Procedure for the Synthesis of the Investigated Sulfonates **5a–5n**

NCS (2.5 g, 19.3 mmol) was dissolved in a cooled (0 °C) mixture of HCl (2 M solution, 1.2 mL) and ACN (20 mL) and stirred for 30 min. Thioacetate 4 (1.5 g, 4.84 mmol) was dissolved in can, added to the reaction mixture, and stirred for 60 min at room temperature. ACN was evaporated, and crude product was dissolved in ethyl acetate, extracted with sat. NaHCO_3_ and brine and dried over sodium sulfate. The crude product was utilized immediately in the next step without purification.

Under an argon atmosphere, DIPEA (0.805 g, 6.23 mmol) and the primary amine (1.1 eq) were dissolved in DCM at 0 °C and stirred for 1 h. Sulfonyl chloride (1.6 g, 4.79 mmol) was dissolved in DCM and added to the reaction mixture flask at 0 °C. The reaction mixture was stirred for 24 h at room temperature and then extracted with water (3 × 50 mL). The collected organic phase was washed with brine and dried over sodium sulfate. The crude product was purified by column chromatography using *n*-hexane: ethyl acetate (3:1). Yields: 79–87%.

*Benzyl [(2S)-1-(benzylsulfamoyl)propan-2-yl]carbamate* (**5a**): White solid; yield 82%; mp 127–128 °C; R_f_ (hex/EtOAc–1/1) = 0.35. IR (ATR): 3288, 3066, 3031, 2987, 2932, 2874, 1722, 1687, 1659, 1606, 1575, 1541, 1495, 1472, 1461, 1359, 1334, 1301, 1257, 1134, 1100, 1072, 1052, 961, 894, 867, 748, 692 cm^−1^. ^1^H-NMR (500 MHz, CDCl_3_): δ 7.28–7.19 (10H, m, Ar-*H*), 5.47 (1H, s, N*H*-SO_2_), 5.08 (1H, d, *J* = 8 Hz, N*H*-CH-CH_3_), 5.03–4.97 (2H, ABq, *J* = 13 Hz, O-C*H_2_*-Ph), 4.20 (2H, d, *J* = 4.5 Hz, NH-C*H_2_*-Ph), 4.02–3.97 (1H, m, NH-C*H*-CH_3_), 3.05 (1H, dd, *J* = 8 Hz, *J* = 14.5 Hz, C*H*H-SO_2_), 2.83 (1H, dd, *J* = 4 Hz, *J* = 14 Hz, CH*H*-SO_2_), 1.12 (3H, d, *J* = 7 Hz, NH-CH-C*H_3_*). ^13^C-NMR (100 MHz, CDCl_3_): δ 156.3, 137.1, 136.3, 129.1, 129.0, 128.8, 128.4, 128.3, 128.3, 67.2, 58.0, 47.4, 43.8, 20.9. CHN analysis: Calc. for C_18_H_22_N_2_O_4_S (362.44): C, 59.65; H, 6.12; N, 7.73, S, 8.85. Found: C, 60.02 ± 0.73; H, 6.16 ± 0.03; N, 7.19 ± 0.04; S, 7.60 ± 0.18. HRMS: *m*/*z* calc. C_18_H_22_N_2_O_4_S: 385.11925 [M + Na]^+^; found: 385.12011 [M + Na]^+^.

*Benzyl [(2S)-1-(benzylsulfamoyl)-3-phenylpropan-2-yl]carbamate* (**5b**): White solid; yield 84%; mp 157–158 °C; R_f_ (hex/EtOAc–1/1) = 0.42. IR (ATR): 3304, 3063, 2927, 2855, 1692, 1662, 1602, 1586, 1539, 1495, 1453, 1353, 1321, 1299, 1261, 1131, 1082, 1044, 907, 867, 846, 746, 694 cm^−1^. ^1^H-NMR (400 MHz, CDCl_3_): δ 7.48–7.26 (15H, m, Ar-*H*), 5.65 (1H, t, J = 7.2 Hz, N*H*-SO_2_), 5.33 (1H, d, *J* = 8.4 Hz, N*H*-CH-CH_2_-Ph), 5.17 (2H, t, *J* = 6.8 Hz, O-C*H_2_*-Ph,), 4.39–4.31 (3H, m, NH-C*H*-CH_2_-Ph, NH-C*H_2_*-Ph), 3.27 (1H, dd, *J* = 7.6 Hz, *J* = 14 Hz, C*H*H-SO_2_), 3.13 (1H, dd, *J* = 3.6 Hz, *J* = 14 Hz, CH*H*-SO_2_), 2.95 (2H, dd, *J* = 7.2 Hz, *J* = 13.2 Hz, NH-CH-C*H_2_*-Ph). ^13^C-NMR (100 MHz, CDCl_3_): δ 156.6, 137.1, 136.5, 136.4, 129.5, 129.1, 129.0, 128.7, 128.4, 128.3, 128.3, 128.2, 127.3, 67.2, 55.5, 49.0, 47.4, 40.4. CHN analysis: Calc. for C_24_H_26_N_2_O_4_S (438.54): C, 65.73; H, 5.98; N, 6.39, S, 7.31. Found: C, 66.14 ± 0.15; H, 6.04 ± 0.02; N, 6.21 ± 0.01, S, 6.72 ± 0.03. HRMS: *m*/*z* calc. C_24_H_26_N_2_O_4_S: 461.15055 [M + Na]^+^; 477.12449 [M + K]^+^; found: 461.15168 [M + Na]^+^; 477.12564 [M + K]^+^.

*Benzyl [(2S)-1-(benzylsulfamoyl)-4-methylpentan-2-yl]carbamate* (**5c**): White solid; yield 86%; mp 74–76 °C; R_f_ (hex/EtOAc–1/1) = 0.51. IR (ATR): 3336, 3286, 3064, 3033, 2958, 2874, 1693, 1662, 1586, 1532, 1465, 1434, 1400, 1353, 1318, 1266, 1244, 1132, 1045, 1026, 967, 906, 838, 774, 695 cm^−1^. ^1^H-NMR (400 MHz, CDCl_3_): δ 7.34–7.27 (10H, m, Ar-*H*), 5.65 (1H, t, *J* = 6 Hz, N*H*-SO_2_), 5.05 (2H, t, *J* = 14 Hz, O-C*H_2_*-Ph), 4.98 (1H, d, *J* = 9.6 Hz, N*H*-CH-CH_2_-CH-(CH_3_)_2_), 4.25 (2H, d, *J* = 5.6 Hz, NH-C*H_2_*-Ph), 4.01–3.95 (1H, m, NH-C*H*-CH_2_-CH-(CH_3_)_2_), 3.04 (1H, dd, *J* = 8.8 Hz, *J* = 14.8 Hz, C*H*H-SO_2_), 2.84 (1H, dd, *J* = 3.2 Hz, *J* = 14.4 Hz, CH*H*-SO_2_), 1.60–1.53 (1H, m, CH_3_-C*H*-CH_3_), 1.42–1.35 (1H, m, NH-CH-C*H*H-CH-(CH_3_)_2_), 1.14–1.08 (1H, m, NH-CH-CH*H*-CH-(CH_3_)_2_), 0.82 (6H, dd, *J* = 6.4 Hz, *J* = 10.4 Hz, C*H_3_*-CH-C*H_3_*). ^13^C-NMR (100 MHz, CDCl_3_): δ 157.0, 137.2, 136.4, 129.1, 128.9, 128.7, 128.4, 128.2, 128.2, 67.2, 57.4, 47.5, 46.2, 43.6, 24.7, 23.1. CHN analysis: Calc. for C_21_H_28_N_2_O_4_S (404.52): C, 62.35; H, 6.98; N, 6.93, S, 7.93. Found: C, 62.23 ± 0.02; H, 7.12 ± 0.01; N, 6.57 ± 0.04, S, 6.76 ± 0.13. HRMS: *m*/*z* calc. C_21_H_28_N_2_O_4_S: 427.16620 [M + Na]^+^; 443.14014 [M + K]^+^; found: 427.16733 [M + Na]^+^; 443.14136 [M + K]^+^.

*Benzyl [(2S)-4-methyl-1-(phenylsulfamoyl)pentan-2-yl]carbamate* (**5d**): Thick yellow oil; yield 86%; R_f_ (hex/EtOAc–1/1) = 0.55. IR (ATR): 3378, 3206, 3087, 3028, 2963, 2921, 2865, 1687, 1647, 1598, 1522, 1495, 1411, 1334, 1296, 1233, 1148, 1055, 1029, 952, 911, 751, 694 cm^−1^. ^1^H-NMR (400 MHz, CDCl_3_): δ 7.74 (1H, s, N*H*-SO_2_), 7.34–7.12 (10H, m, Ar-*H*), 5.17–4.097 (3H, m, O-C*H_2_*-Ph, N*H*-CH-CH_2_-CH-(CH_3_)_2_), 4.23–4.17 (1H, m, NH-C*H*-CH_2_-CH-(CH_3_)_2_), 3.14 (2H, dd, *J* = 11.6 Hz, *J* = 14.4 Hz, C*H_2_*-SO_2_), 1.58–1.52 (1H, m, CH_3_-C*H*-CH_3_), 1.44–1.36 (1H, m, NH-CH-C*H*H-CH-(CH_3_)_2_), 1.21–1.15 (1H, m, NH-CH-CH*H*-CH-(CH_3_)_2_), 0.78 (6H, dd, *J* = 6.4 Hz, *J* = 24.4 Hz, C*H_3_*-CH-C*H_3_*). ^13^C-NMR (100 MHz, CDCl_3_): δ 157.5, 137.4, 136.2, 129.8, 128.8, 128.5, 128.3, 125.2, 120.4, 67.5, 55.2, 45.9, 43.9, 24.7, 22.6, 21.9. CHN analysis: Calc. for C_20_H_26_N_2_O_4_S (390.5): C, 61.52; H, 6.71; N, 7.17, S, 8.21. Found: C, 61.32 ± 0.04; H, 6.86 ± 0.01; N, 6.53 ± 0.02; S, 7.39 ± 0.36. HRMS: *m*/*z* calc. C_20_H_26_N_2_O_4_S: 413.15055 [M + Na]^+^; 429.12449 [M + K]^+^; found: 413.15153 [M + Na]^+^; 429.12554 [M + K]^+^.

*Benzyl [(2S)-4-methyl-1-(propylsulfamoyl)pentan-2-yl]carbamate* (**5e**): Faint yellow solid; yield 87%; mp 58–60 °C; R_f_ (hex/EtOAc–1/1) = 0.53. IR (ATR): 3333, 3278, 3064, 2960, 2873, 1689, 1662, 1537, 1455, 1402, 1318, 1267, 1246, 1129, 1024, 907, 733, 696 cm^−1^. ^1^H-NMR (400 MHz, CDCl_3_): δ 7.33–7.27 (5H, m, Ar-*H*), 5.20–5.05 (4H, m, O-C*H_2_*-Ph, N*H*-CH-CH_2_-CH-(CH_3_)_2_, N*H*-SO_2_), 4.16–4.10 (1H, m, NH-C*H*-CH_2_-CH-(CH_3_)_2_), 3.19–3.07 (2H, m, C*H_2_*-SO_2_), 2.99 (2H, t, *J* = 6.4 Hz, C*H_2_*-CH_2_-CH_3_), 1.69–1.61 (1H, m, CH_3_-C*H*-CH_3_).1,57–1.50 (3H, m, CH_2_-C*H_2_*-CH_3_, NH-CH-C*H*H-CH-(CH_3_)_2_), 1.42–1.35 (1H, m, NH-CH-CH*H*-CH-(CH_3_)_2_), 0.91 (9H, t, *J* = 6.4 Hz, C*H_3_*-CH-C*H_3_*, CH_2_-CH_2_-C*H_3_*). ^13^C- NMR (100 MHz, CDCl_3_): δ 156.8, 136.4, 128.7, 128.4, 128.2, 67.2, 56.3, 46.4, 45.2, 43.8, 24.9, 23.7, 23.0, 21.9, 11.3. CHN analysis: Calc. for C_17_H_28_N_2_O_4_S (356.48): C, 57.28; H, 7.92; N, 7.86, S, 8.99. Found: C, 57.35 ± 0.12; H, 8.04 ± 0.03; N, 7.47 ± 0.01, S, 7.19 ± 0.25. HRMS: *m*/*z* calc. C_17_H_28_N_2_O_4_S: 379.16620 [M + Na]^+^; 395.14014 [M + K]^+^; found: 379.16703 [M + Na]^+^; 395.14105 [M + K]^+^.

*Benzyl {(2S)-4-methyl-1-[(2-phenylethyl)sulfamoyl]pentan-2-yl}carbamate* (**5f**): White solid; yield 86%; mp 59–60 °C; R_f_ (hex/EtOAc–1/1) = 0.57. IR (ATR): 3378, 3271, 3065, 3031, 2959, 2931, 2876, 1698, 1603, 1584, 1519, 1452, 1438, 1399, 1358, 1344, 1316, 1266, 1245, 1133, 1113, 1067, 1054, 1021, 970, 903, 829, 804, 745, 695 cm^−1^. ^1^H-NMR (500 MHz, CDCl_3_): δ 7.27 (7H, t, *J* = 7 Hz, Ar-*H*), 7.20–7.14 (3H, m, Ar-*H*), 5.06–4.98 (4H, m, O-C*H_2_*-Ph, N*H*-CH-CH_2_-CH-(CH_3_)_2_), N*H*-SO_2_), 4.05–4.01 (1H, m, NH-C*H*-CH_2_-CH-(CH_3_)_2_), 3.28 (2H, q, *J* = 7 Hz, NH-C*H_2_*-CH_2_-Ph), 3.07 (1H, dd, *J* = 8 Hz, *J* = 14 Hz, C*H*H-SO2), 2.96 (1H, d, *J* = 12 Hz, CH*H*-SO_2_), 2.79 (2H, t, *J* = 6.5 Hz, NH-CH_2_-C*H_2_*-Ph), 1.62–1.57 (1H, m, CH_3_-C*H*-CH_3_), 1.49–1.43 (1H, m, NH-CH-C*H*H-CH-(CH_3_)_2_), 1.34–1.29 (1H, m, NH-CH-CH*H*-CH-(CH_3_)_2_), 0.85 (6H, d, *J* = 6 Hz, C*H_3_*-CH-C*H_3_*). ^13^C-NMR (100 MHz, CDCl_3_): δ 156.6, 138.1, 136.4, 129.1, 128.9, 128.8, 128.4, 128.2, 127.0, 67.2, 56.5, 46.4, 44.6, 43.5, 36.9, 24.9, 23.1, 21.9. CHN analysis: Calc. for C_22_H_30_N_2_O_4_S (418.55): C, 63.13; H, 7.22; N, 6.69, S, 7.66. Found: C, 63.30 ± 0.03; H, 7.25 ± 0.02; N, 6.51 ± 0.01, S, 7.09 ± 0.07. HRMS: *m*/*z* calc. C_22_H_30_N_2_O_4_S: 441.18185 [M + Na]^+^; found: 441.18306 [M + Na]^+^.

*Benzyl {(2S)-1-[(3,4-dichlorophenyl)sulfamoyl]-3-phenylpropan-2-yl}carbamate* (**5g**): Faint yellow solid; yield 83%; mp 154–156 °C; R_f_ (hex/EtOAc–1/1) = 0.48. IR (ATR): 3332, 3224, 3030, 2931, 1691, 1689, 1591, 1531, 1495, 1454, 1380, 1318, 1260, 1252, 1148, 1081, 1043, 958, 888, 738, 695 cm^−1^. ^1^H-NMR (400 MHz, CDCl_3_): δ 7.89 (1H, s, N*H*-SO_2_), 7.32 (7H, t, *J* = 6.8 Hz, Ar-*H*), 7.20 (3H, s, Ar-*H*), 7.05 (1H, d, *J* = 7.6 Hz, Ar-*H*), 6.97 (2H, d, *J* = 2.4 Hz, Ar-*H*), 5.16 (1H, d, *J* = 8.8 Hz, N*H*-CH-CH_2_-Ph), 5.09 (2H, s, O-C*H_2_*-Ph,), 4.37–4.31 (1H, m, NH-C*H*-CH_2_-Ph), 3.14 (2H, dd, *J* = 9.2 Hz, *J* = 14.4 Hz, C*H_2_*-SO_2_), 2.90 (1H, dd, *J* = 5.6 Hz, *J* = 13.6 Hz, NH-CH-C*H*H-Ph), 2.69 (1H, dd, *J* = 8 Hz, *J* = 13.6 Hz, NH-CH-CH*H*-Ph). ^13^C- NMR (100 MHz, CDCl_3_): δ 157.3, 136.8, 136.0, 135.9, 135.7, 133.6, 131.4, 129.1, 129.1, 128.8, 128.6, 128.3, 127.5, 121.6, 119.3, 67.7, 53.9, 48.7, 40.9. CHN analysis: Calc. for C_23_H_22_Cl_2_N_2_O_4_S (493.4): C, 55.99; H, 4.49; N, 5.68, S, 6.50. Found: C, 55.99 ± 0.44; H, 4.50 ± 0.02; N, 5.52 ± 0.02, S, 6.03 ± 0.20. HRMS: *m*/*z* calc. C_23_H_22_Cl_2_N_2_O_4_S: 515.05695 [M + Na]^+^; found: 515.05836 [M + Na]^+^.

*Benzyl [(2S)-1-(1,3-benzodioxol-5-ylsulfamoyl)-3-phenylpropan-2-yl]carbamate* (**5h**): Dark brown solid; yield 79%; mp 110–112 °C; R_f_ (hex/EtOAc–1/1) = 0.44. IR (ATR): 3366, 3171, 3087, 3061, 2928, 2895, 1688, 1635, 1586, 1528, 1500, 1484, 1416, 1340, 1315, 1257, 1238, 1146, 1079, 1038, 953, 935, 847, 832, 745, 697 cm^−1^. ^1^H-NMR (400 MHz, CDCl_3_): δ 7.37 (1H, s, N*H*-SO_2_), 7.33–7.27 (5H, m, Ar-*H*), 7.20 (3H, t, *J* = 7.2 Hz, Ar-*H*), 7.04 (2H, d, *J* = 6.4 Hz, Ar-*H*), 6.81 (1H, s, Ar-*H*), 6.66 (2H, d, *J* = 8 Hz, Ar-*H*), 5.95 (2H, s, O-C*H_2_*-O), 5.24 (1H, d, *J* = 8.8 Hz, N*H*-CH-CH_2_-Ph), 5.08 (2H, t, *J* = 12.8 Hz, O-C*H_2_*-Ph), 4.44–4.37 (1H, m, NH-C*H*-CH_2_-Ph), 3.14 (2H, dd, *J* = 10.8 Hz, *J* = 18.8, C*H_2_*-SO_2_), 2.92 (1H, dd, *J* = 6.4 Hz, *J* = 13.6 Hz, NH-CH-C*H*H-Ph), 2.77 (1H, dd, *J* = 7.6 Hz, *J* = 14 Hz, NH-CH-CH*H*-Ph). ^13^C- NMR (100 MHz, CDCl_3_): δ 157.0, 148.5, 145.8, 136.2, 130.8, 129.3, 129.0, 128.8, 128.4, 128.2, 127.3, 115.2, 108.6, 104.3, 101.7, 67.4, 53.5, 48.7, 40.7, 14.4. CHN analysis: Calc. for C_24_H_24_N_2_O_6_S (468.52): C, 61.52; H, 5.16; N, 5.98, S, 6.84. Found: C, 61.79 ± 0.15; H, 5.29 ± 0.03; N, 5.80 ± 0.01, S, 6.43 ± 0.02. HRMS: *m*/*z* calc. C_24_H_24_N_2_O_6_S: 491.12473 [M + Na]^+^; 507.09867 [M + K]^+^; found: 491.12597 [M + Na]^+^; 507.10023 [M + K]^+^.

*Benzyl {(2S)-1-phenyl-3-[(2S)-tetrahydrofuran-2-ylsulfamoyl]propan-2-yl}carbamate* (**5i**): White solid; yield 80%; mp 132–134 °C; R_f_ (hex/EtOAc–1/1) = 0.23. IR (ATR): 3347, 3288, 3058, 2975, 2861, 1689, 1658, 1587, 1530, 1448, 1327, 1308, 1292, 1145, 1120, 1051, 1040, 924, 906, 745, 629 cm^−1^. ^1^H-NMR (400 MHz, CDCl_3_): δ 7.23–7.15 (8H, m, Ar-*H*), 7.10 (2H, d, *J* = 6.8 Hz, Ar-*H*), 5.28 (2H, t, *J* = 7.6 Hz, N*H*-CH-CH_2_-Ph, N*H*-SO_2_) 5.00 (2H, s, O-C*H_2_*-Ph), 4.25–4.20 (1H, m, NH-C*H*-CH_2_-Ph), 3.82 (2H, dd, *J* = 7.2 Hz, *J* = 16 Hz, THF-CH, C*H*H-SO_2_), 3.64 (2H, t, *J* = 6 Hz, THF), 3.51 (1H, d, *J* = 7.2 Hz, CH*H*-SO_2_), 3.17 (1H, dd, *J* = 8 Hz, *J* = 14 Hz, NH-CH-C*H*H-Ph), 3.06 (1H, d, *J* = 12 Hz, NH-CH-CH*H*-Ph), 2.95 (1H, q, *J* = 5.2 Hz, THF), 2.83 (1H, q, *J* = 6.8 Hz, THF), 2.11–2.02 (1H, m, THF), 1.79–1.78(1H, m, THF). ^13^C-NMR (100 MHz, CDCl_3_): δ 156.4, 136.6, 136.3, 129.5, 129.1, 128.8, 128.4, 128.2, 127.4, 73.5, 67.2, 66.8, 55.6, 54.1, 49.4, 40.5, 33.7. CHN analysis: Calc. for C_21_H_26_N_2_O_5_S (418.51): C, 60.27; H, 6.26; N, 6.69, S, 7.66. Found: C, 60.53 ± 0.26; H, 6.38 ± 0.02; N, 6.54 ± 0.02, S, 7.33 ± 0.40. HRMS: *m*/*z* calc. C_21_H_26_N_2_O_5_S: 441.14546 [M + Na]^+^; 457.11940 [M + K]^+^; found: 441.14682 [M + Na]^+^; 457.12082 [M + K]^+^.

*Benzyl {(2S)-1-[(4-chlorobenzyl)sulfamoyl]-4-methylpentan-2-yl}carbamate* (**5j**): White solid; yield 84%; mp 104–106 °C; R_f_ (hex/EtOAc–1/1) = 0.57. IR (ATR): 3346, 3287, 3064, 3036, 2962, 2919, 2871, 1688, 1577, 1532, 1489, 1453, 1403, 1340, 1318, 1276, 1247, 1129, 1122, 1106, 1094, 1039, 1027, 996, 948, 870, 860, 734, 694 cm^−1^. ^1^H-NMR (500 MHz, CDCl_3_): δ 7.37–7.28 (9H, m, Ar-*H*), 5.76 (1H, t, *J* = 6.5 Hz, N*H*-SO_2_), 5.12–5.05 (3H, m, O-C*H_2_*-Ph, N*H*-CH-CH_2_-CH-(CH_3_)_2_), 4.24 (2H, d, *J* = 5.5 Hz, NH-C*H_2_*-(4-Cl)-Ph), 4.05–3.99 (1H, m, NH-C*H*-CH_2_-CH-(CH_3_)_2_), 3.11 (1H, dd, *J* = 9 Hz, *J* = 14.5 Hz, C*H*H-SO_2_), 2.92 (1H, d, *J* = 14.5 Hz, CH*H*-SO_2_), 1.66–1.58 (1H, m, NH-CH-C*H*H-CH-(CH_3_)_2_), 1.48–1.43 (1H, m, NH-CH-CH*H*-CH-(CH_3_)_2_), 1.23–1.18 (1H, m, CH_3_-C*H*-CH_3_), 0.88 (6H, dd, *J* = 7 Hz, *J* = 12.5 Hz, C*H_3_*-CH-C*H_3_*). ^13^C-NMR (100 MHz, CDCl_3_): δ 156.8, 136.1, 135.7, 133.9, 129.5, 129.0, 128.6, 128.3, 128.0, 67.1, 57.2, 46.5, 46.0, 43.5, 24.6, 22.9, 21.6. CHN analysis: Calc. for C_21_H_27_ClN_2_O_4_S (438.97): C, 57.46; H, 6.20; N, 6.38, S, 7.30. Found: C, 57.80 ± 0.13; H, 6.20 ± 0.02; N, 6.14 ± 0.01, S, 5.86 ± 0.43. HRMS: *m*/*z* calc. C_21_H_27_ClN_2_O_4_S: 461.12723 [M + Na]^+^; 477.10116 [M + K]^+^; found: 461.12857 [M + Na]^+^; 477.10260 [M + K]^+^.

*Benzyl {(2S)-1-[(2-methoxybenzyl)sulfamoyl]-4-methylpentan-2-yl}carbamate* (**5k**): Thick oil; yield 87%; R_f_ (hex/EtOAc–1/1) = 0.50. IR (ATR): 3340, 3065, 3033, 2955, 2869, 2839, 1698, 1602, 1589, 1521, 1494, 1463, 1438, 1413, 1326, 1290, 1257, 1141, 1118, 1072, 1025, 956, 936, 872, 836, 752, 696 cm^−1^. ^1^H-NMR (500 MHz, CDCl_3_): δ 7.32–7.25 (7H, m, Ar-*H*), 6.89 (2H, q, *J* = 7.5 Hz, Ar-*H*), 5.64 (1H, s, N*H*-SO_2_), 5.06 (2H, t, *J* = 14 Hz, O-C*H_2_*-Ph), 4.93 (1H, d, *J* = 9 Hz, N*H*-CH-CH_2_-CH-(CH_3_)_2_), 4.31–4.23 (2H, m, NH-C*H*-CH_2_-CH-(CH_3_)_2_, NH-C*H*H-(2-O-CH_3_)-Ph), 3.85 (4H, s, NH-CH_2_-(2-O-C*H_3_*)-Ph, NH-CH*H*-(2-O-CH_3_)-Ph), 2.95 (1H, dd, *J* = 9 Hz, *J* = 14.5 Hz, C*H*H-SO_2_), 2.82 (1H, d, *J* = 12.5 Hz CH*H*-SO_2_), 1.58–1.50 (1H, m, CH_3_-C*H*-CH_3_). 1.40–1.34 (1H, m, NH-CH-C*H*H-CH-(CH_3_)_2_), 1.07–1.02 (1H, m, NH-CH-CH*H*-CH-(CH_3_)_2_), 0.79 (6H, dd, *J* = 6.5 Hz, *J* = 20 Hz, C*H_3_*-CH-C*H_3_*). ^13^C-NMR (100 MHz, CDCl_3_): δ 157.7, 156.6, 136.5, 130.1, 129.9, 128.7, 128.3, 128.2, 125.4, 120.9, 110.8, 67.1, 57.4, 55.6, 46.1, 43.9, 43.5, 24.7, 23.1, 21.9. CHN analysis: Calc. for C_22_H_30_N_2_O_5_S (434.55): C, 60.81; H, 6.96; N, 6.45, S, 7.38. Found: C, 60.75 ± 0.20; H, 7.19 ± 0.02; N, 6.07 ± 0.04, S, 6.17 ± 0.09. HRMS: *m*/*z* calc. C_22_H_30_N_2_O_5_S: 457.17676 [M + Na]^+^; found: 457.17802 [M + Na]^+^.

*Benzyl {(2S)-1-[(3,4-difluorobenzyl)sulfamoyl]-4-methylpentan-2-yl}carbamate* (**5l**): White solid; yield 86%; mp 70–72 °C; R_f_ (hex/EtOAc–1/1) = 0.47. IR (ATR): 3338, 3294, 3066, 3037, 2967, 2941, 2871, 1690, 1609, 1536, 1519, 1452, 1425, 1322, 1285, 1228, 1202, 1174, 1133, 1093, 1035, 1007, 996, 952, 894, 772.733 694 cm^−1^. ^1^H-NMR (500 MHz, CDCl_3_): δ 7.29 (5H, d, *J* = 14.5 Hz, Ar-*H*), 7.18–7.04 (3H, m, Ar-*H*), 5.73 (1H, t, *J* = 8 Hz, N*H*-SO_2_), 5.09–5.01 (3H, m, O-C*H_2_*-Ph, N*H*-CH-CH_2_-CH-(CH_3_)_2_), 4.17 (2H, d, *J* = 3.5 Hz, NH-C*H_2_*-(3,4-di-F)-Ph), 4.07–4.02 (1H, m, NH-C*H*-CH_2_-CH-(CH_3_)_2_), 3.11 (1H, dd, *J* = 9 Hz, *J* = 14.5 Hz, C*H*H-SO_2_), 2.94 (1H, d, *J* = 13.5 Hz, CH*H*-SO_2_), 1.63–1.56 (1H, m, NH-CH-C*H*H-CH-(CH_3_)_2_), 1.48–1.43 (1H, m, NH-CH-CH*H*-CH-(CH_3_)_2_) 1.26–1.21 (1H, m, CH_3_-C*H*-CH_3_), 0.86 (6H, t, *J* = 7.5 Hz, C*H_3_*-CH-C*H_3_*). ^13^C-NMR (100 MHz, CDCl_3_): δ 157.0, 151.6 (dd, *J* = 37.8 Hz, *J* = 12.6 Hz), 149.1 (dd, *J* = 36.8, *J* = 12.5 Hz), 136.3, 134.5(dd, *J* = 8.2 Hz, *J* = 5.3 Hz), 128.8, 128.5, 128.2, 124.2 (dd, *J* = 6 Hz, *J* = 3.1 Hz), 117.7 (d, *J* = 17.2 Hz), 117.2 (d, *J* = 17.5 Hz), 67.3, 57.4, 46.3, 46.3, 43.8, 24.8, 23.0, 21.7. ^19^F NMR (377 MHz, CDCl_3_): −136.6 (d, *J* = 18.9 Hz), −138.7 (d, *J* = 22.6 Hz), CHN analysis: Calc. for C_21_H_26_F_2_N_2_O_4_S (440.5): C, 57.26; H, 5.95; N, 6.36, S, 7.28. Found: C, 57.49 ± 0.07; H, 6.16 ± 0.02; N, 5.96 ± 0.01, S, 6.35 ± 0.43. HRMS: *m*/*z* calc. C_21_H_26_F_2_N_2_O_4_S: 441.16541 [M + H]^+^; 463.14736 [M + Na]^+^; found: 441.16669 [M + H]^+^; 463.14872 [M + Na]^+^.

*Benzyl {(2S)-1-[(furan-2-ylmethyl)sulfamoyl]-4-methylpentan-2-yl}carbamate* (**5m**): Light yellow solid; yield 80%; mp 80–82 °C; R_f_ (hex/EtOAc–1/1) = 0.55. IR (ATR): 3304, 3125, 3066, 2952, 2931, 2871, 1686, 1656, 1540, 1503, 1452, 1429, 1313, 1296, 1265, 1230, 1189, 1136, 1116, 1058, 1043, 959, 922, 866, 809, 743, 693 cm^−1^. ^1^H-NMR (500 MHz, CDCl_3_): δ 7.35–7.24 (6H, m, Ar-*H*), 6.29 (2H, d, *J* = 12 Hz, Ar-*H*), 5.82 (1H, t, *J* = 7 Hz, N*H*-SO_2_), 5.10–5.04 (2H, ABq, *J* = 12 Hz, O-C*H_2_*-Ph), 4.94 (1H, d, *J* = 9.5 Hz, N*H*-CH-CH_2_-CH-(CH_3_)_2_), 4.34–4.22 (2H, m, NH-C*H_2_*-furan), 4.03–3.99 (1H, m, NH-C*H*-CH_2_-CH-(CH_3_)_2_), 3.00 (1H, dd, *J* = 9.5 Hz, *J* = 14.5 Hz, C*H*H-SO_2_), 2.88 (1H, d, *J* = 13.5 Hz, CH*H*-SO_2_), 1.64–1.56 (1H, m, CH_3_-C*H*-CH_3_), 1.42–1.36 (1H, m, NH-CH-C*H*H-CH-(CH_3_)_2_), 1.16–1.11 (1H, m, NH-CH-CH*H*-CH-(CH_3_)_2_), 0.84 (6H, dd, *J* = 6.5 Hz, *J* = 11.5 Hz, C*H_3_*-CH-C*H_3_*). ^13^C-NMR (100 MHz, CDCl_3_): δ 157.0, 150.6, 142.8, 136.2, 128.5, 128.2, 128.0, 110.7, 108.7, 67.1, 57.5, 45.9, 43.5, 39.8, 24.5, 23.0, 21.5. CHN analysis: Calc. for C_19_H_26_N_2_O_5_S (394.49): C, 57.85; H, 6.64; N, 7.10, S, 8.13. Found: C, 57.79 ± 0.07; H, 6.56 ± 0.05; N, 7.01 ± 0.01, S, 7.53 ± 0.29. HRMS: *m*/*z* calc. C_19_H_26_N_2_O_5_S: 417.14546 [M + Na]^+^; 433.11940 [M + K]^+^; found: 417.14653 [M + Na]^+^; 433.12052 [M + K]^+^.

*Benzyl {(2S)-1-[(3,4-dichlorophenyl)sulfamoyl]-4-methylpentan-2-yl}carbamate* (**5n**): White solid; yield 85%; mp 117–118 °C; R_f_ (hex/EtOAc–1/1) = 0.57. IR (ATR): 3342, 3214, 3067, 2954, 2872, 1692, 1591, 1537, 1473, 1380, 1337, 1271, 1130, 1078, 1050, 955, 887, 853, 775, 693 cm^−1^. ^1^H-NMR (400 MHz, CDCl_3_): δ 8.05 (1H, s, N*H*-SO_2_), 7.46 (1H, s, Ar-*H*), 7.34 (6H, d, *J* = 12.4 Hz, Ar-*H*), 7.16 (1H, d, *J* = 7.6 Hz, Ar-*H*), 5.16–5.03 (3H, m, O-C*H_2_*-Ph, N*H*-CH-CH_2_-CH-(CH_3_)_2_), 4.21–4.12 (1H, m, NH-C*H*-CH_2_-CH-(CH_3_)_2_), 3.13 (2H, d, *J* = 6.4 Hz, C*H_2_*-SO_2_), 1.60–1.52 (1H, m, CH_3_-C*H*-CH_3_), 1.44–1.37 (1H, m, NH-CH-C*H*H-CH-(CH_3_)_2_), 1.23–1.16 (1H, m, NH-CH-CH*H*-CH-(CH_3_)_2_), 0.81 (6H, dd, *J* = 6.4 Hz, *J* = 16 Hz, C*H_3_*-CH-C*H_3_*). ^13^C-NMR (100 MHz, CDCl_3_): δ 157.6, 137.0, 136.0, 133.7, 131.4, 128.9, 128.8, 128.6, 128.2, 122.0, 119.7, 67.7, 55.7, 45.9, 43.8, 24.7, 22.6, 21.7. CHN analysis: Calc. for C_20_H_24_Cl_2_N_2_O_4_S (459.39): C, 52.29; H, 5.27; N, 6.10, S, 6.98. Found: C, 52.87 ± 0.05; H, 5.35 ± 0.02; N, 5.88 ± 0.02, S, 7.57 ± 0.07. HRMS: *m*/*z* calc. C_20_H_24_Cl_2_N_2_O_4_S: 481.07260 [M + Na]^+^; 497.04654 [M + K]^+^; found: 481.07371 [M + Na]^+^; 497.04772 [M + K]^+^.

### 3.3. AChE and BChE Inhibition Studies

The ability of all synthesized derivatives to inhibit eeAChE (acetylcholinesterase from electric eel, *Electrophorus electricus*) and eqBChE (butyrylcholinesterase from equine serum) was determined in vitro using the modified Ellman´s method. The inhibitory activity of the studied compounds was expressed as IC_50_ value representing the concentration of an inhibitor, which is necessary for the reduction of enzyme activity (or reaction rate) to 50%. Ellmanʼs method [57] is widely used for measuring cholinesterase activity and the efficiency of cholinesterase inhibitors. The principle of this simple method is the determination of the SH and -S-S- groups [85]. The activity of the cholinesterase is measured indirectly by quantifying the concentration of 2-nitro-5-sulfanylbenzoic acid ion formed in the reaction between 5,5′-dithiobis-2-nitrobenzoic acid (DTNB) and thiocholine (i.e., the product of the acetylthiocholine hydrolysis catalysed by cholinesterase).

All studied compounds were dissolved in DMSO (concentration 0.01 M) and diluted in demineralized water (concentration 0.001 M). The ability of the studied compounds to inhibit eeAChE and eqBChE was determined using modified Ellman´s method at 25 °C in the presence of phosphate buffered saline (PBS, 0.1 M, pH 7.4) in a glass cuvette with 1 cm optical path. The enzyme activity in the total reaction mixture (2 mL) was 0.2 U/mL; the concentration of acetylthiocholine (ATCh) or butyrylthiocholine (BTCh) was 40 μM; and the concentration of DTNB was 0.1 mM for all reactions. The inhibitory activity of the studied derivatives was evaluated based on the ratio *v*_0_/*v*_i_ (*v*_0_ is the rate of ATCh or BTCh hydrolysis in the absence of the inhibitor, *v*_i_ is the rate of ATCh or BTCh hydrolysis in the presence of the inhibitor). The IC_50_ value was obtained from the dependence v_0_/v_i_ on the concentration of the tested compound (inhibitor).

The determination of v_0_ was done as follows. Into the cuvette, PBS (0.1 M, pH 7.4), DTNB, and ATCh (BTCh) were placed. The enzymatic reaction was started by adding the enzyme. The dependence of absorbance (λ = 412 nm) on the time was observed for 70 s (the reference solution contained PBS, DTNB, and ATCh or BTCh), and then the reaction rate (v_0_) was calculated (v = ΔA/Δt). The measurement was performed at least in triplicate, and average v_0_ was determined.

Then, *v*_i_ (for the given concentration of inhibitor) was determined. Into the cuvette, DTNB, ATCh (BTCh), a chosen volume of the suitably diluted inhibitor (to achieve the required concentration of inhibitor in the total reaction mixture), and a certain volume of PBS (to achieve the total volume of the reaction mixture 2 mL after adding the enzyme) were placed. The enzymatic reaction was started by adding the enzyme. The dependence of absorbance (λ = 412 nm) on time was observed for 70 s (the reference solution was the same as for the reaction in the absence of the inhibitor), and then the reaction rate (*v*_i_) was calculated. Five different concentrations of the inhibitor were used, and each measurement was performed at least in duplicate.

Finally, the dependence *v*_0_/*v*_i_ on the concentration of the inhibitor was determined, and IC_50_ was calculated from the obtained equation of the regression curve for y = 2 (based on the definition of IC_50_).

### 3.4. Kinetic Studies

Two derivatives (compounds **5k**, **5j**) were used for evaluation of carbamylation and decarbamylation of BChE from equine serum. The decrease in enzyme activity over time due to inhibitor binding was monitored by the Ellman´s method. Pursuant the procedure described in Carletti [86], the determination was performed subsequently: the reaction mixture containing PBS (0.1 M, pH 7.4), BChE and chosen inhibitor in an appropriate concentration was prepared and intensively stirred. In given times DTNB and BTCh were added to the sample withdrawn from stirred reaction mixture, quickly mixed and absorbance was measured. Consequently, the enzyme activity was determined. Based on knowledge of enzyme activity in the absence of the inhibitor (i.e., 100% activity), the percentages of residual activity in presence of the inhibitor were calculated. Then the dependence of percentage of residual activity vs. time was constructed [87]. By nonlinear regression of these dependences the values of *k_obs_* were obtained and subsequently rate constants for carbamylation were calculated. All experiments were performed in duplicate at least.

Monitoring of enzyme reactivation was performed as follows. BChE was inhibited ≥ 95%. The excess inhibitor was removed by dilution into PBS. Samples were withdrawn at successive time points and assayed to measure recovery of enzymatic activity (Ellman´s method was used). The percentage of reactivation was determined by comparison with that for control samples, where the BChE had been mixed only with buffer, but otherwise treated identically. All experiments were performed in duplicate at least.

### 3.5. Molecular Modeling

#### 3.5.1. Receptor Preparation and Docking Procedure

X-ray enzyme structures available at the Protein Data Bank were used as follows: *Torpedo californica* AChE code 1DX6 [88] and *Equus caballus* BChE (UniPrtoAC Q9N1N9). Water and ligands molecules were removed from PDB structure before calculations. Receptor structure as well as sulfonamide derivative structures were converted from pdb to pdbqt format using AutoDockTools 1.5.4 [88]. Gasteiger charges were added for all the compounds, and nonpolar hydrogen atoms were merged. Molecular docking studies were performed using AutoDock4 software [88]. The receptor structure was defined as rigid. The XYZ dimensions of a cubic grid were set to 60 × 60 × 60 points, repectively, with a spacing resolution of 0.375 Å and centered at the catalytic site of each enzyme. All torsions of the ligand were allowed to rotate during docking. Other parameters were set to default values. 200 poses were collected and then clustered into families based on the root mean square deviation (RMSD) between the Cartesian coordinates of the ligand atoms. A representative structure from the most populated cluster was selected for further studies.

#### 3.5.2. Molecular Dynamic Simulations

MD simulations were performed using the Amber16 [89] software package considering ff99SB [90] and GAFF [91] force fields. Calculations were carried out in triplicate for all complexes obtained under docking procedures. Each model was soaked in a truncated octahedral periodic box of TIP3P water molecules [92] with a margin of 10.0 Å in each direction from the solute. Na^+^ or Cl^-^ ions were placed by the Leap module to neutralize the charges of AChE and BChE complexes, respectively. The potential energy of the complexes was then minimized using the Sander module in 5000 steps with a steepest-descent algorithm. Subsequently, complexes were equilibrated at constant volume for 500 ps. Each system was heated from 0 to 300 K using a Langevin thermostat [93]. The SHAKE algorithm [94] was applied allowing for an integration time step of 2 fs. The equilibration run was followed by three 20 ns MD runs without position restraints under periodic boundary conditions at the target temperature 298 K. The particle mesh Ewald method (PME) [95] was employed using a grid spacing of 1.2 Å, a spline interpolation order of 4, and a real space direct sum cutoff of 8 Å. Post MD analysis was performed using the program CPPTRAJ [96].

#### 3.5.3. MM-GBSA Free Energy Decomposition

The MM-GBSA free energy decomposition using the mm_pbsa module in Amber16 was employed to corroborate the amino acids from each enzyme catalytic site interacting with the ligands. The explicit water molecules and counter ions were removed and the equidistant snapshots extracted from the last 10 ns of the dynamics in triplicate were considered.

#### 3.5.4. QTAIM Analysis

In order to select the most stable or probable conformation for QTAIM analysis [49], a clustering process using the CPPTRAJ program (AmberTools package) was carried out. This technique is based on the RMSA of the L-R complex. An RMSD of 2 Å was considered. The representative structure of the most populated cluster for each complex was employed as the input structure.

Charge density topological analysis based in the QTAIM was performed on a BChE-5c and BChE-5k reduced models to evaluate the L-R interactions. The reduced model was constructed by considering those residues that directly interact with the ligands. All amino acids found within a radius of 5 Å of the distance from each ligand atom were included.

The wave function for the reduced models generated at the M062X/6-31G(d) level of theory were computed with the Gaussian16 [97] package and were subjected to quantum theory atoms in molecules (QTAIM) analysis using the Multiwfn software [98]. Molecular graphs were depicted with Pymol.

QTAIM calculations were performed in order to determine the ρ(r) values at the bond critical points (BCPs). Results are summarized as the sum of the ρ(r) values of BCPs considering the amino acid residues belonging to a particular binding site of BChE.

### 3.6. Cytotoxicity Evaluation

Human monocytic leukemia THP-1 cells were obtained from the European Collection of Cell Cultures (ECACC, Salisbury, UK) and routinely cultured in RPMI 1640 medium supplemented with 10% fetal bovine serum (FBS), 2% l-glutamine, 1% penicillin, and 1% streptomycin (all from Sigma-Aldrich) at 37 °C with atmosphere containing 5% CO_2_. The tested compounds dissolved at DMSO were added to cells suspended at complete cultivation medium, and the relative cell viability (the ratio between cells treated with compounds and cells treated with DMSO only) was measured by a CCK-8 kit (Sigma-Aldrich) after 24 h, as we described previously [99].

## 4. Conclusions

A series of benzyl [2-(arylsulfamoyl)-1-substituted-ethyl]carbamates was designed, prepared by multi-step synthetic procedure, and characterized. All the target compounds were investigated as AChE and BChE inhibitors. While AChE inhibition was insignificant, 11 compounds showed strong preferential inhibition of BChE, and 9 of them were more active than rivastigmine. Benzyl {(2*S*)-1-[(2-methoxybenzyl) sulfamoyl]-4-methylpentan-2-yl}carbamate (**5k**), benzyl {(2*S*)-1-[(4-chlorobenzyl)sulfamoyl]- 4-methylpentan-2-yl}carbamate (**5j**), and benzyl [(2S)-1-(benzylsulfamoyl)-4- methylpentan-2-yl]carbamate (**5c**) showed the highest BChE inhibition (IC_50_ = 4.33, 6.57, and 8.52 µM, respectively), indicating that derivatives 5c and **5j** had approximately 5-fold higher inhibitory activity against BChE than rivastigmine, and **5k** was even 9-fold more effective than rivastigmine. The selectivity index of **5c** and **5j** was 10 and that of **5k** was 34. These results show that the compounds reported here bind to the same active site of the molecular targets as rivastigmine. Our theoretical results are in total agreement with the experimental data, being an additional support for such results. Furthermore, our study using QTAIM calculations gives detailed information on the molecular interactions that stabilize the molecular complexes of the most active compounds in this series. Moreover, the tested compounds showed low cytotoxic effect and, thus, could be worth of further evaluation. In addition, all predicted ADME properties underline the importance of subsequent in vivo studies of these small molecules. The molecular modeling study was performed using combined techniques. Active ligands adopt an extended conformation leading to the interaction of the carbamate group with the active site Ser. Conversely, less active compounds adopt a V-shape conformation, which causes interactions of these ligands with different regions of the enzyme active site. The selectivity displayed by these compounds towards BChE relies on the larger accesible area of this enzyme compared to AChE. Our theoretical results support the experimental data. Furthermore, our study using QTAIM calculations gives detailed information on the molecular interactions that stabilize the molecular complexes of the most active compounds in this series.

## Figures and Tables

**Table 1:** Structures of discussed sulfonamides **5a–n** and their in vitro AChE and BChE inhibition (IC_50_ (μM)) compared with that of standard rivastigmine (RIV). Cholinesterase inhibition is expressed as the mean ± SD (*n* = 3 experiments).

Comp.	Structure	IC_50_ (µM)		SI *
		AChE	BChE	
**5a**	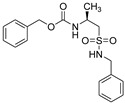	90.56 ± 5.70	100.25 ± 4.26	0.90
**5b**	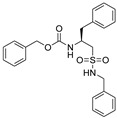	76.95 ± 1.52	86.12 ± 1.91	0.89
**5c**	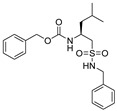	91.81 ± 4.89	**8.52 ± 0.32**	**10.78**
**5d**	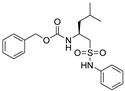	55.64 ± 1.38	16.54 ± 0.10	3.36
**5e**	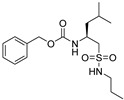	108.46 ± 2.39	58.94 ± 1.88	1.84
**5f**	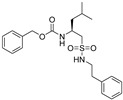	114.73 ± 6.22	15.29 ± 0.38	**7.50**
**5g**	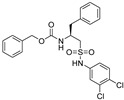	78.83 ± 0.63	12.61 ± 0.31	6.25
**5h**	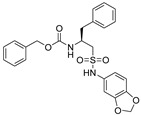	61.39 ± 0.47	11.54 ± 0.28	5.32
**5i**	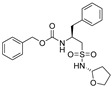	61.65 ± 1.47	112.21 ± 3.41	0.55
**5j**	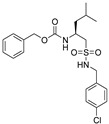	67.47 ± 0.37	**6.57 ± 0.12**	**10.27**
**5k**	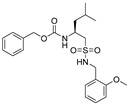	150.29 ± 3.82	**4.33 ± 0.07**	**34.71**
**5l**	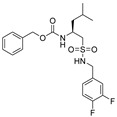	92.91 ± 2.47	55.64 ± 1.38	1.67
**5m**	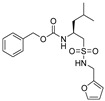	131.85 ± 0.42	19.99 ± 0.17	5.70
**5n**	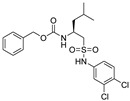	95.14 ± 2.77	13.51 ± 0.51	**7.04**
**RIV**	–	56.10 ± 1.41	38.40 ± 1.97	1.46

* SI (selectivity index) = IC_50_ (AChE)/IC_50_ (BChE). AChE and BChE inhibition are expressed as the mean ± SD (*n* = three independent experiments). The best results of IC_50_ and SI are indicated in bold.

**Table 2:** Values of parameters predicted using ACD/Percepta ver. 2012 and characterizing the properties of selected most pronounced BChE inhibitors in comparison with rivastigmine and galantamine in relation to Lipinski’s Rule of Five (Ro5) and some other selected criteria.

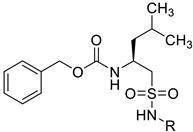													
Comp.	R	MW	logP	HBD	HBA	RB	TPSA	HJS (cm/s)	*k*_a_ (min^−1^)	log*K*_a_^HSA^	%PPB	logPS	logBB
**5c**	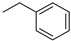	404.52	3.69	2	6	11	92.88	7.22 × 10^−4^	0.050	5.18	90.52	−1.3	0.49
**5f**	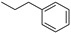	418.55	4.04	2	6	12	92.88	7.13 × 10^−4^	0.049	5.22	91.70	−1.3	0.63
**5j**	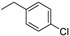	438.97	4.68	2	6	11	92.88	7.15 × 10^−4^	0.0449	5.34	94.64	−1.2	0.74
**5k**	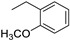	434.55	3.95	2	7	12	102.11	7.09 × 10^−4^	0.049	5.20	89.60	−1.3	0.68
**5n**	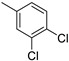	459.39	5.17	2	6	10	92.88	7.17 × 10^−4^	0.049	5.68	96.90	−1.8	0.42
**rivastigmine**		250.34	2.29	0	4	5	32.78	6.89 × 10^−4^	0.047	3.57	33.55	−1.9	0.51
**galantamine**		287.35	1.55	1	4	1	41.93	4.37 × 10^−4^	0.030	3.38	19.99	−2.2	0.28
**Ro5**		**<500**	**<5**	**<5**	**<10**	–	–	–	–	–	–	–	–

Molecular weight (MW), lipophilicity (logP), number of H-bond donors (HBD), number of H-bond acceptors (HBA), number of rotatable bonds (RB), topological polar surface area (TPSA), human jejunum scale at pH = 6.5 (HJS), absorption rate from jejunum (*k*_a_), protein binding (%PPB), rate of brain panetration (logPS), extent of brain penetration (logBB).

## Data Availability

Data are contained within the article.

## Supplementary Materials

The following is available online at https://www.mdpi.com/article/10.3390/ijms22179447/s1.
