# Supplementary material for: Novel Sulfonamide-Based Carbamates as Selective Inhibitors of BChE
Source: Int J Mol Sci. 2021 Aug 31;22(17):9447. doi: 10.3390/ijms22179447 (PMC8430704; doi:10.3390/ijms22179447)
Supplement: Supplementary file 1 [file ijms-22-09447-s001.zip › ijms-1371097-supplementary.pdf]

Supporting Materials

Novel sulfonamide-based carbamates as selective inhibitors of BChE

Pratibha Magara[1], Oscar Parravicini[2], Šárka Štěpánková[3], Katarina Svrčková[3], Adriana D. Garro[2], Izabela Jendrzejewska[4], Karel Pauk[1], Jan Hošek[5], Josef Jampilek[6,7], Ricardo D. Enriz[2]* and Aleš Imramovský[1]*

1. Institute of Organic Chemistry and Technology, Faculty of Chemical Technology, University of Pardubice, Studentska 573, 532 10 Pardubice, Czech Republic; partibha.magar@gmail.com (P.M.); ales.imramovsky@upce.cz (A.I.)
2. Facultad de Química, Bioquímica y Farmacia, Universidad Nacional de San Luis; Instituto Multidisciplinario de Investigaciones Biológicas (IMIBIO-SL).Chacabuco 915, 5700 San Luis, Argentina; e-mail: denriz@unsl.edu.ar
3. Department of Biological and Biochemical Sciences, Faculty of Chemical Technology, University of Pardubice, Studentska 573, 532 10 Pardubice, Czech Republic; sarka.stepankova@upce.cz (S.S.); katarina.svrckova@upce.cz (K.S.)
4. Institute of Chemistry, University of Silesia, Szkolna 9, 40007 Katowice, Poland; izabela.jendrzejewska@us.edu.pl
5. Department of Pharmacology and Toxicology, Veterinary Research Institute, Hudcova 296/70, 621 00 Brno, Czech Republic; hosek.jan@vri.cz
6. Department of Analytical Chemistry, Faculty of Natural Sciences, Comenius University, Ilkovicova 6, 842 15 Bratislava, Slovakia; josef.jampilek@gmail.com
7. Institute of Neuroimmunology, Slovak Academy of Sciences, Dubravska Cesta 9, 845 10 Bratislava, Slovakia

* Correspondence: denriz@unsl.edu.ar (R.E.), ales.imramovsky@upce.cz (A.I.)

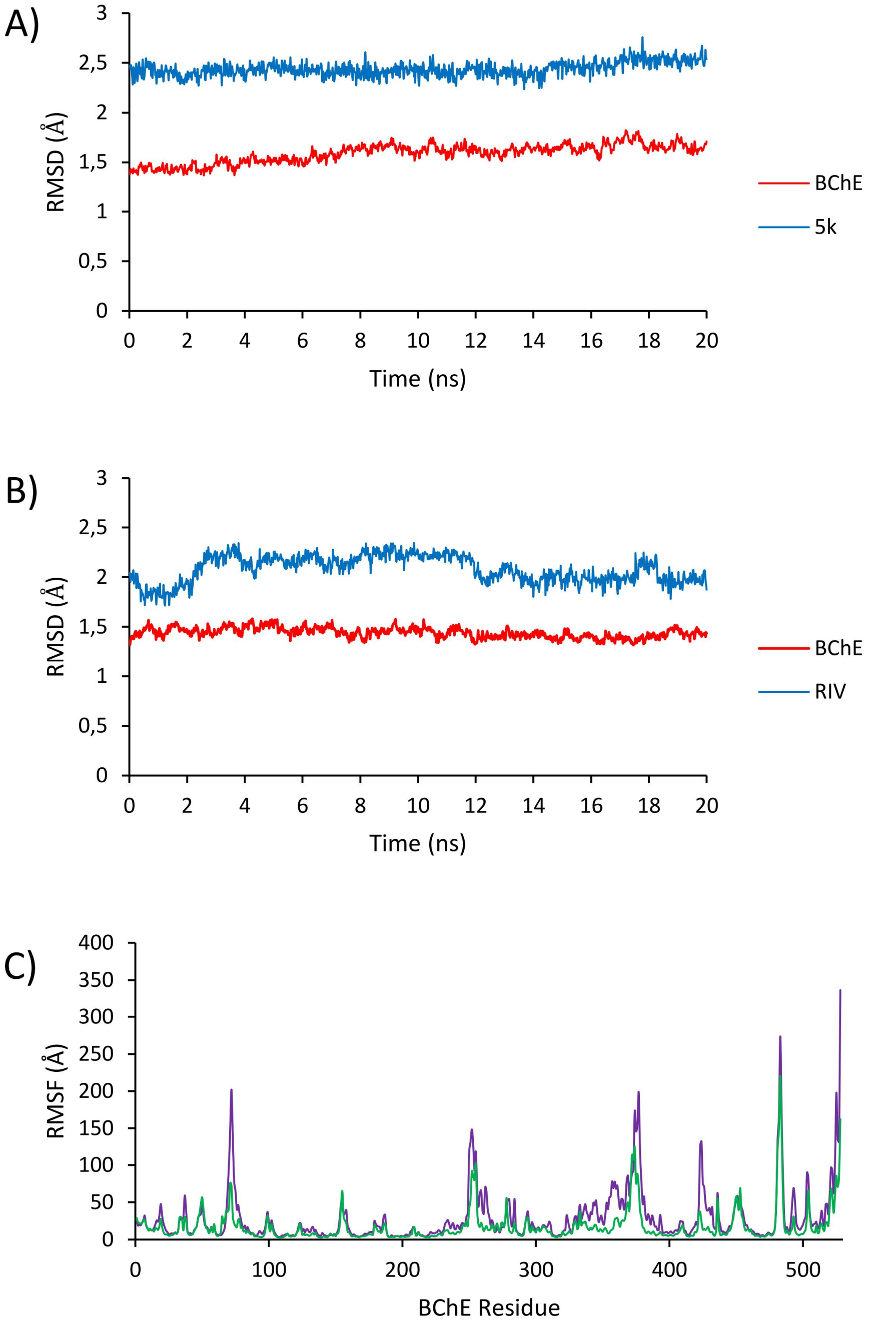

Figure S1 Root-mean-square deviations (RMSD) of (A) BChE-5k and (B) BChE-RIV complexes. (C) Comparative root-mean-square fluctuations (RSMF) of the Cα atoms of BChE when complexed with 5k (violet) and RIV (green).

Experimental part

All reagents and solvents were purchased from commercial sources (TCI Europe, Sigma-Aldrich, Acros organics, fluorochem, Merck, Lach-Ner). Commercial grade reagents were used without further purification. Reactions were monitored by thin layer chromatography plates coated with 0.2 mm silica gel 60 F_{254} (Merck). TLC plates were visualized by UV irradiation (254 nm) or in a 5% solution of phosphomolybdic acid in ethanol. All melting points were determined on a Melting Point B-540 apparatus (Büchi, Switzerland) and are uncorrected. IR spectra were recorded on a Nicolet 6700 FT-IR spectrometer (Thermo Fisher Scientific, Waltham, MA, USA) over the range of 400–4000 cm^{-1} using the ATR technique. NMR spectra were measured in $CDCl_3$ solutions at ambient temperature on a Bruker Avance™ III 400 spectrometer at frequencies ^1H (400 MHz), ^{13}C (100.26 MHz), and ^{19}F (376.46) or a Bruker Ascend™ 500 spectrometer at frequencies ^1H (500.13 MHz), ^{13}C {^1H} (125.76 MHz), and ^{19}F (470.61 MHz). Chemical shifts, δ, are given in ppm, related to the residual solvent peak $CDCl_3$ 7.27 or to tetramethylsilane (TMS) as an internal standard. The coupling constants (*J*) are reported in [Hz]. Elemental analysis (C, H, N, S) was performed on an automatic microanalyzer Flash 2000 organic elemental analyzer. Mass spectrometry with high resolution was performed by the dried droplet method using a MALDI mass spectrometer LTQ Orbitrap XL (Thermo Fisher Scientific) equipped with a nitrogen UV laser (337 nm, 60 Hz). Spectra were measured in the positive ion mode and in regular mass extent with resolution 100,000 at m/z = 400. 2,5-Dihydroxybenzoic acid (DBH) was used as the matrix.

Characterization data of prepared compounds:

1.1. Copy of ^{1}H NMR of **2a**

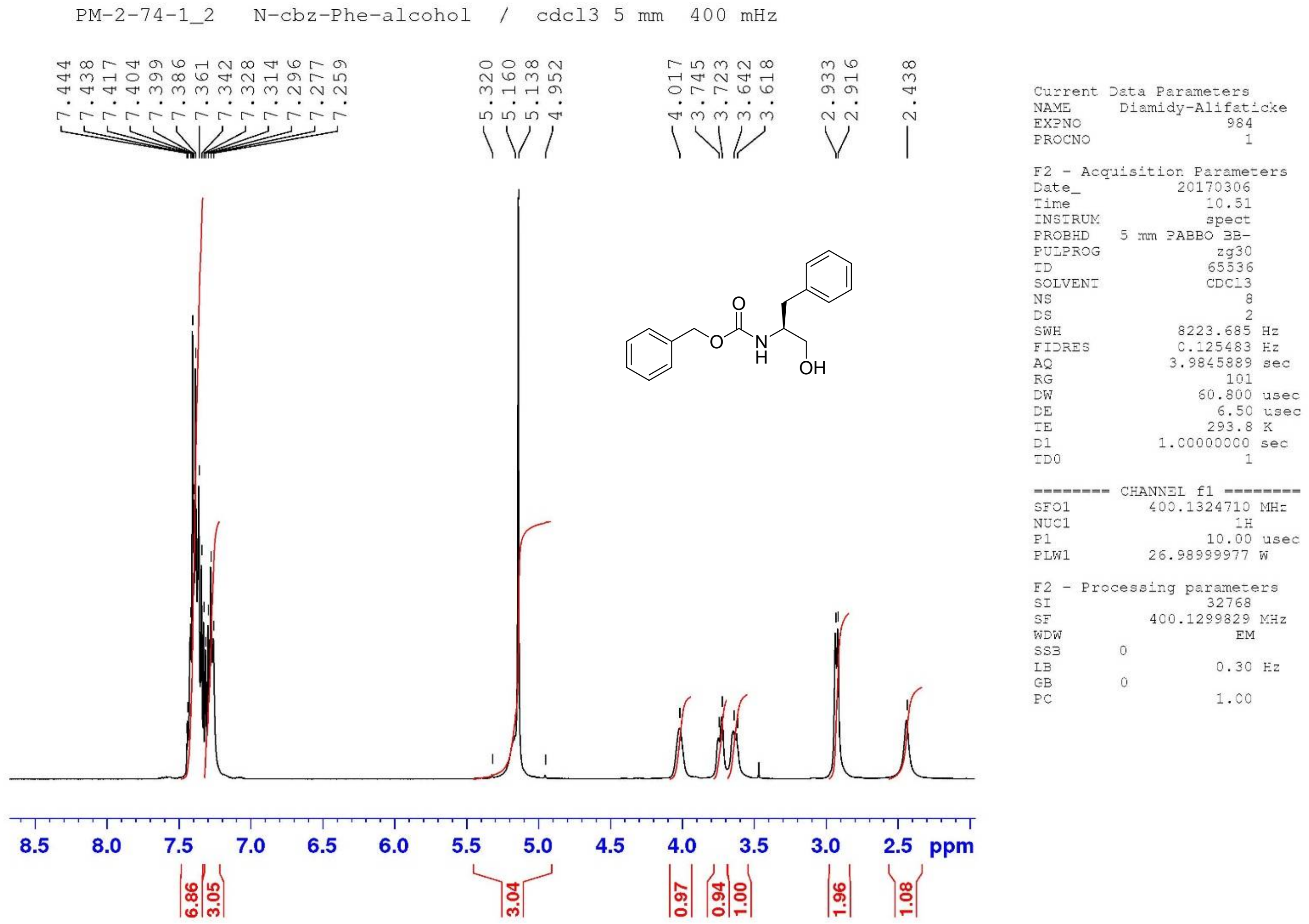

1.2. Copy of ^{13}C NMR of **2a**

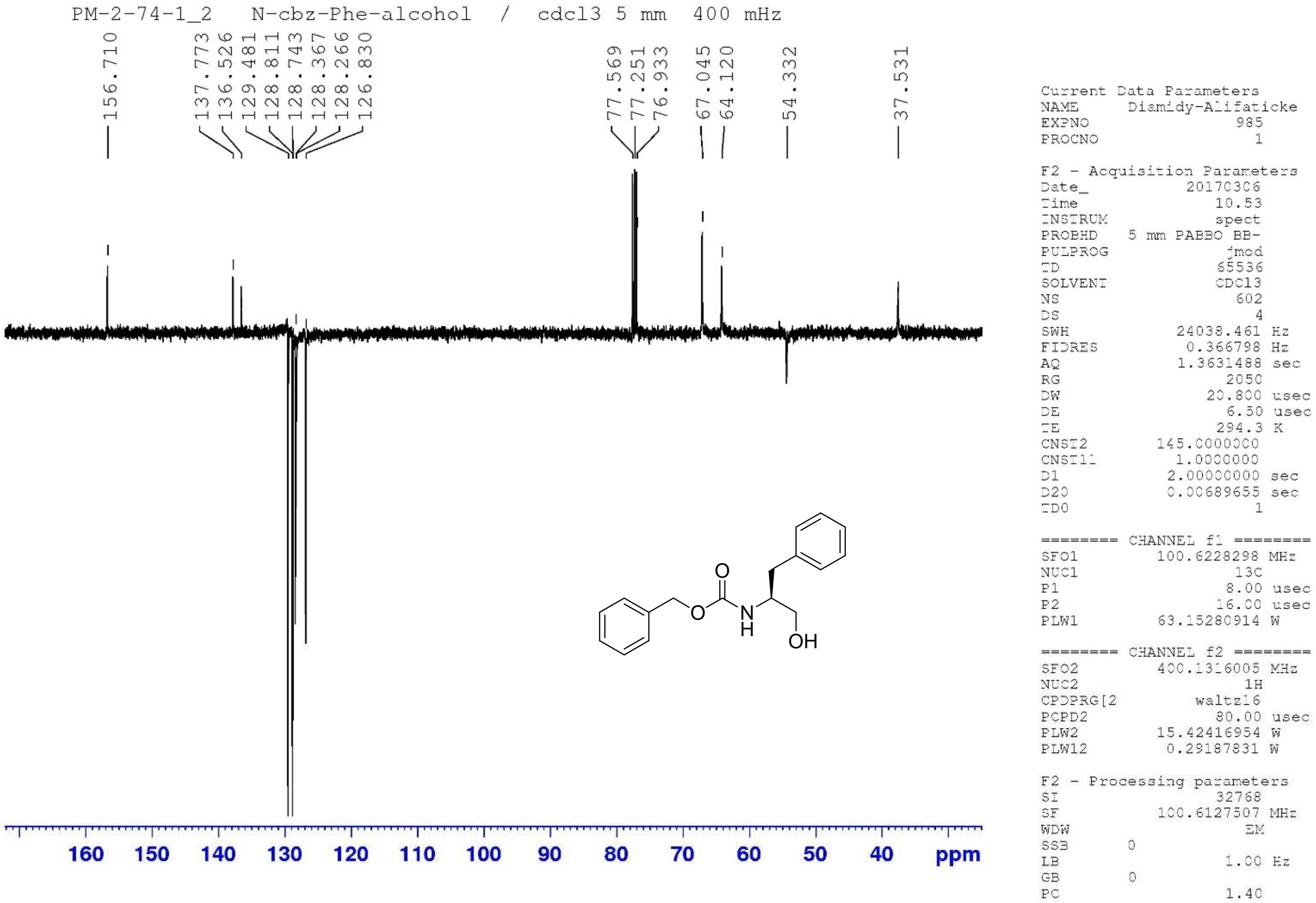

1.3. Copy of ^{1}H NMR of **2b**

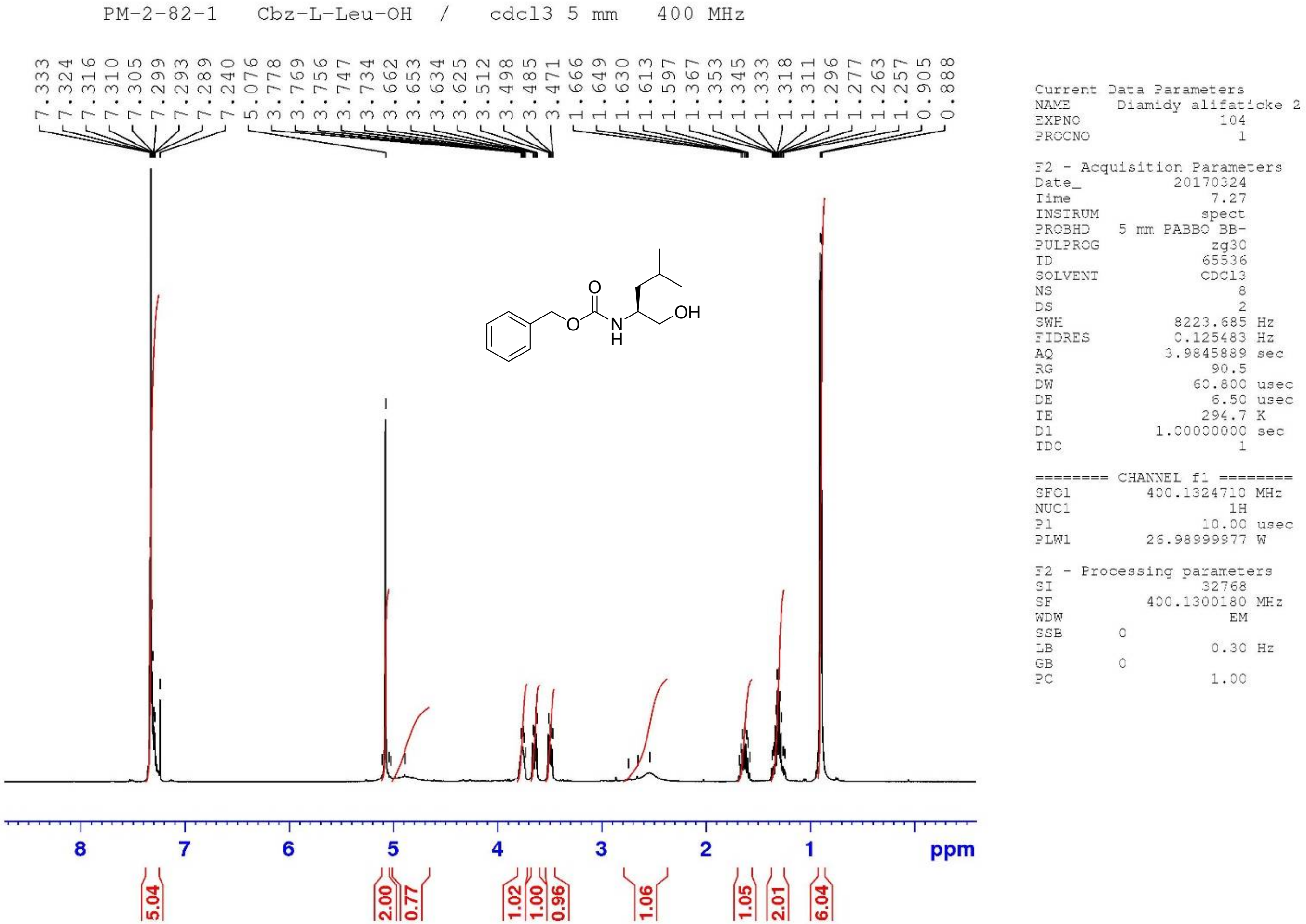

1.4. Copy of ^{13}C NMR of **2b**

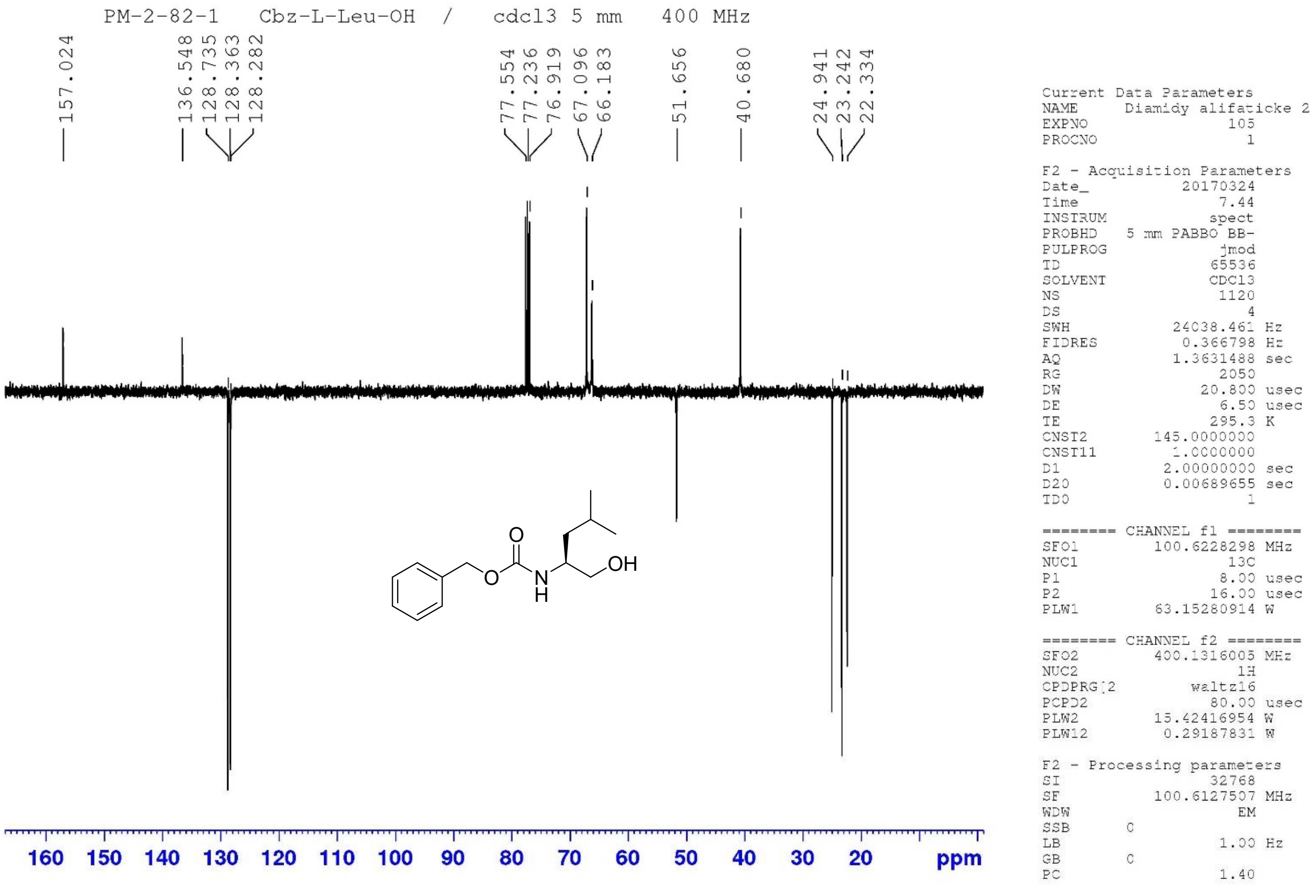

1.5. Copy of ^{1}H NMR of **2c**

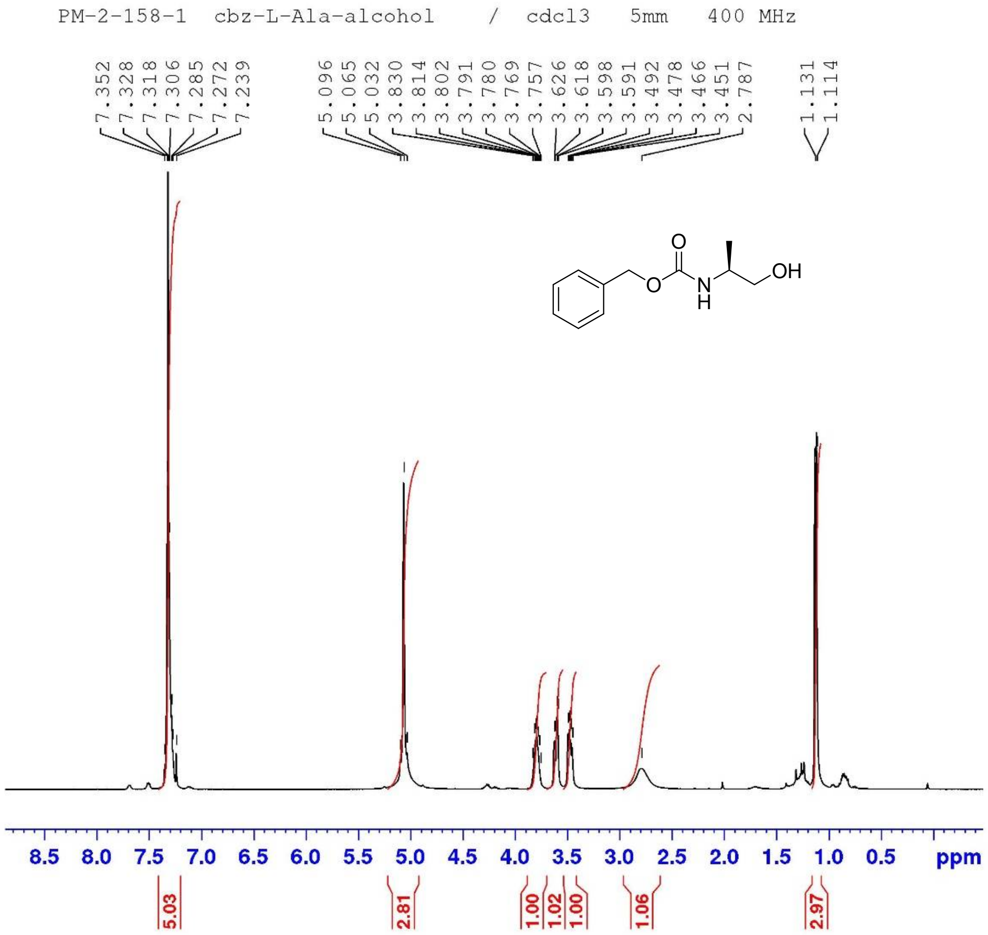


```
Current Data Parameters
NAME     Diamidy alifaticke 2
EXPNO                     925
PROCNO                      1

F2 - Acquisition Parameters
Date_            20201203
Time                10.56
INSTRUM             spect
PROBHD   5 mm PABBO BB-
PULPROG              zg30
TD                  65536
SOLVENT             CDCl3
NS                     16
DS                      2
SWH              8223.685 Hz
FIDRES           0.125483 Hz
AQ              3.9845889 sec
RG                    101
DW                 60.800 usec
DE                   6.50 usec
TE                  294.5 K
D1             1.00000000 sec
TD0                     1

======== CHANNEL f1 ========
SFO1          400.1324710 MHz
NUC1                   1H
P1                  11.00 usec
PLW1          16.15109062 W

F2 - Processing parameters
SI                  32768
SF            400.1300185 MHz
WDW                    EM
SSB      0
LB                   0.30 Hz
GB       0
PC                   1.00
```

1.6. Copy of ^{13}C NMR of **2c**

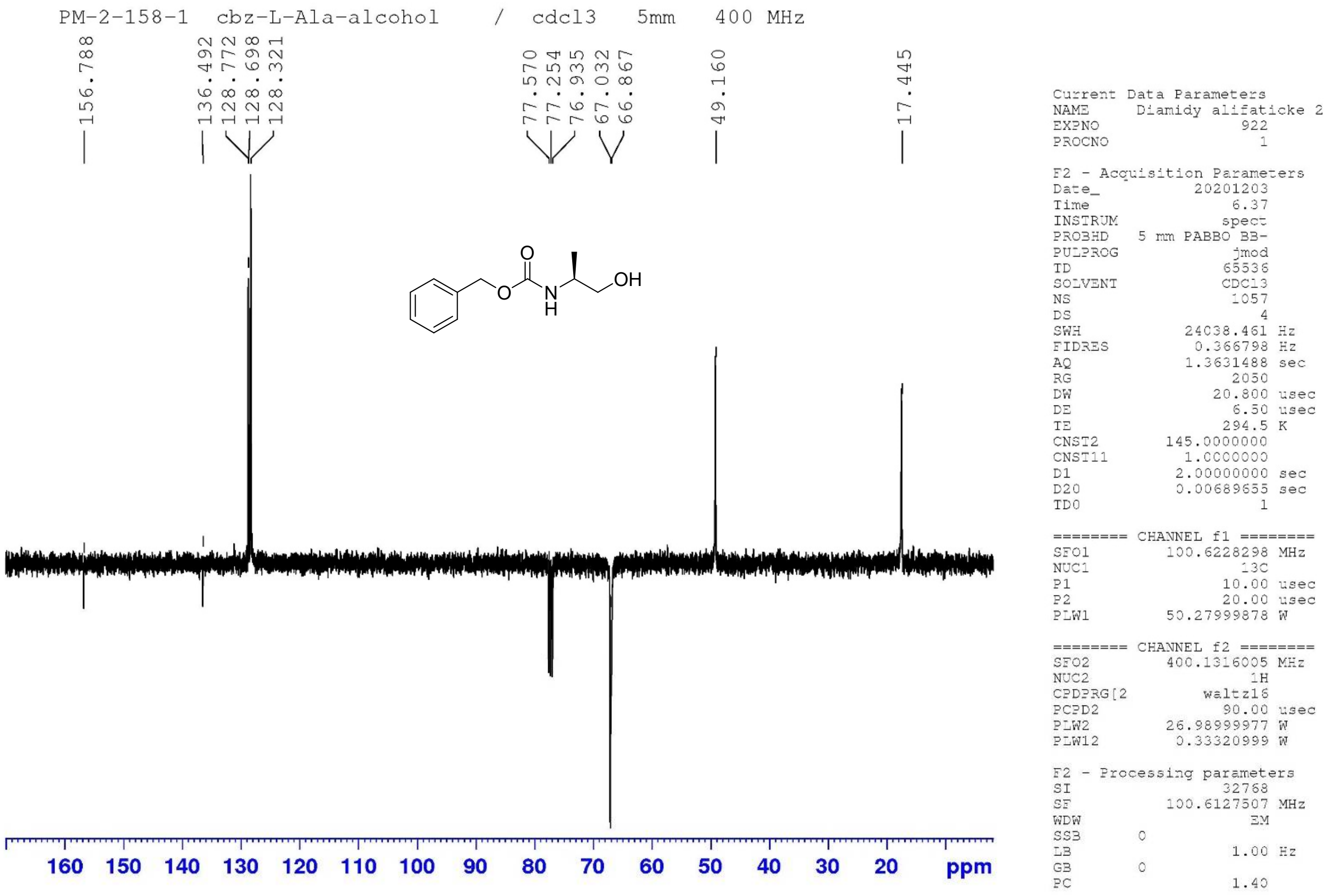

1.7. Copy of ^{1}H NMR of **3a**

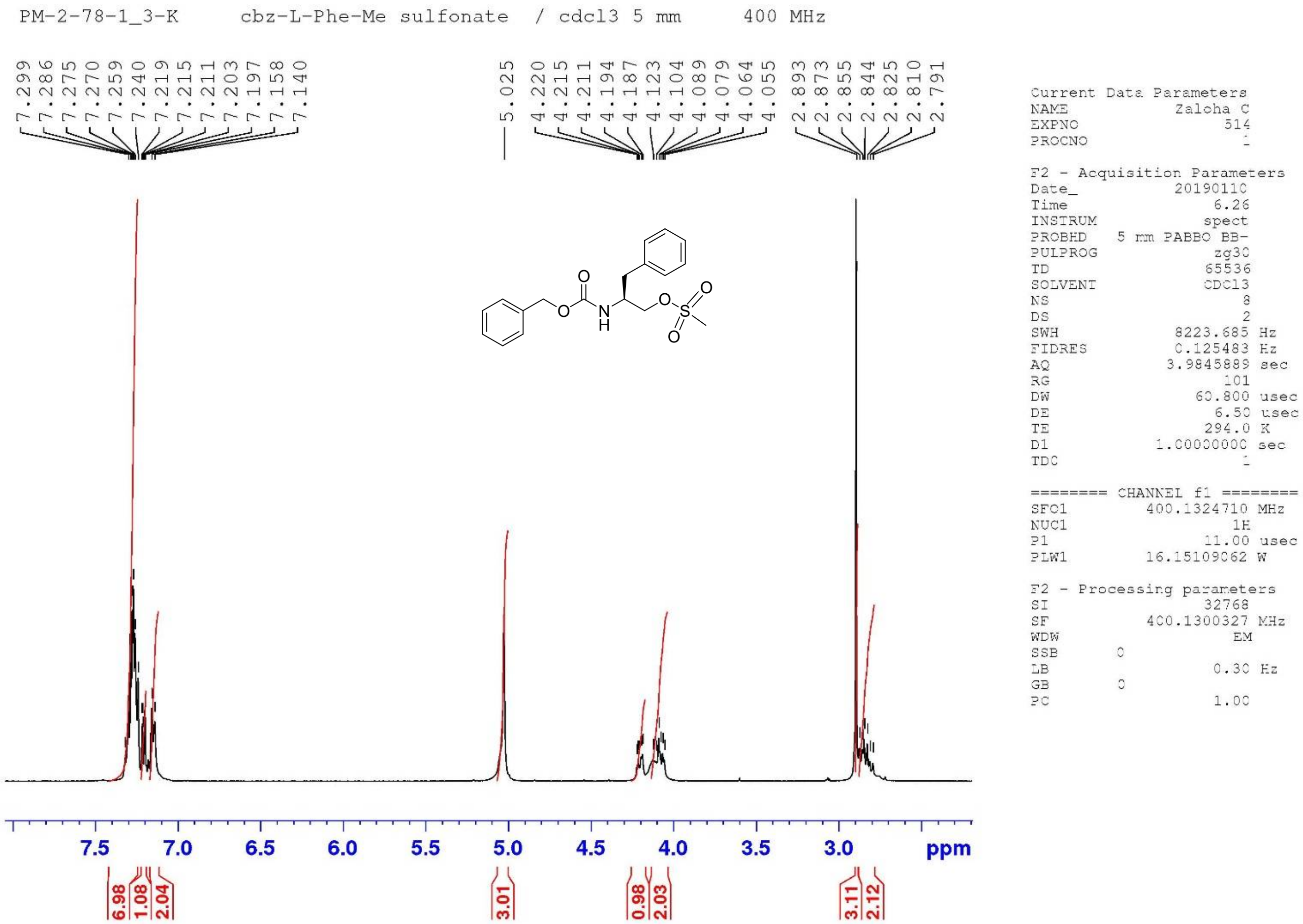

1.8. Copy of ^{13}C NMR of **3a**

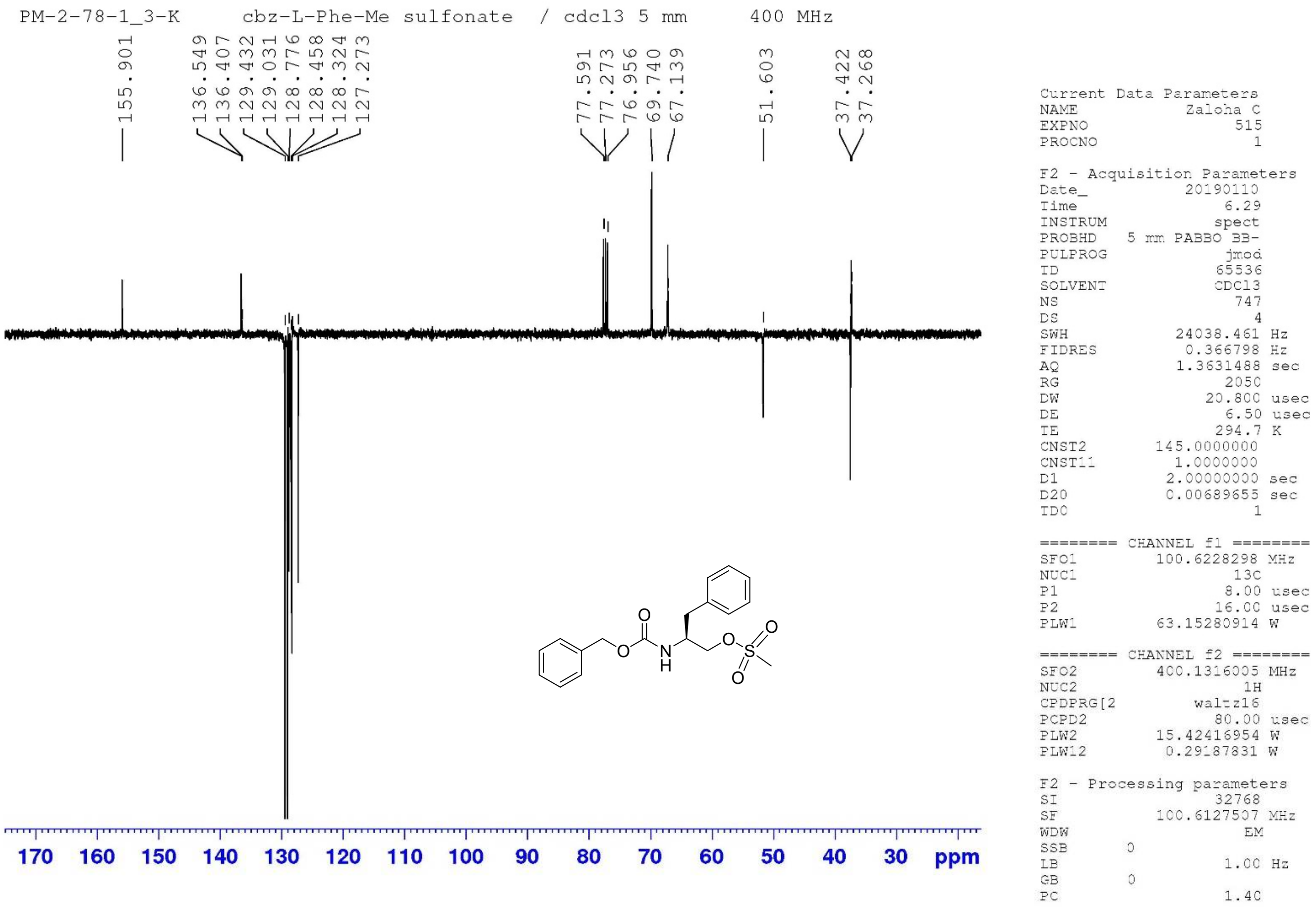

1.9. Copy of ^{1}H NMR of **3b**

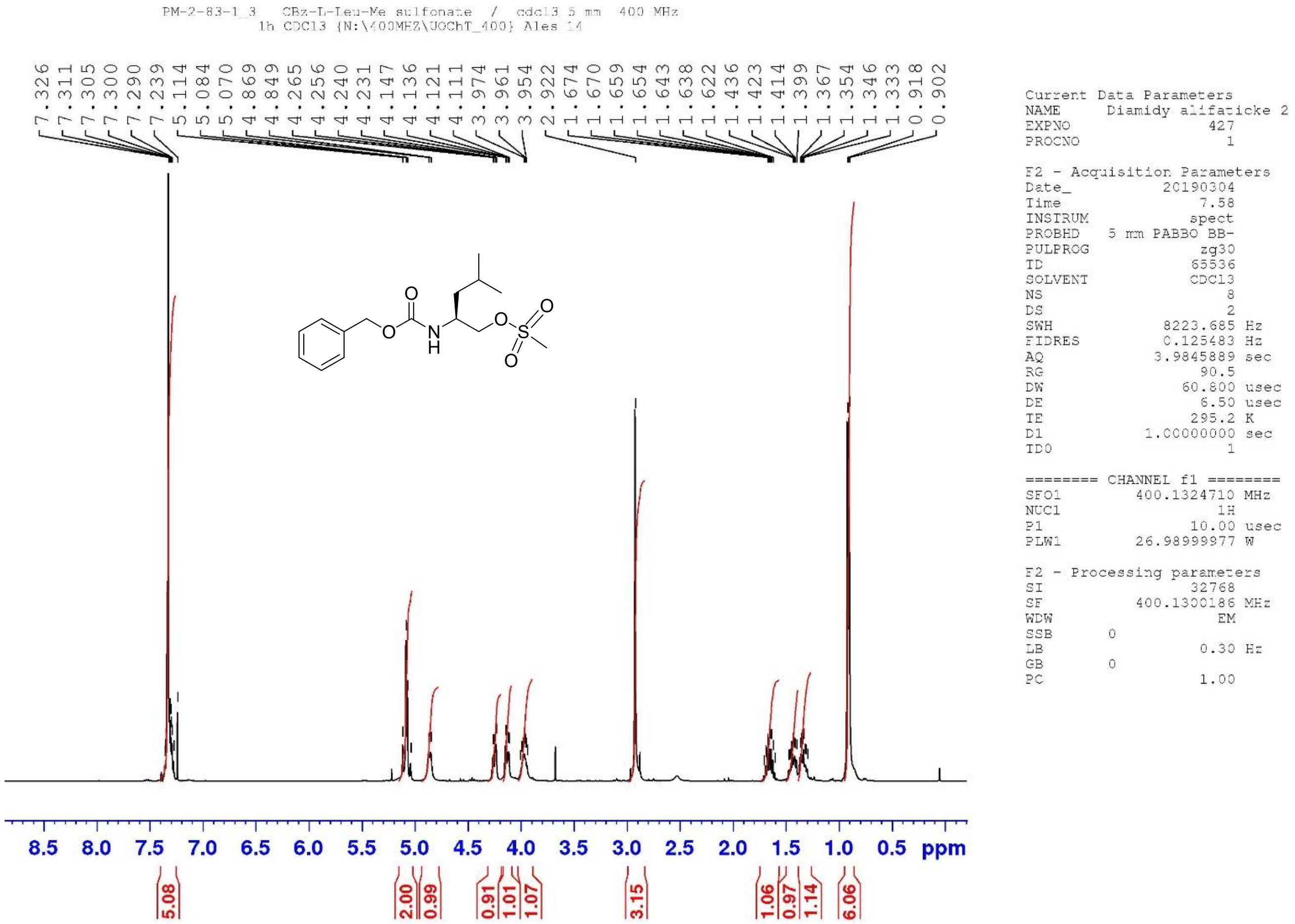

1.10. Copy of ^{13}C NMR of **3b**

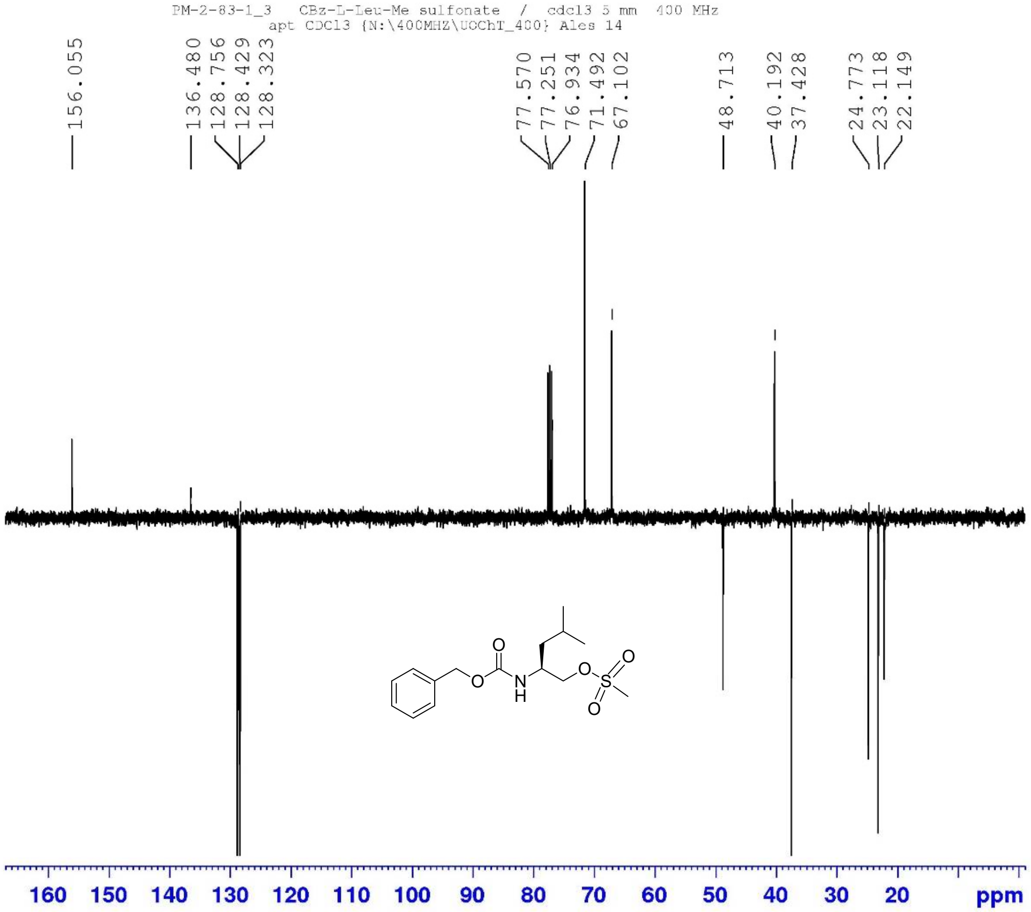


```
Current Data Parameters
NAME       Diamidy alifaticke 2
EXPNO                  428
PROCNO                   1

F2 - Acquisition Parameters
Date_             20190304
Time                  8.29
INSTRUM              spect
PROBHD     5 mm PABBO BB-
PULPROG               jmod
TD                   65536
SOLVENT              CDCl3
NS                     500
DS                       4
SWH              24038.461 Hz
FIDRES            0.366798 Hz
AQ               1.3631488 sec
RG                    2050
DW                  20.800 usec
DE                    6.50 usec
TE                   296.2 K
CNST2          145.0000000
CNST11           1.0000000
D1              2.00000000 sec
D20             0.00689655 sec
TD0                      1

======== CHANNEL f1 ========
SFO1           100.6228298 MHz
NUC1                   13C
P1                   10.00 usec
P2                   20.00 usec
PLW1           50.27999878 W

======== CHANNEL f2 ========
SFO2           400.1316005 MHz
NUC2                    1H
CPDPRG[2           waltz16
PCPD2                90.00 usec
PLW2           26.98999977 W
PLW12           0.33320999 W

F2 - Processing parameters
SI                   32768
SF             100.6127507 MHz
WDW                     EM
SSB        0
LB                    1.00 Hz
GB         0
PC                    1.40
```

1.11. Copy of ^{1}H NMR of **3c**

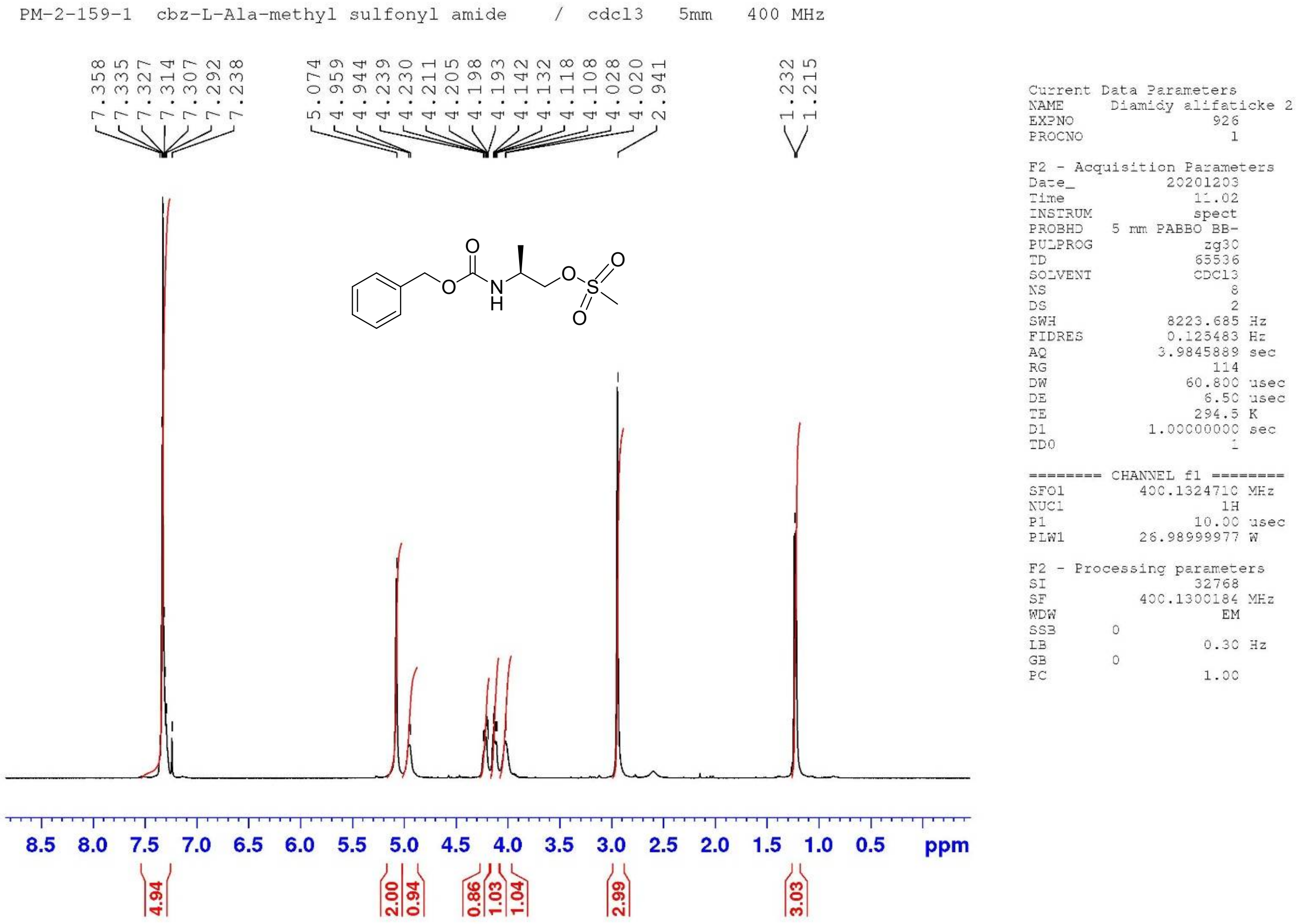

1.12. Copy of ^{13}C NMR of **3c**

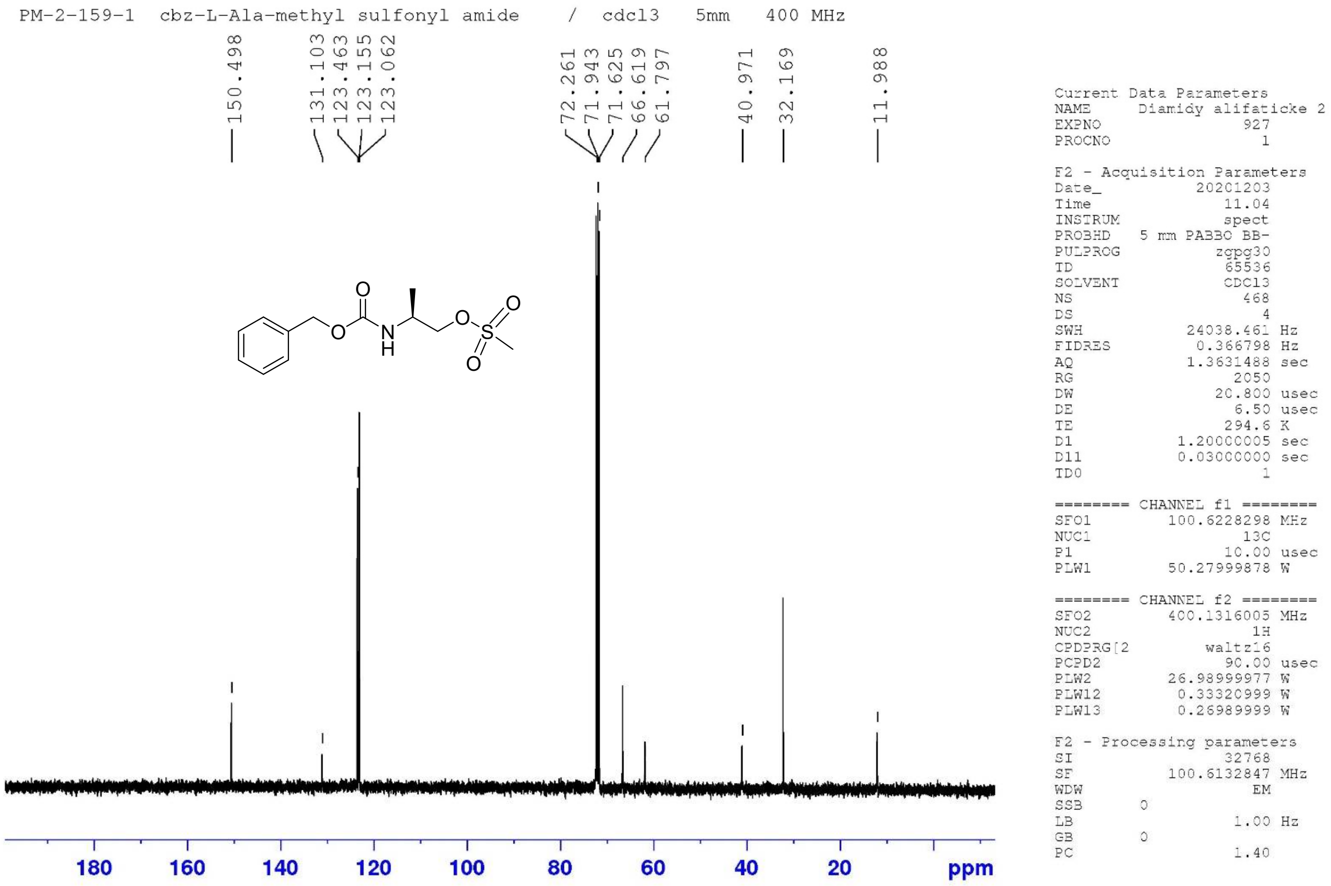

1.13. Copy of ^{1}H NMR of **4a**

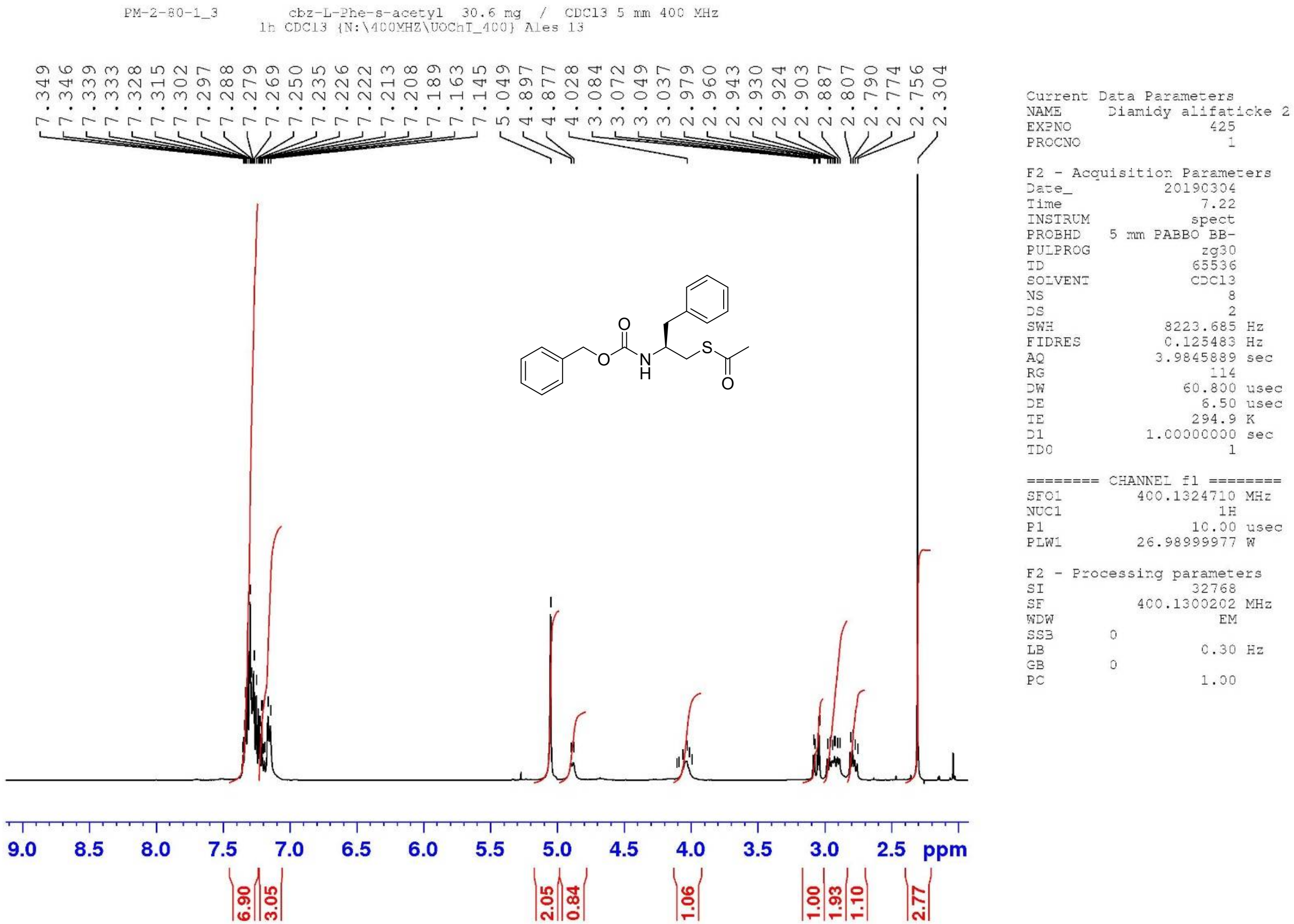

1.14. Copy of ^{13}C NMR of **4a**

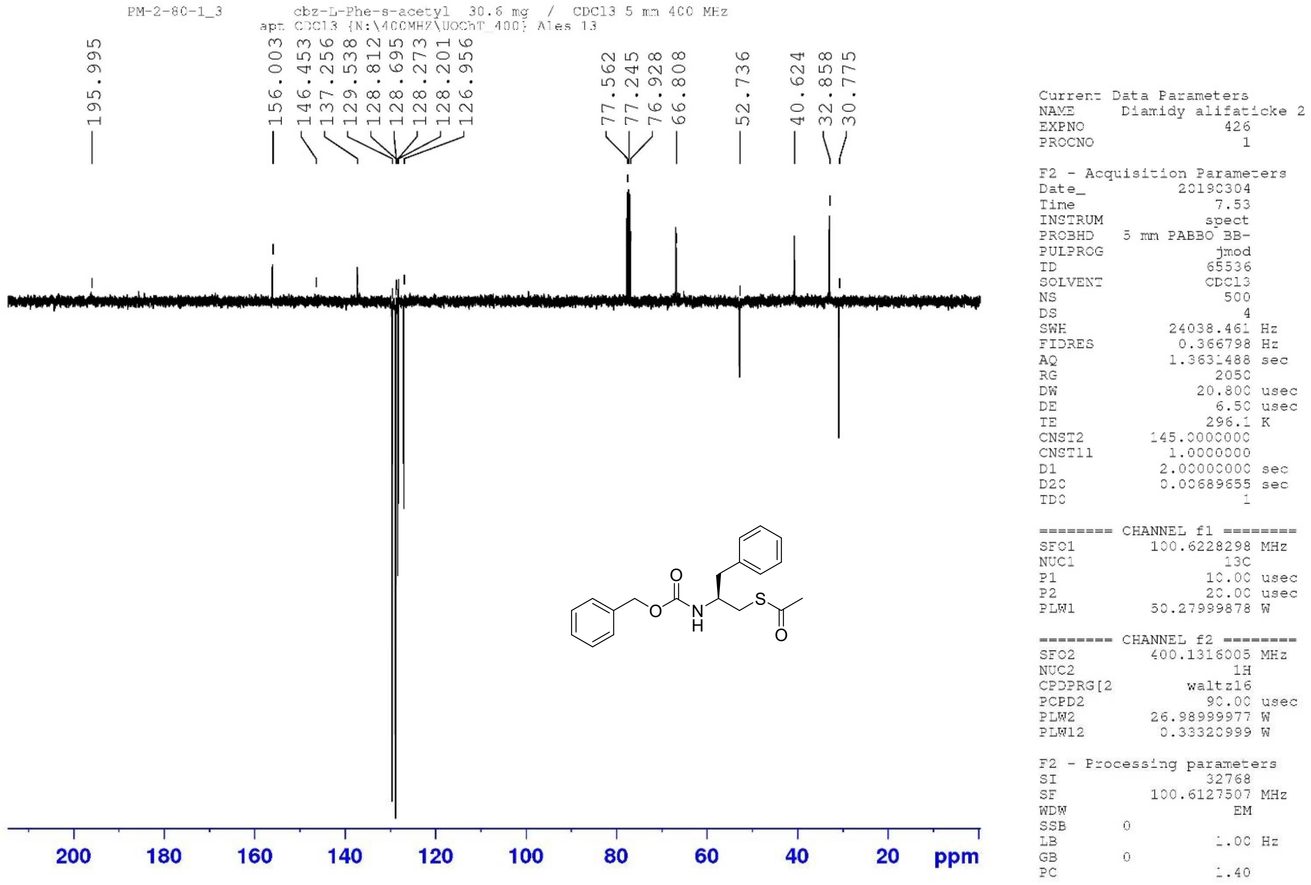

1.15. Copy of 1H NMR of **4b**

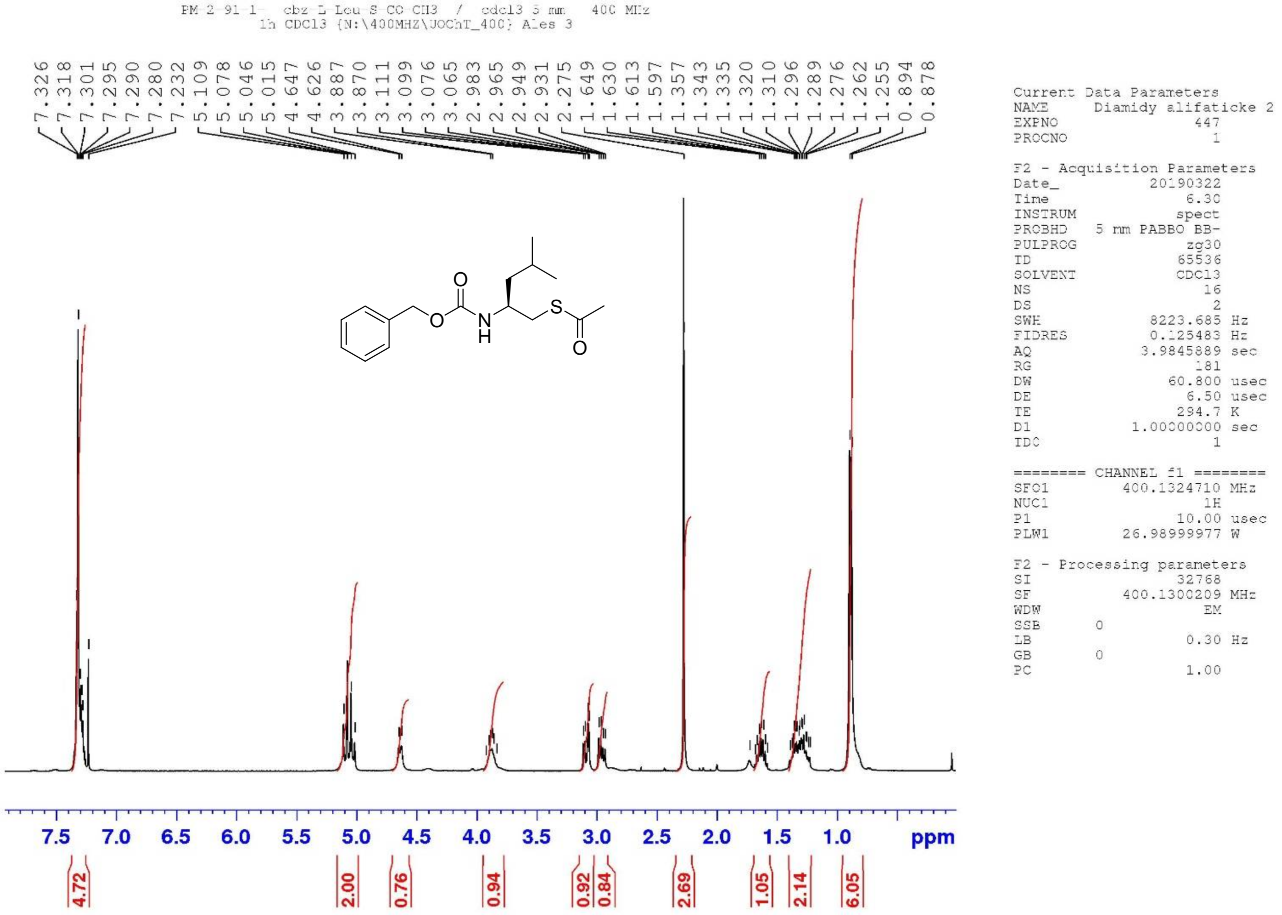

1.16. Copy of ^{13}C NMR of **4b**

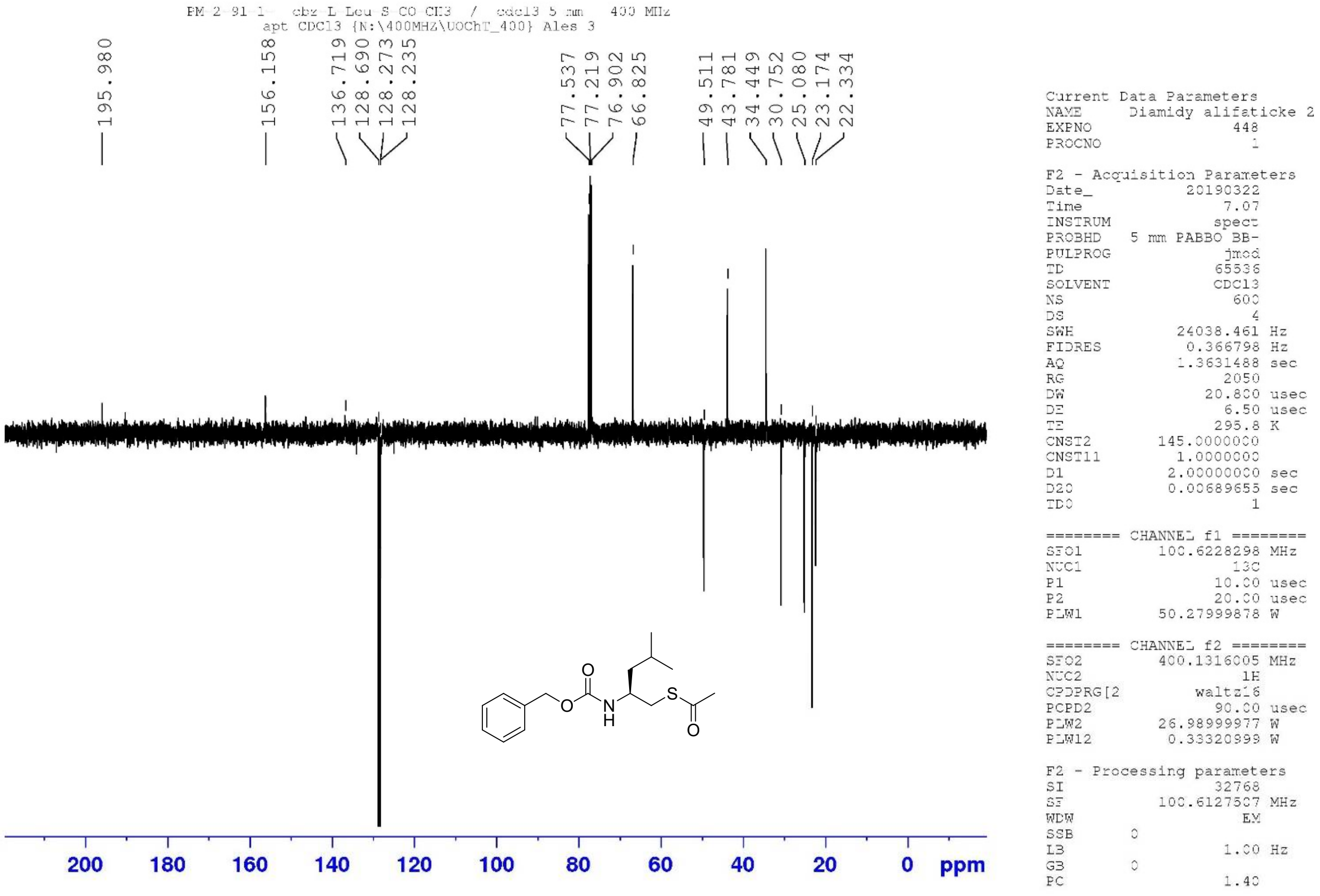

1.17. Copy of ^{1}H NMR of **4c**

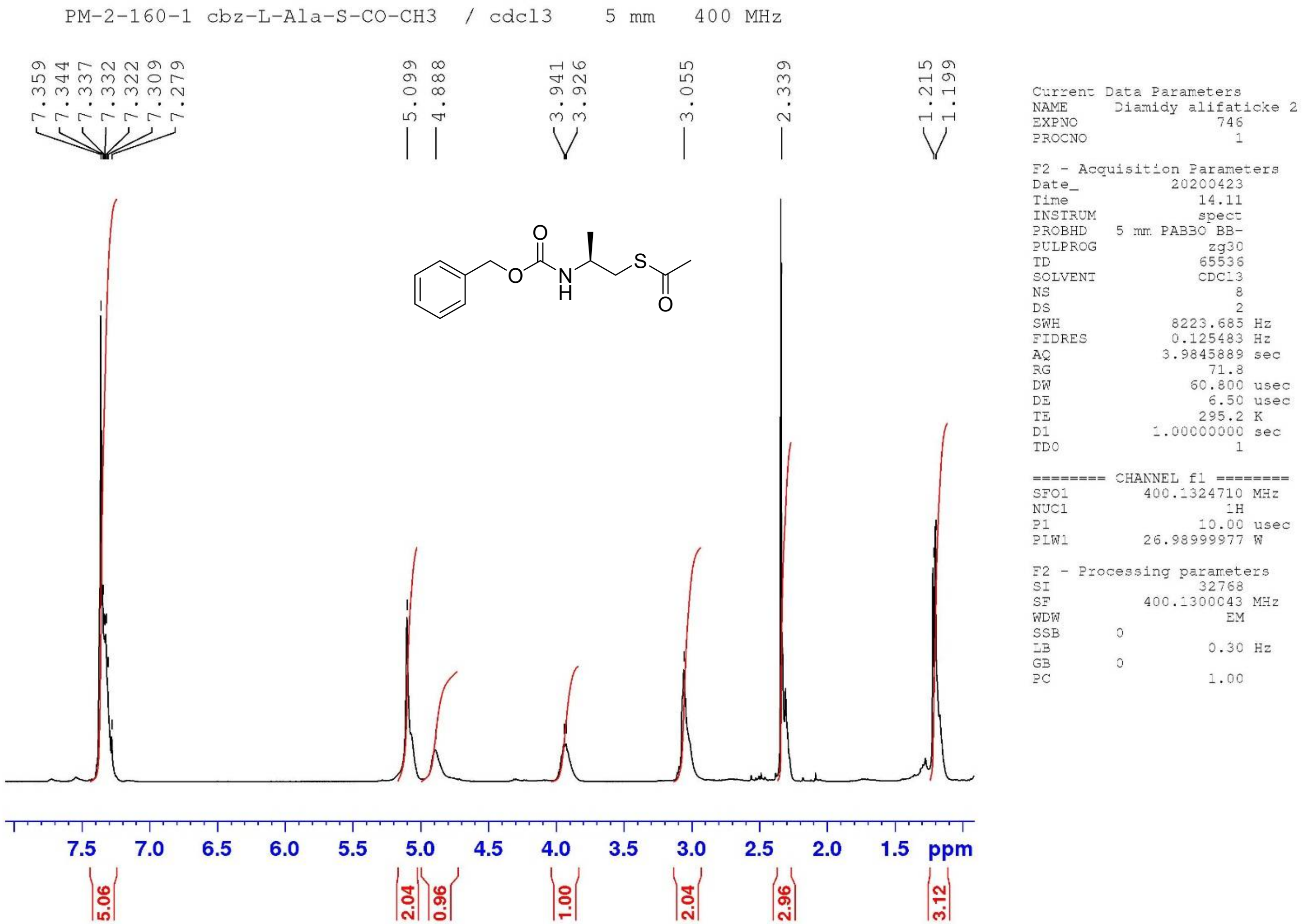

1.18. Copy of ^{13}C NMR of **4c**

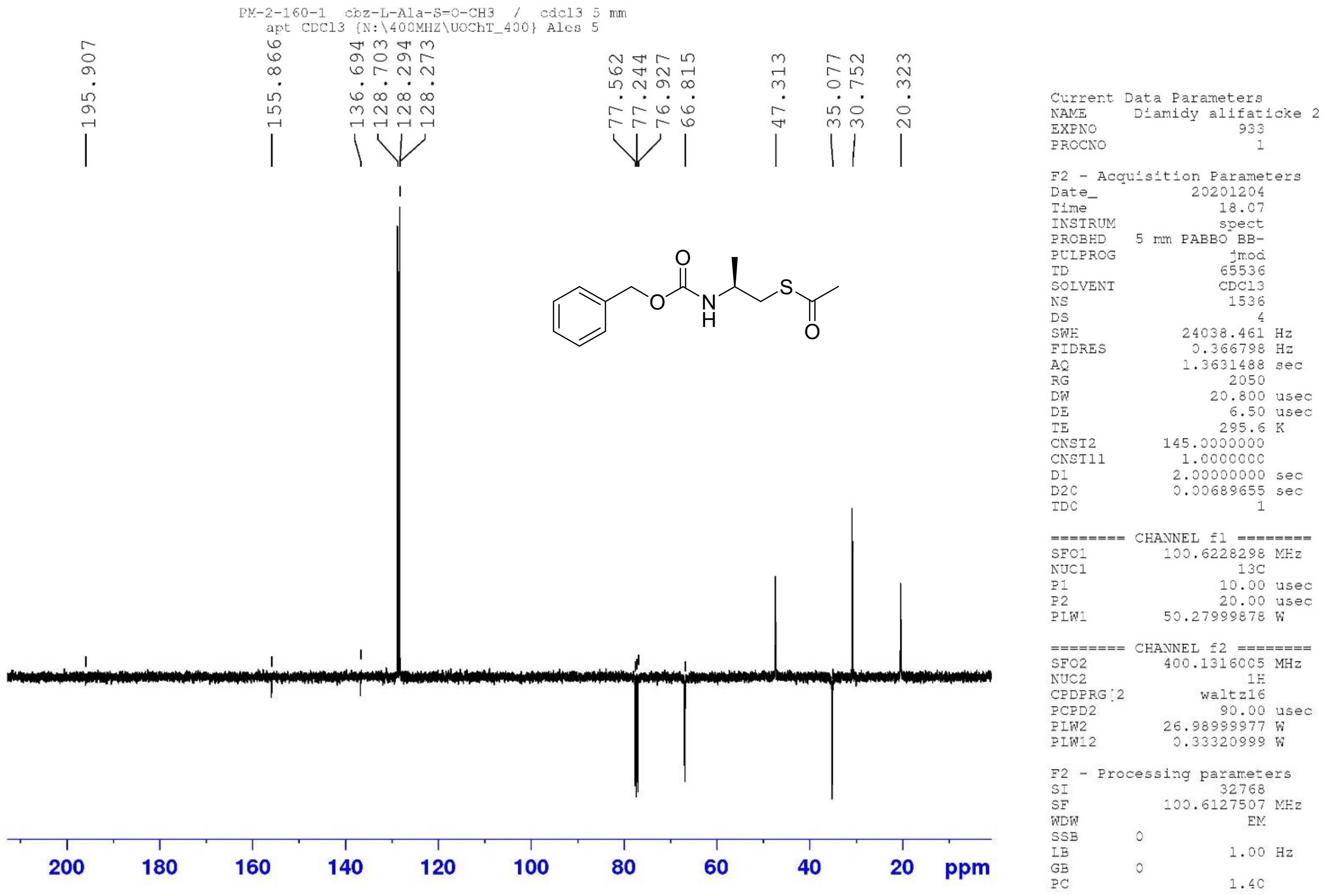

1.19. Copy of ^{1}H NMR of **5a**

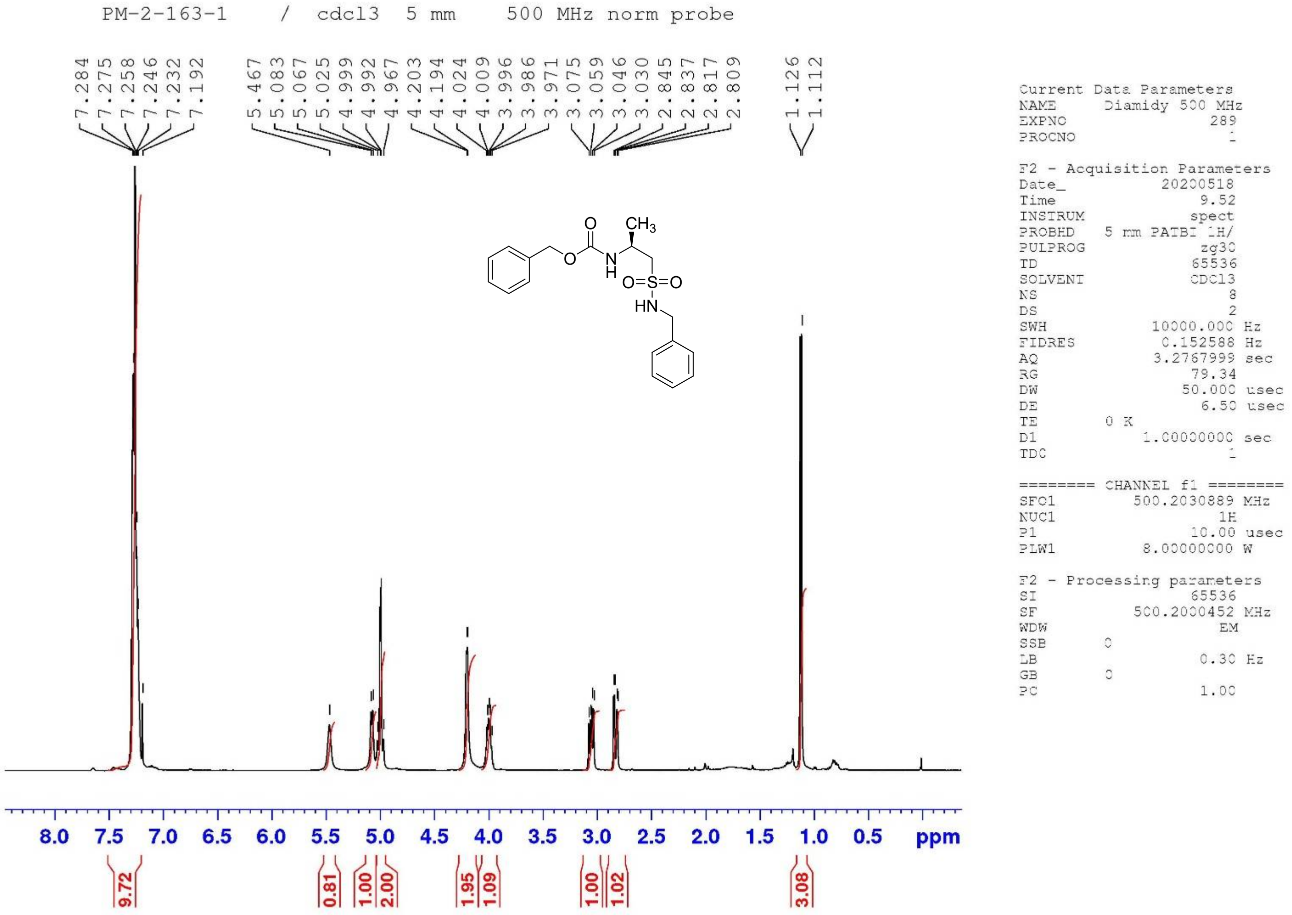

1.20. Copy of ^{13}C NMR of **5a**

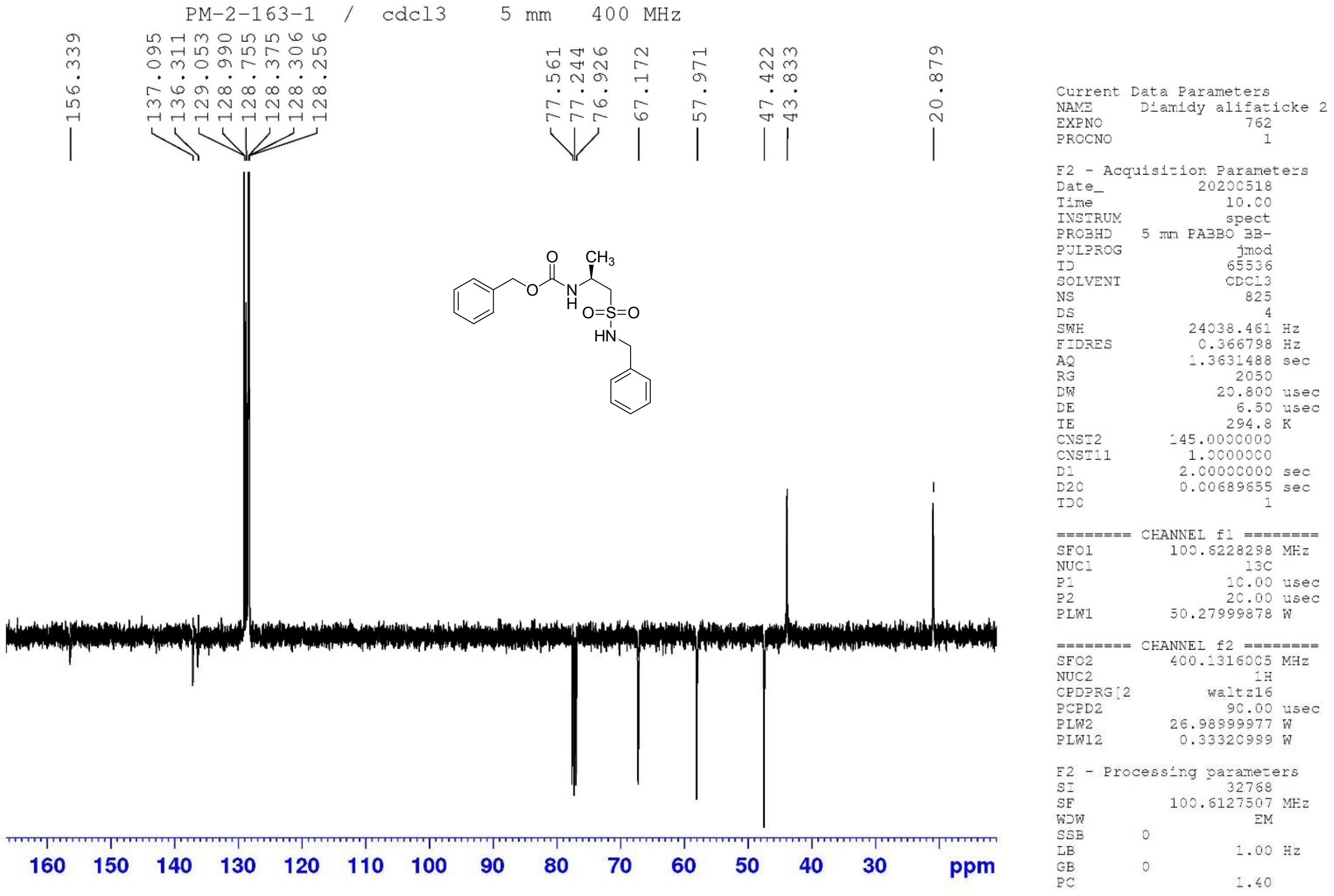

1.21. Copy of ^{1}H NMR of **5b**

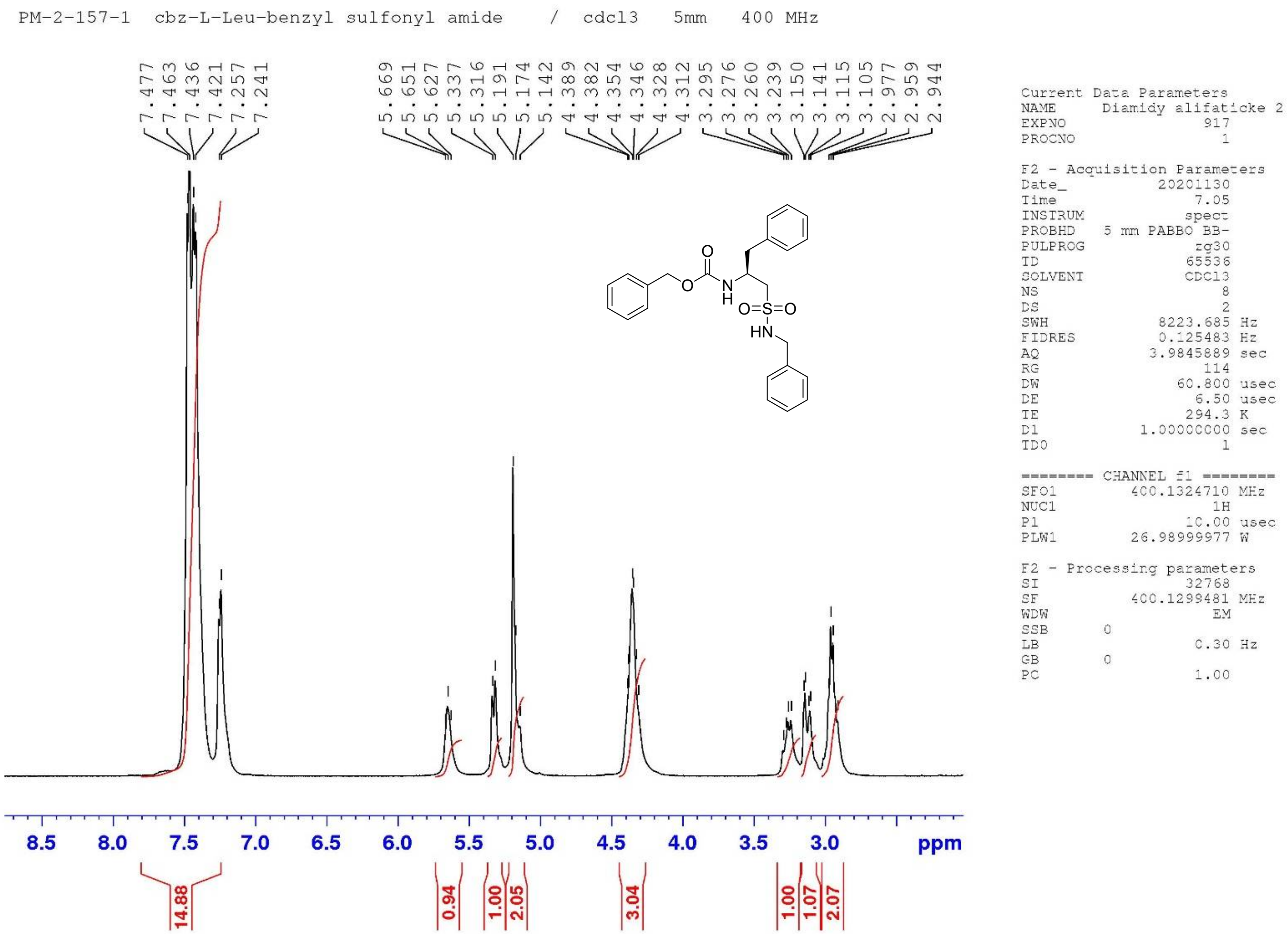

1.22. Copy of ^{13}C NMR of **5b**

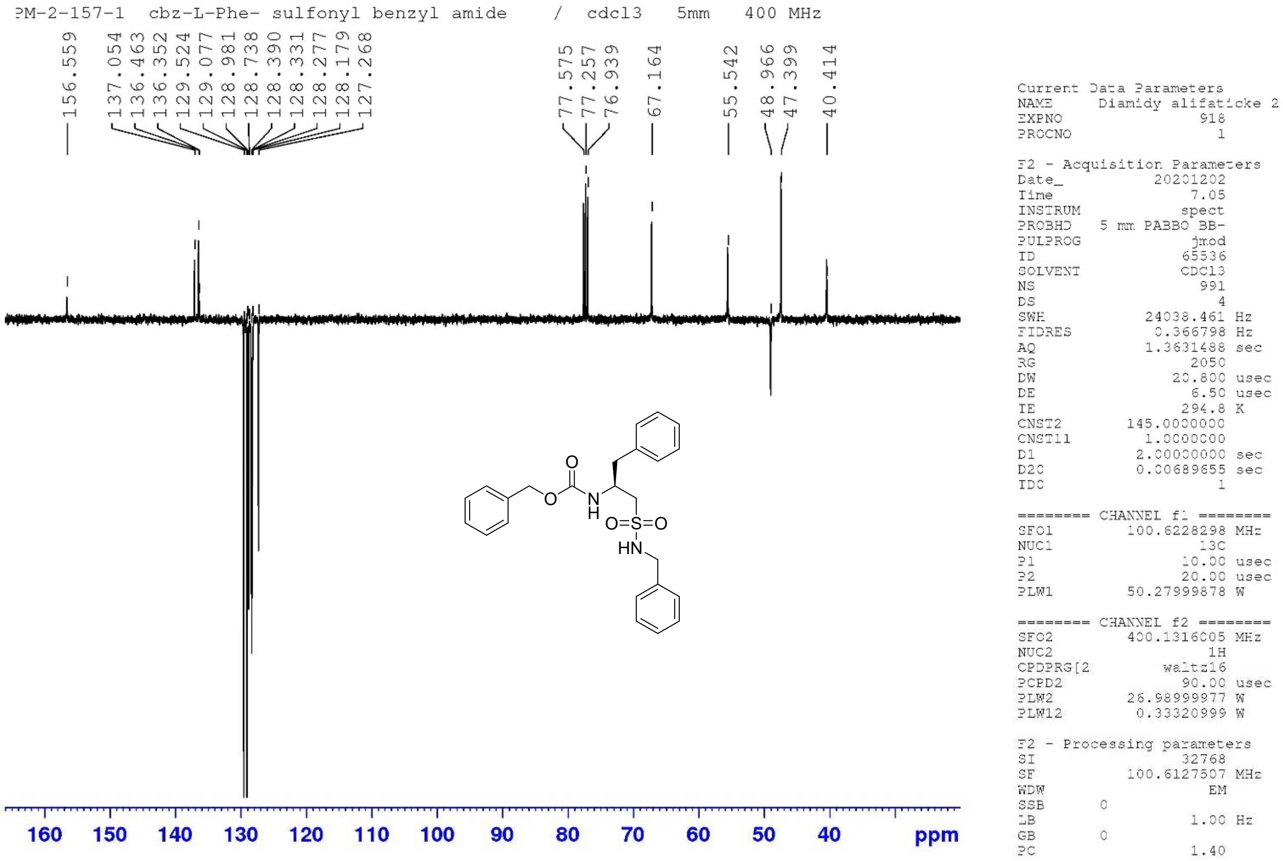

1.23. Copy of ^{1}H NMR of **5c**

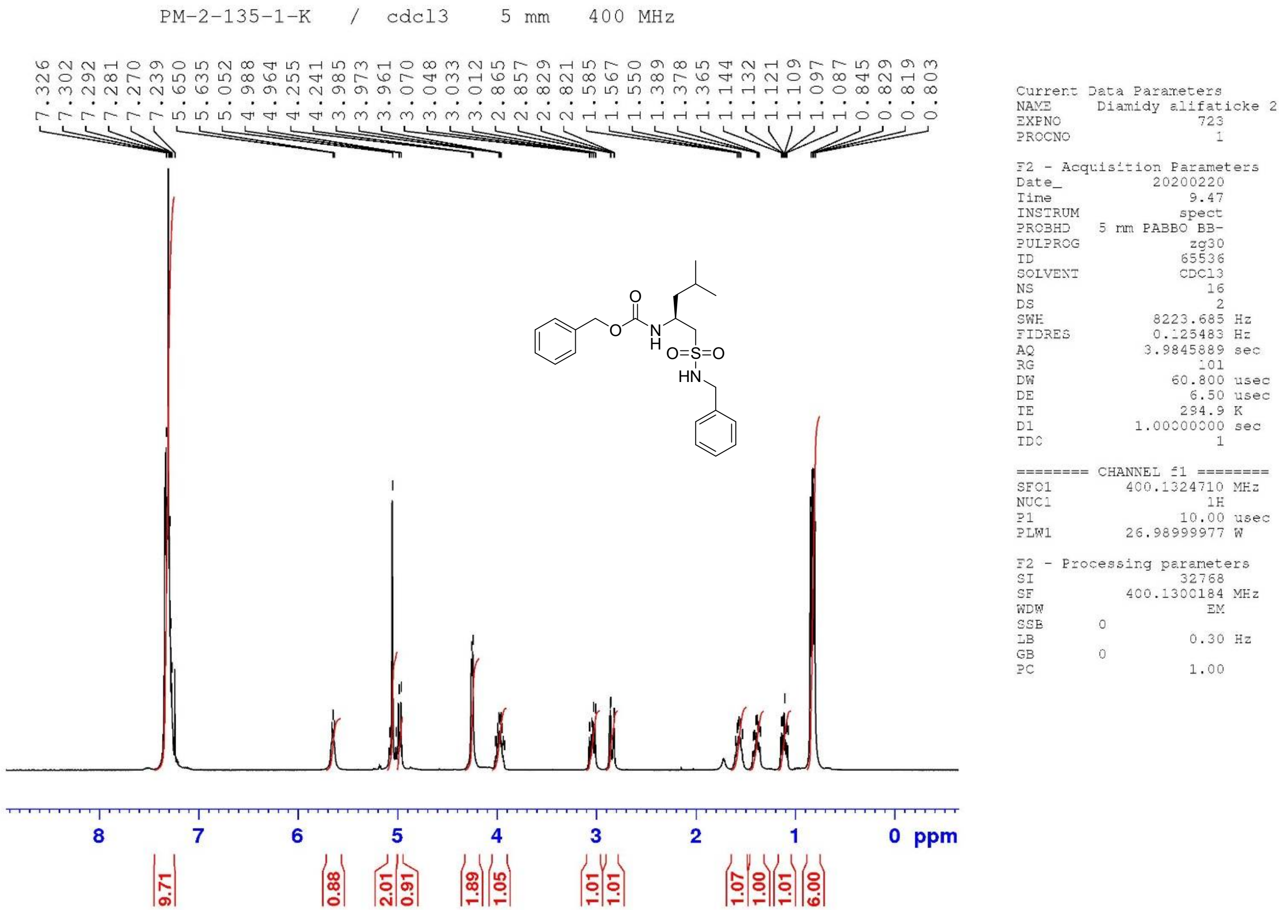

1.24. Copy of ^{13}C NMR of **5c**

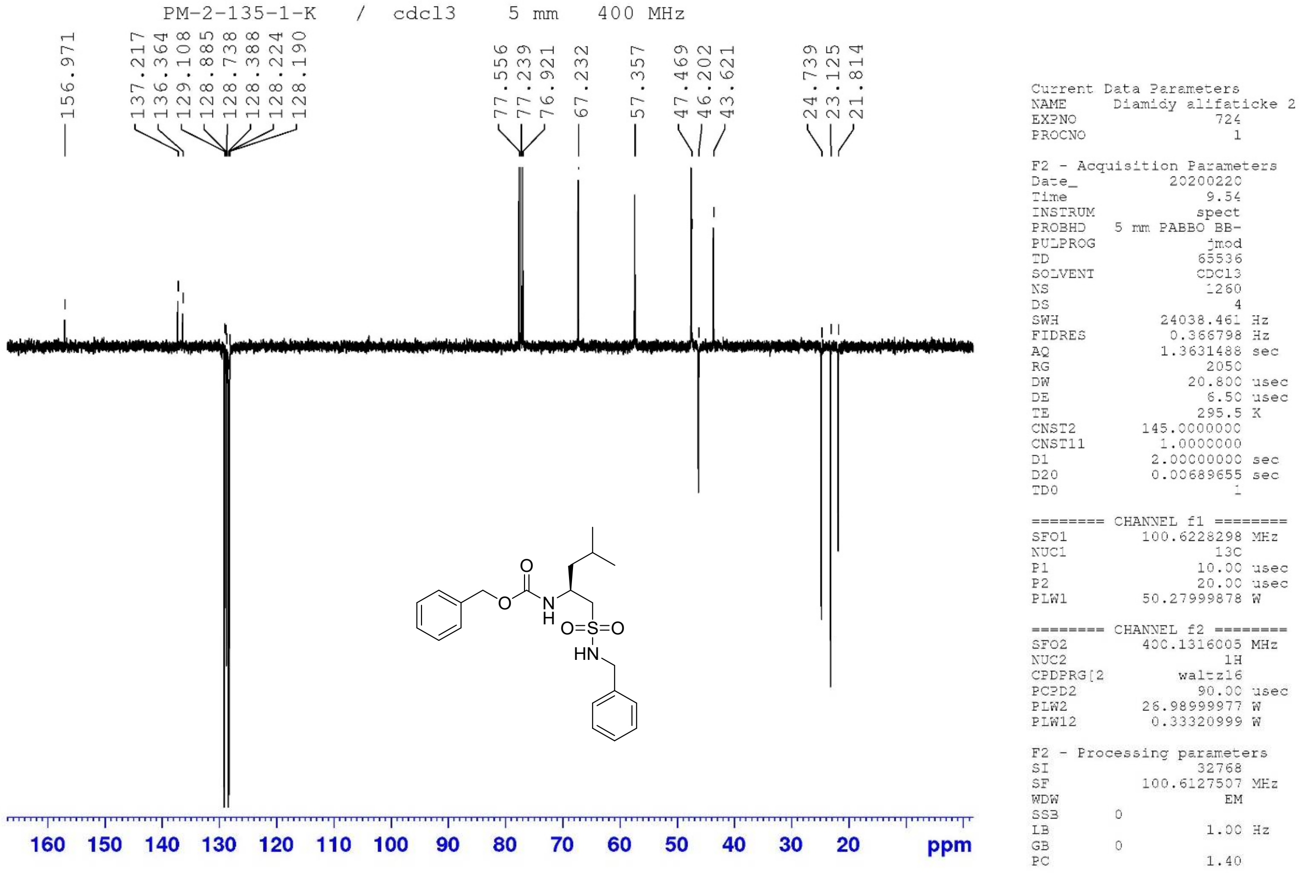

1.25. Copy of ^{1}H NMR of **5d**

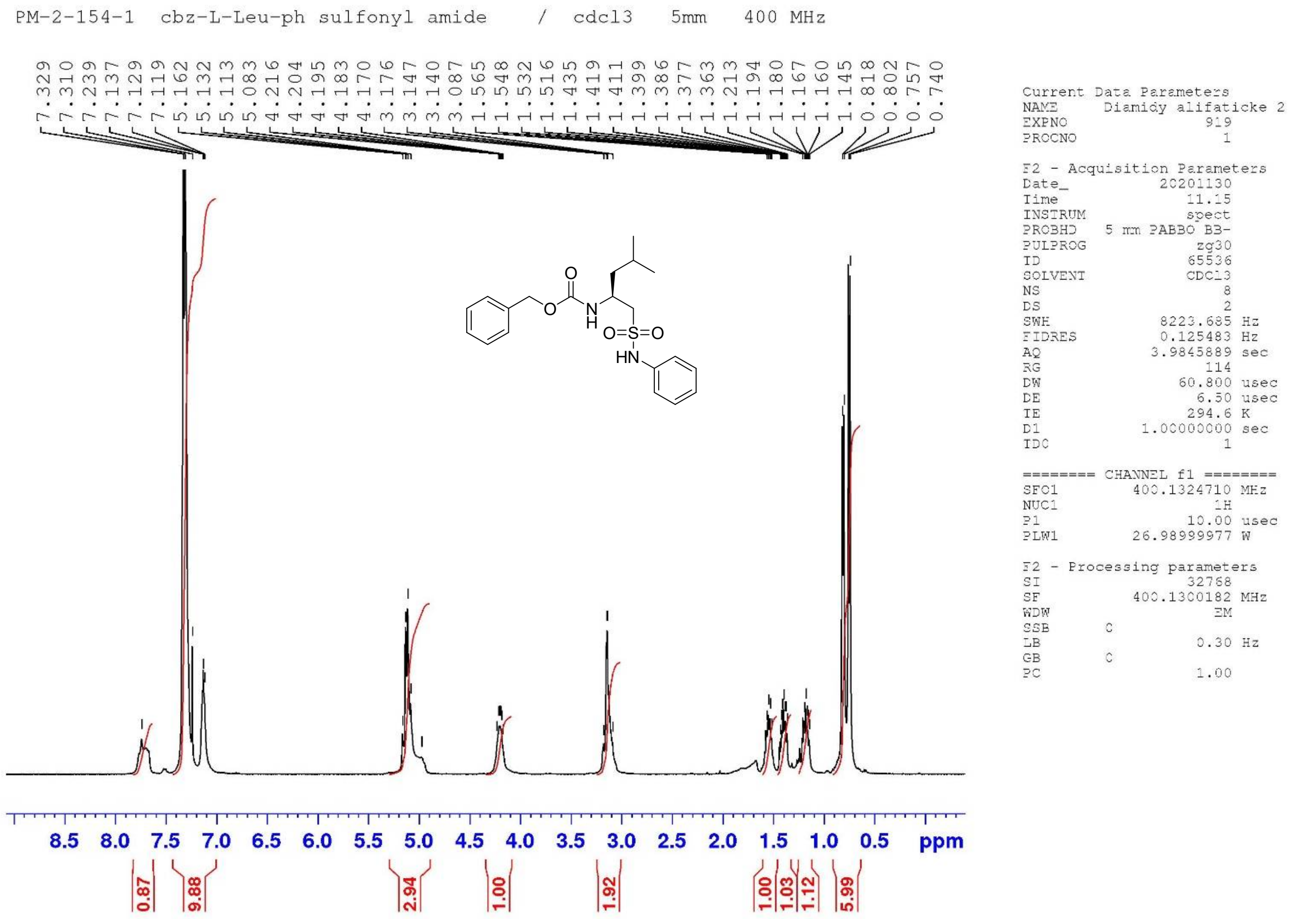

1.26. Copy of ^{13}C NMR of **5d**

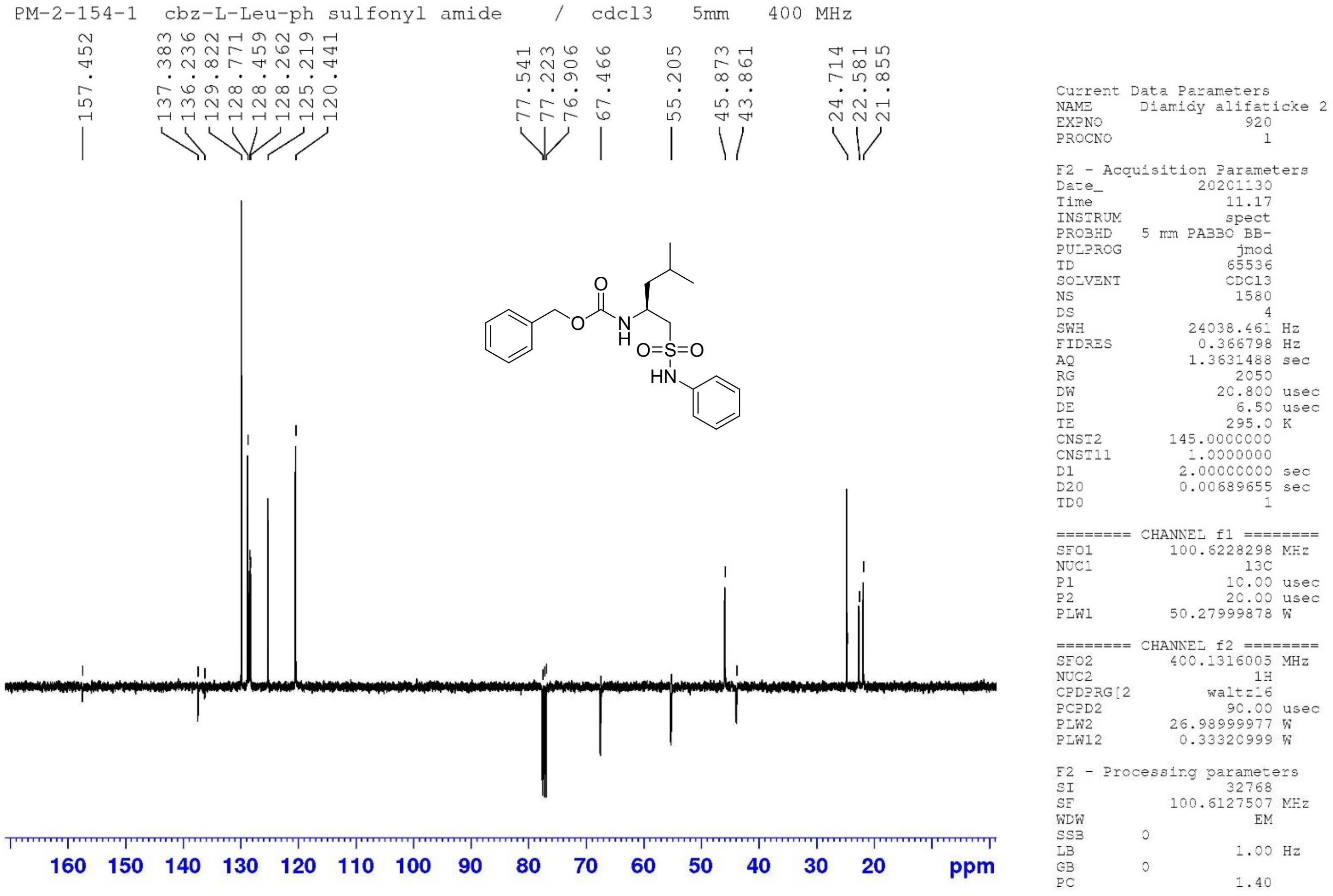

1.27. Copy of ^{1}H NMR of **5e**

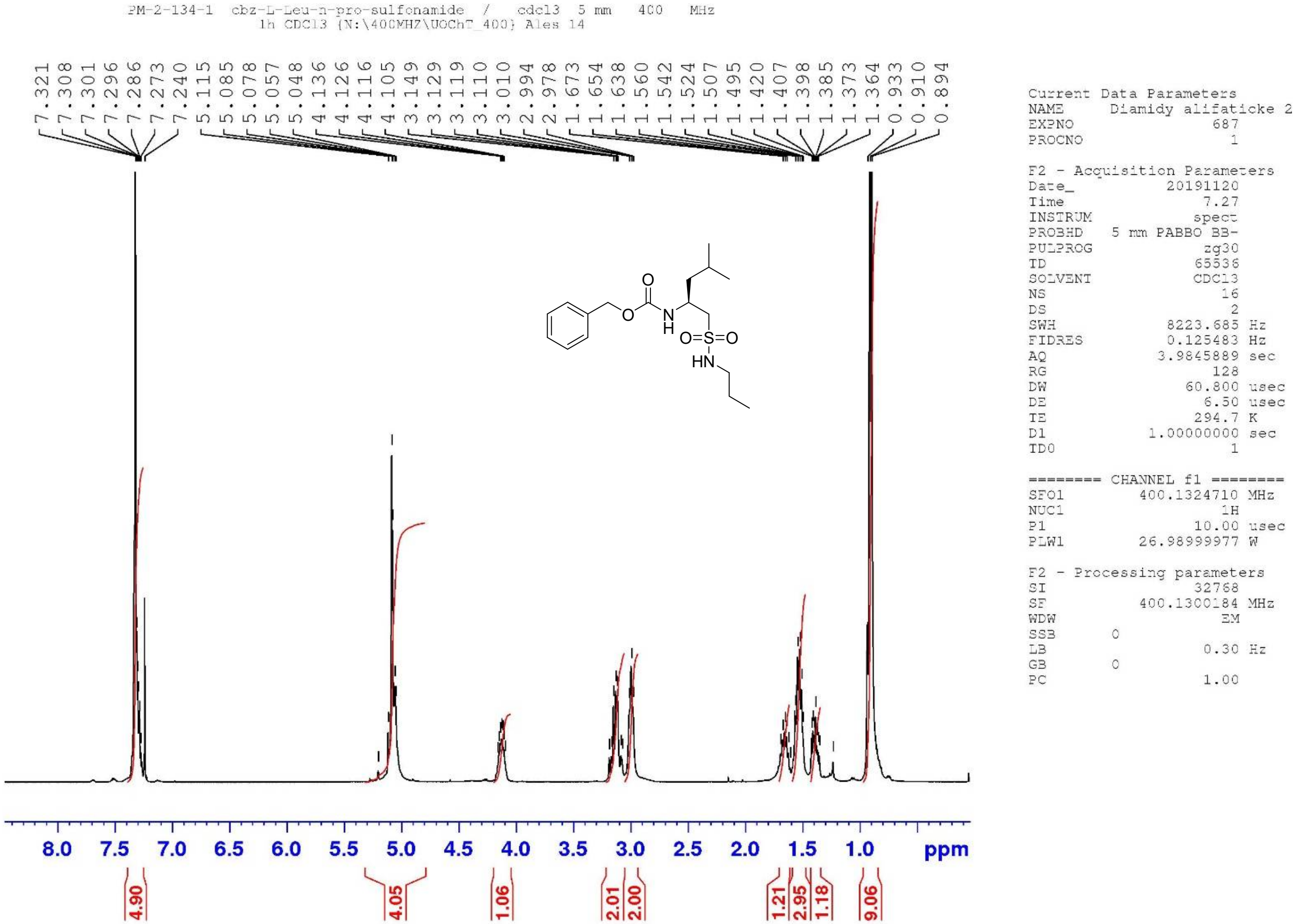

1.28. Copy of ^{13}C NMR of **5e**

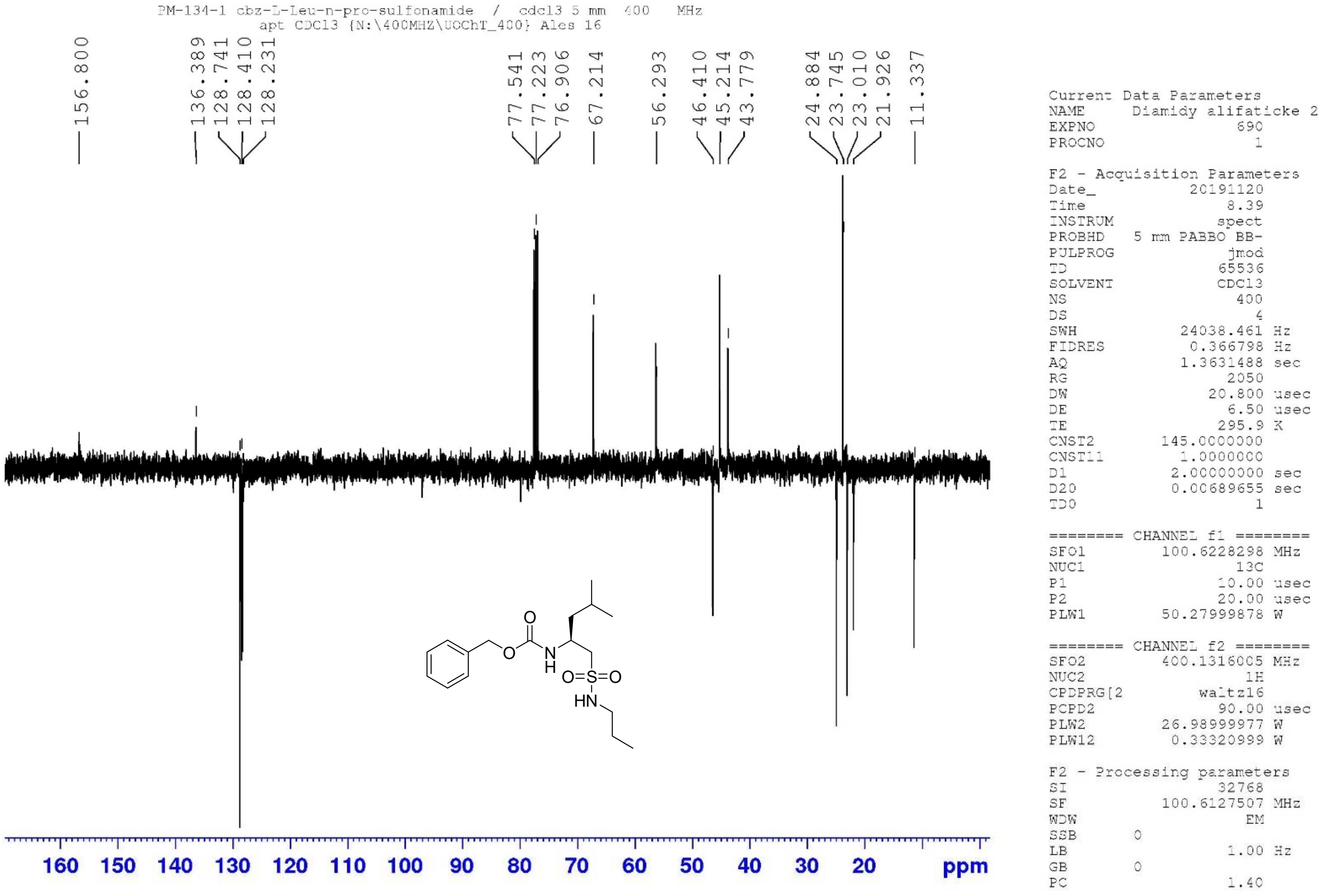

1.29. Copy of ^{1}H NMR of **5f**

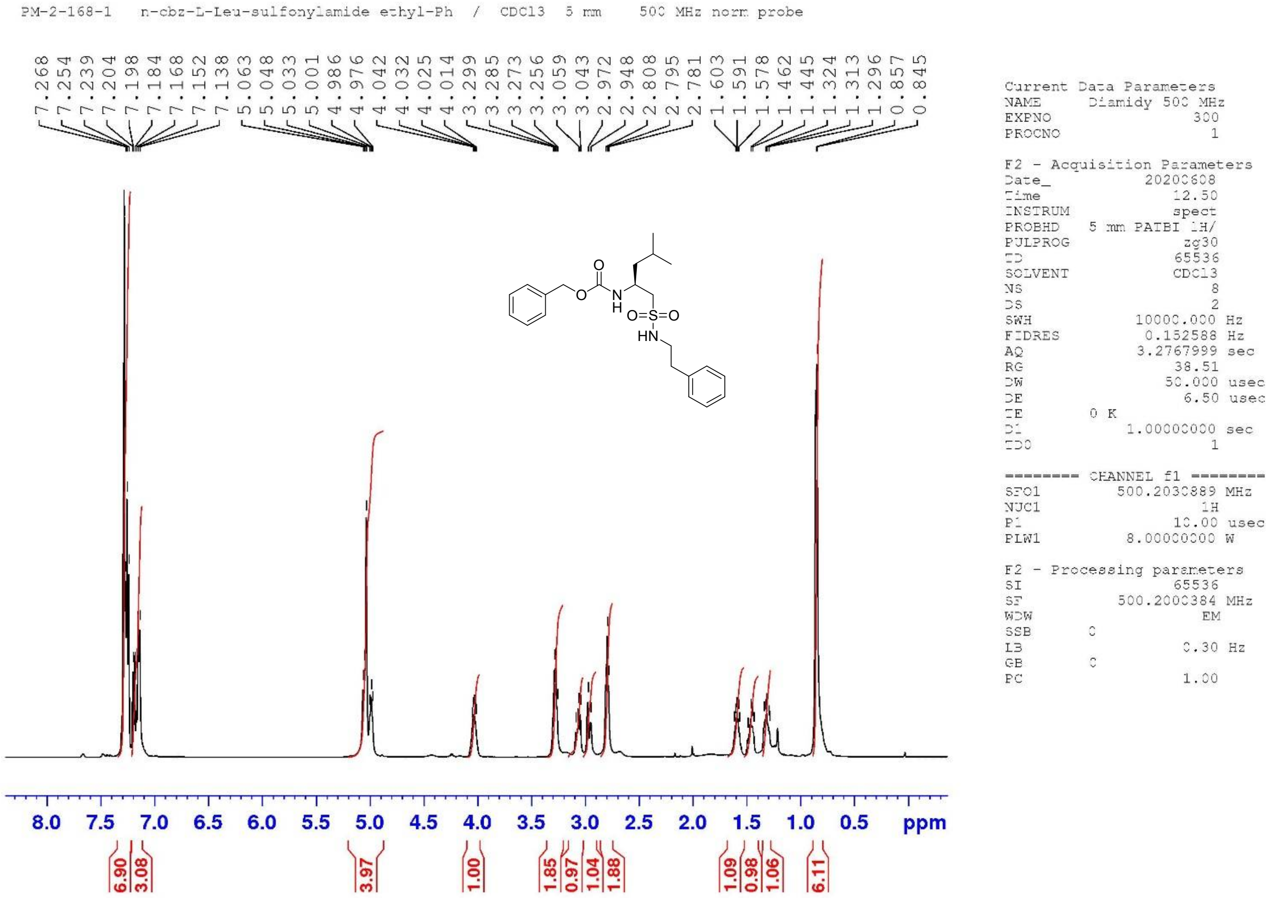

1.30. Copy of ^{13}C NMR of **5f**

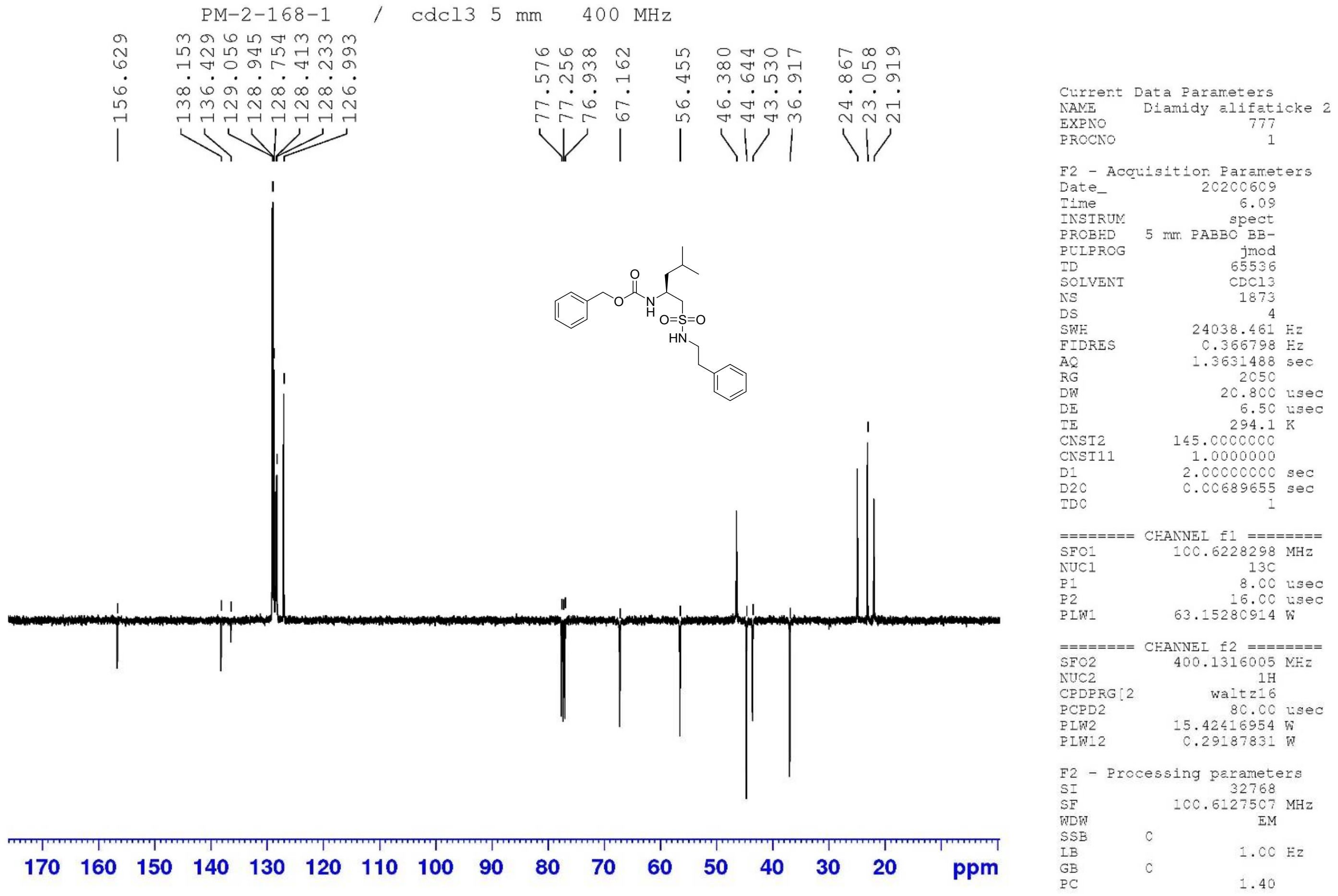

1.31. Copy of ^{1}H NMR of **5g**

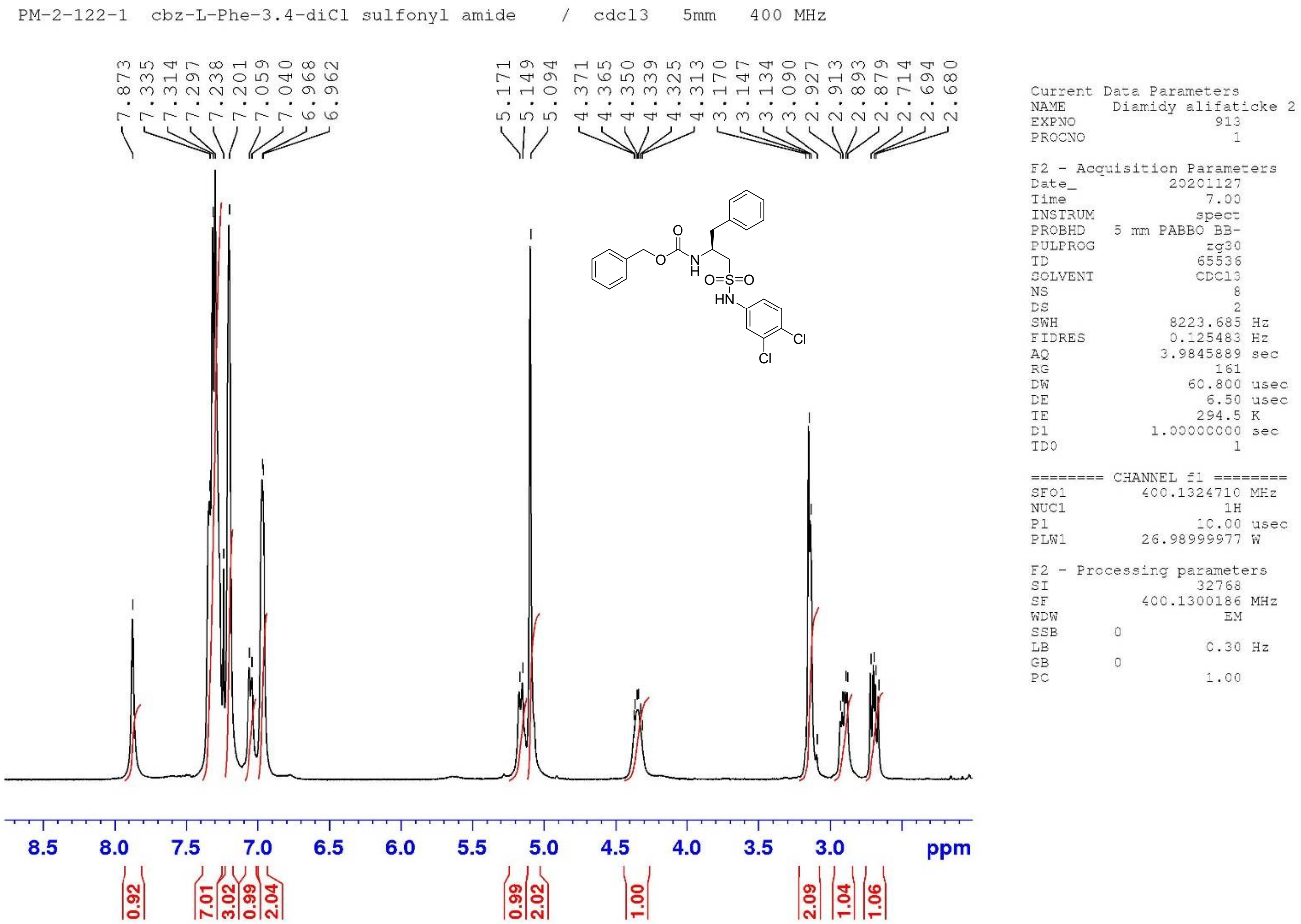

1.32. Copy of ^{13}C NMR of **5g**

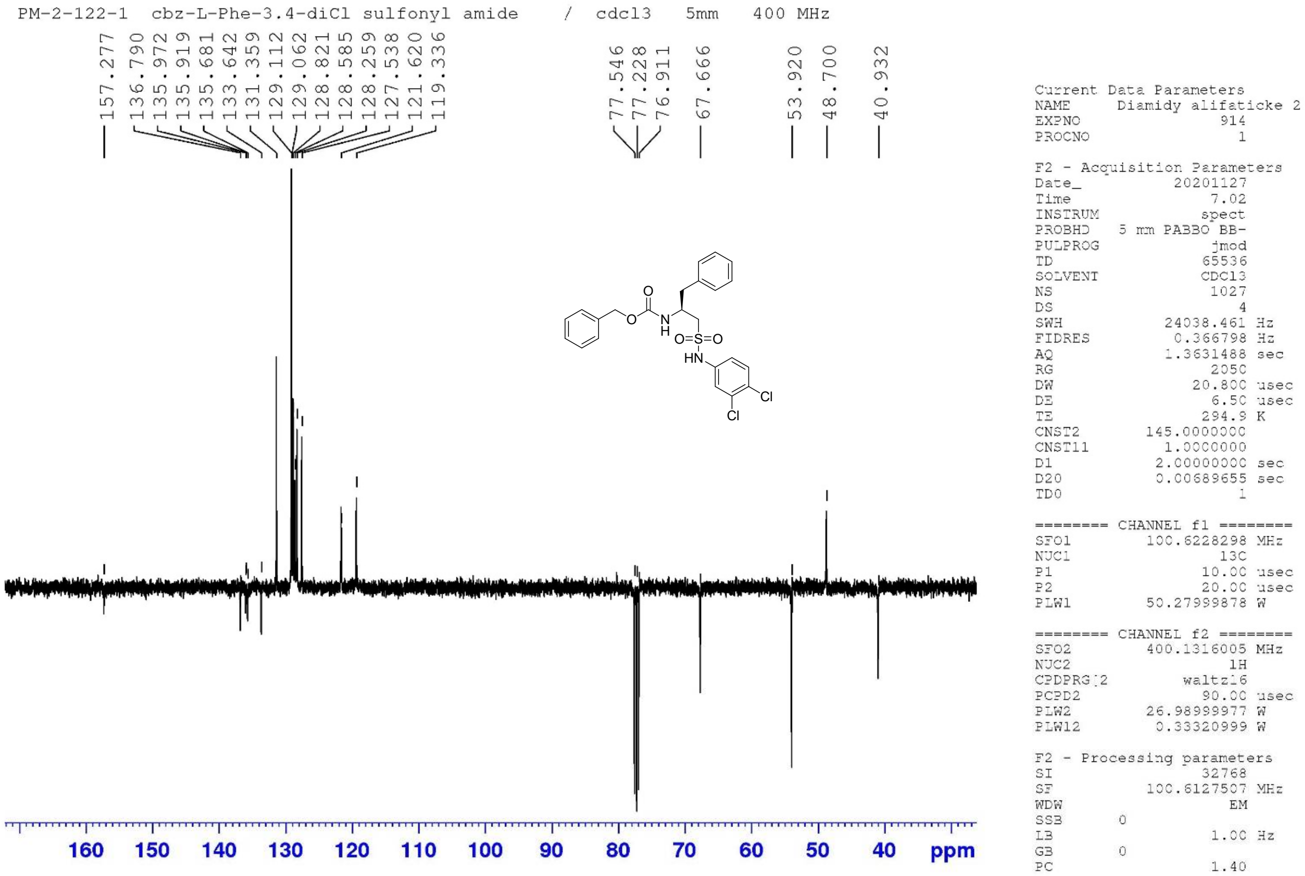

1.33. Copy of ^{1}H NMR of **5h**

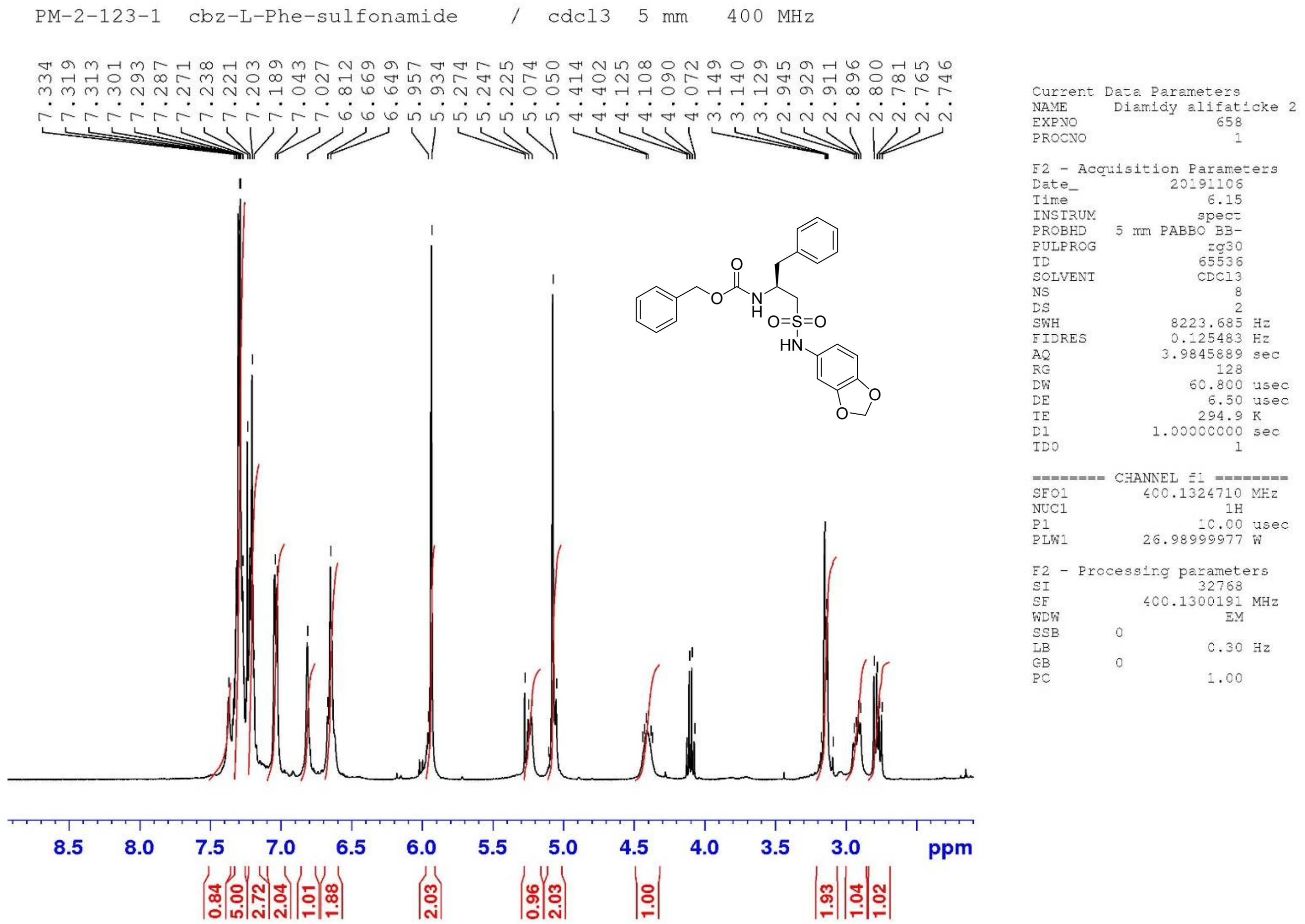

1.34. Copy of ^{13}C NMR of **5h**

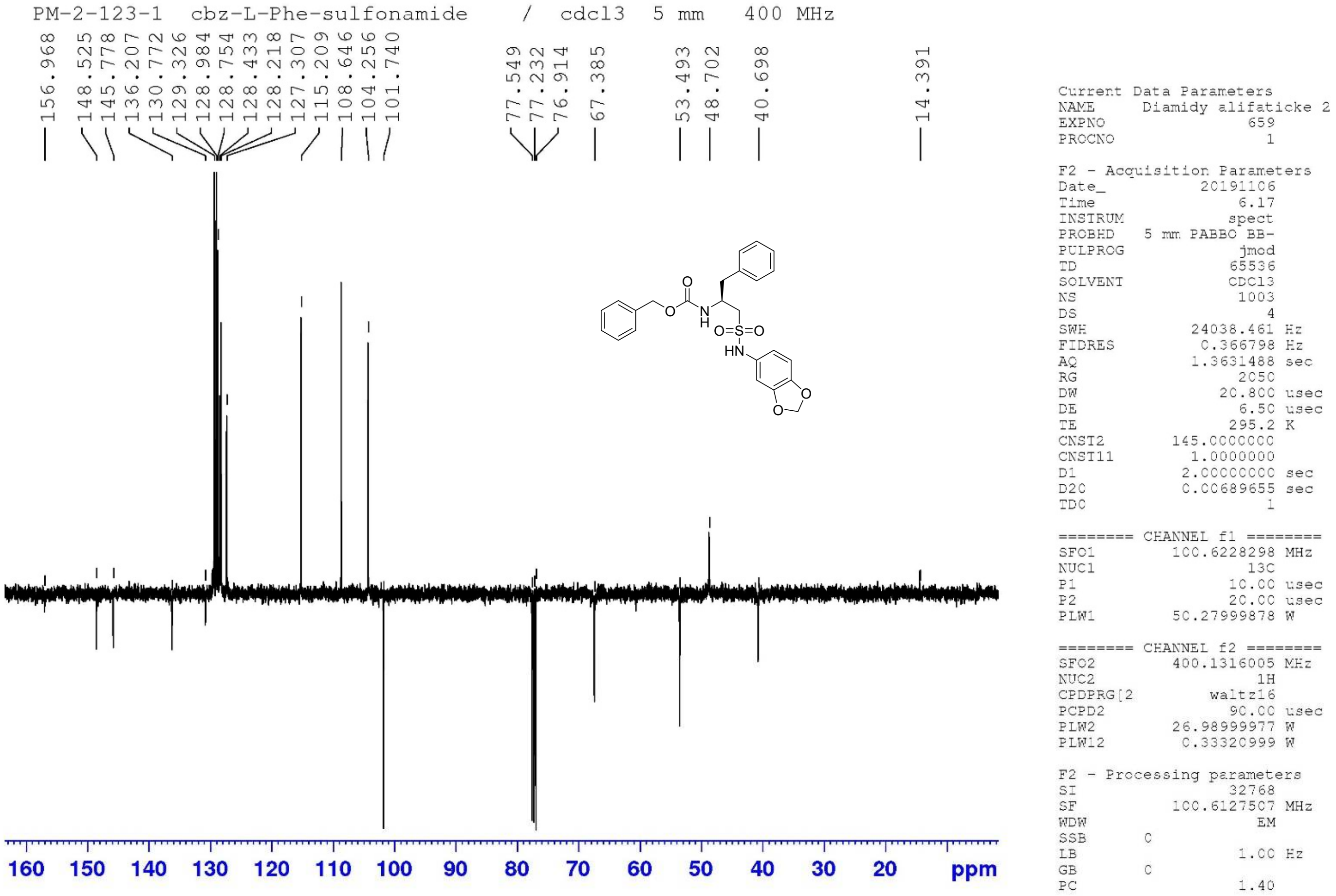

1.35. Copy of ^{1}H NMR of **5i**

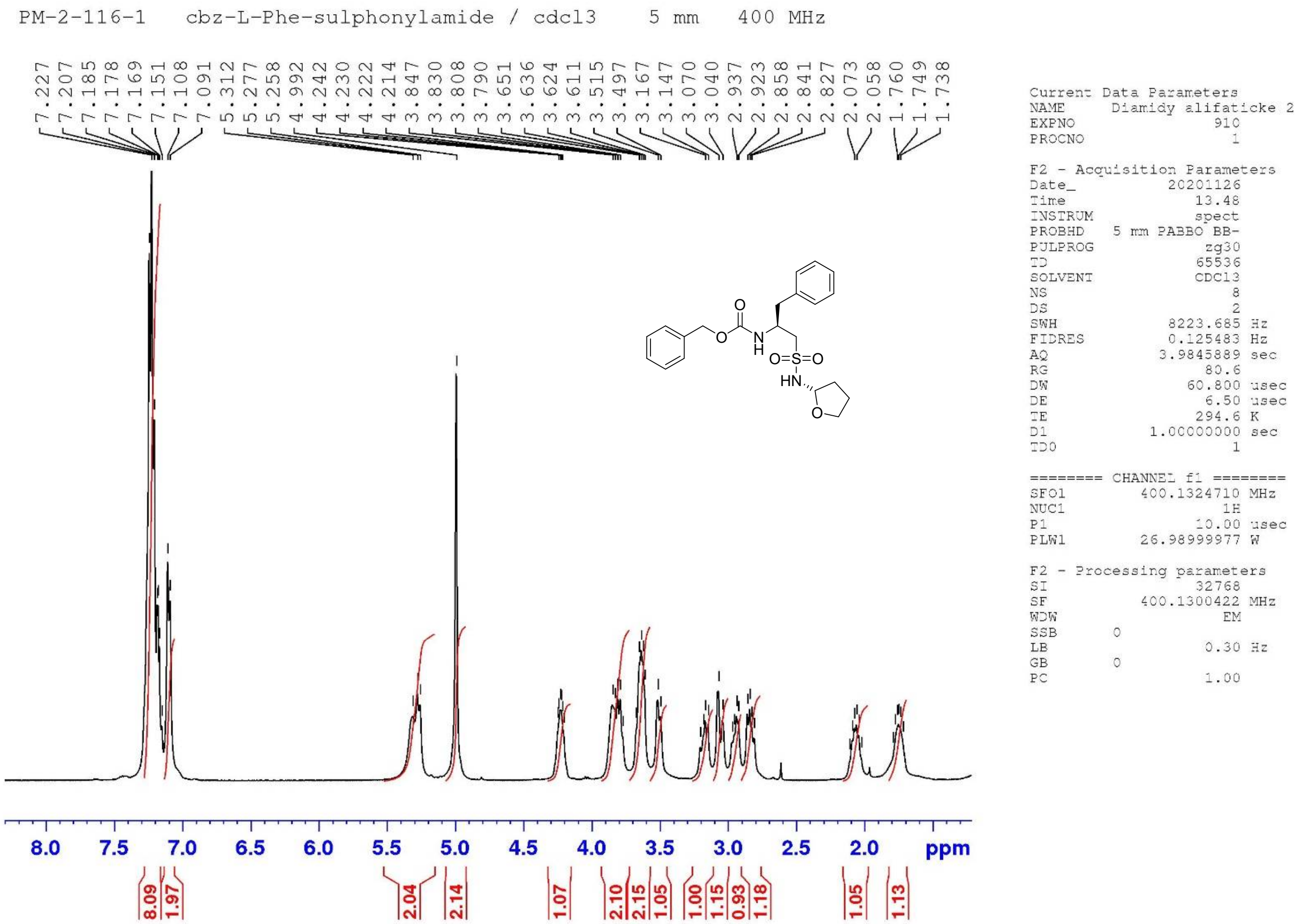

1.36. Copy of ^{13}C NMR of **5i**

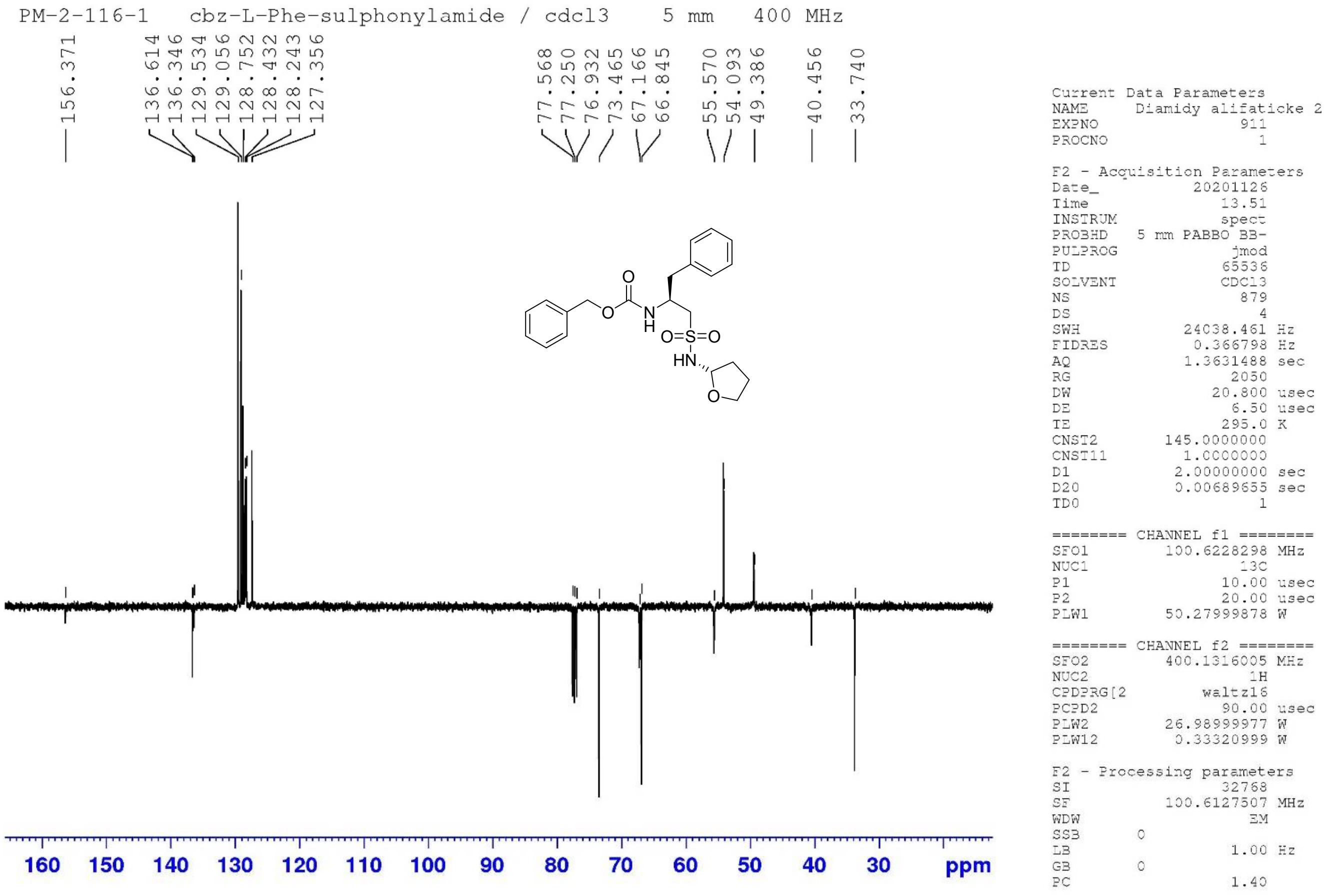

1.37. Copy of ^{1}H NMR of **5j**

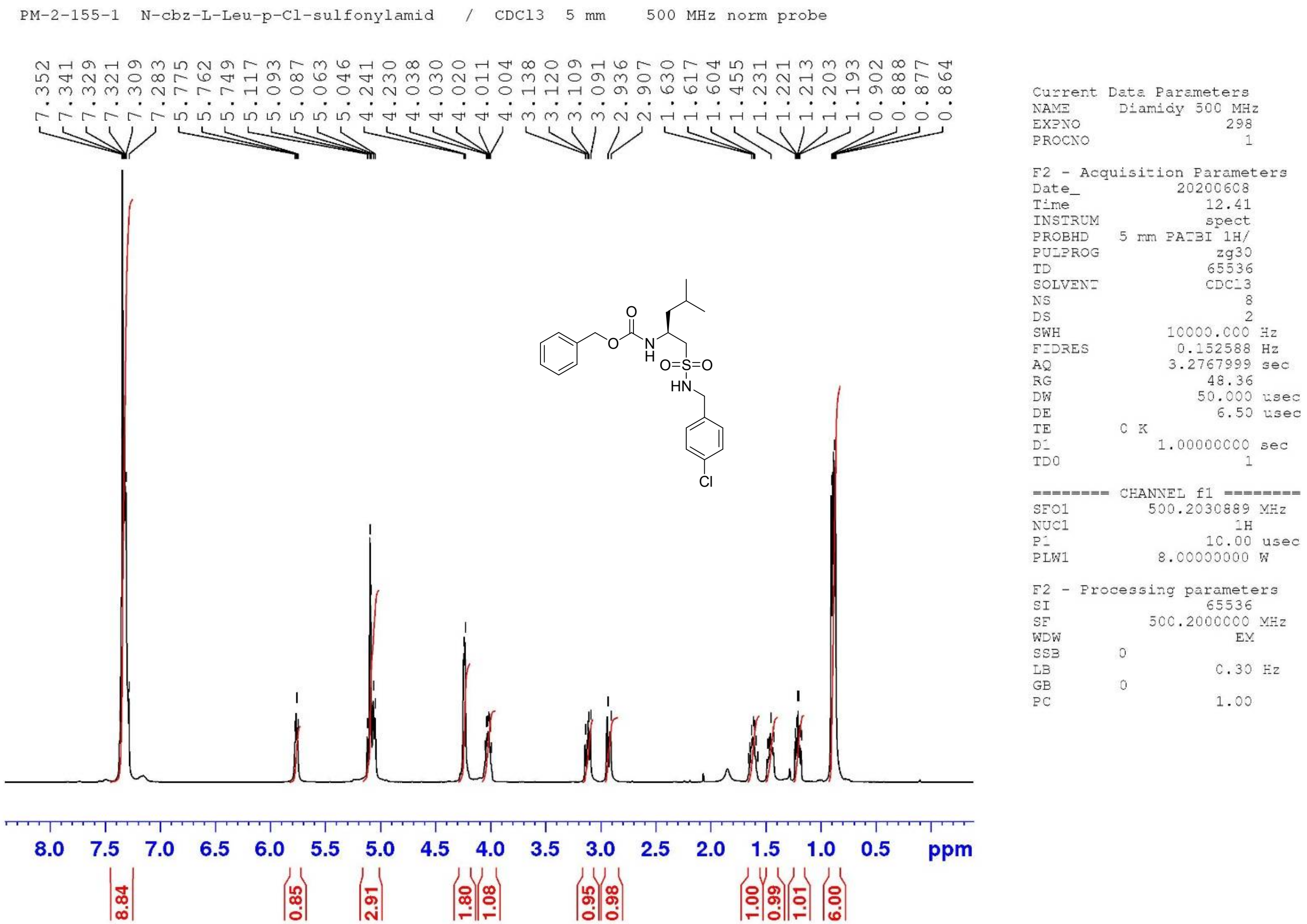

1.38. Copy of ^{13}C NMR of **5j**

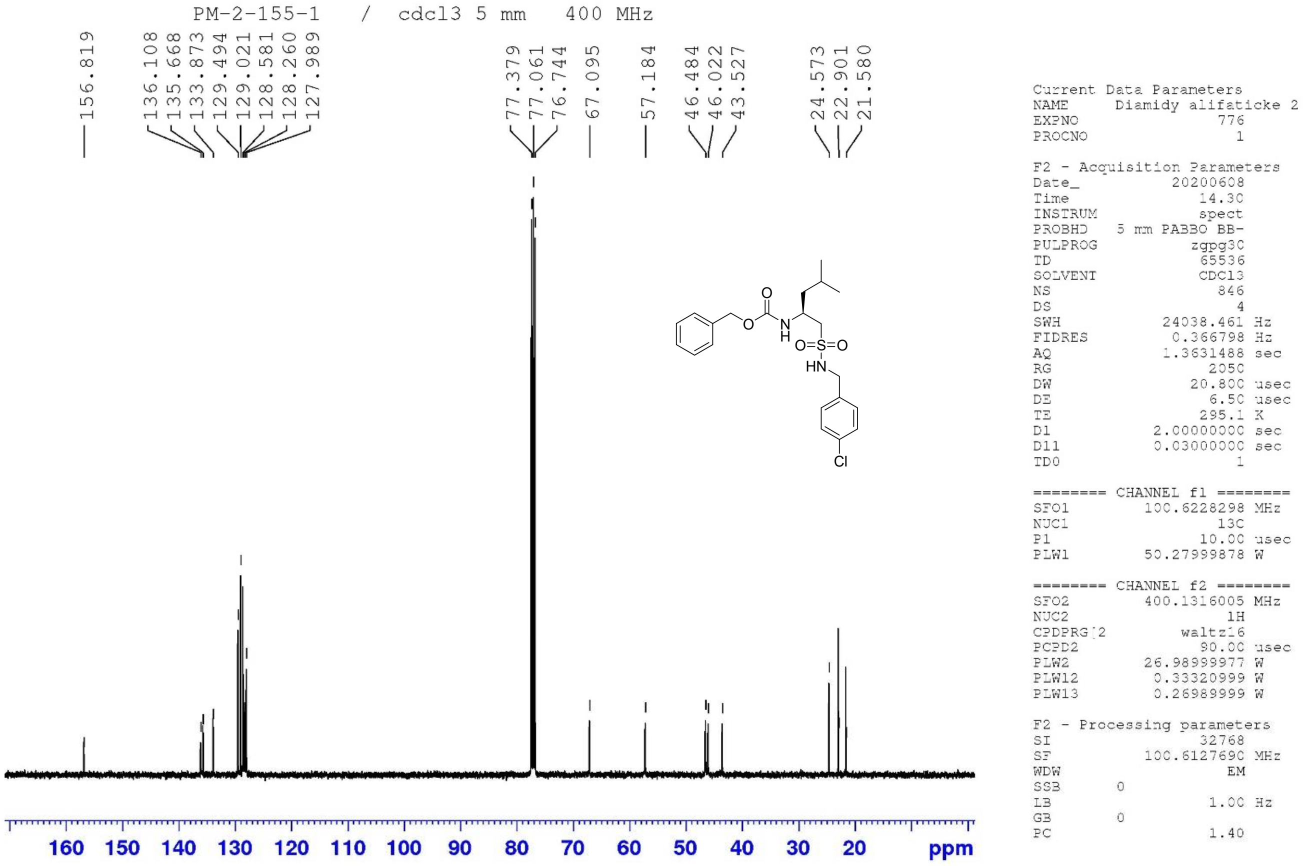

1.39. Copy of ^{1}H NMR of **5k**

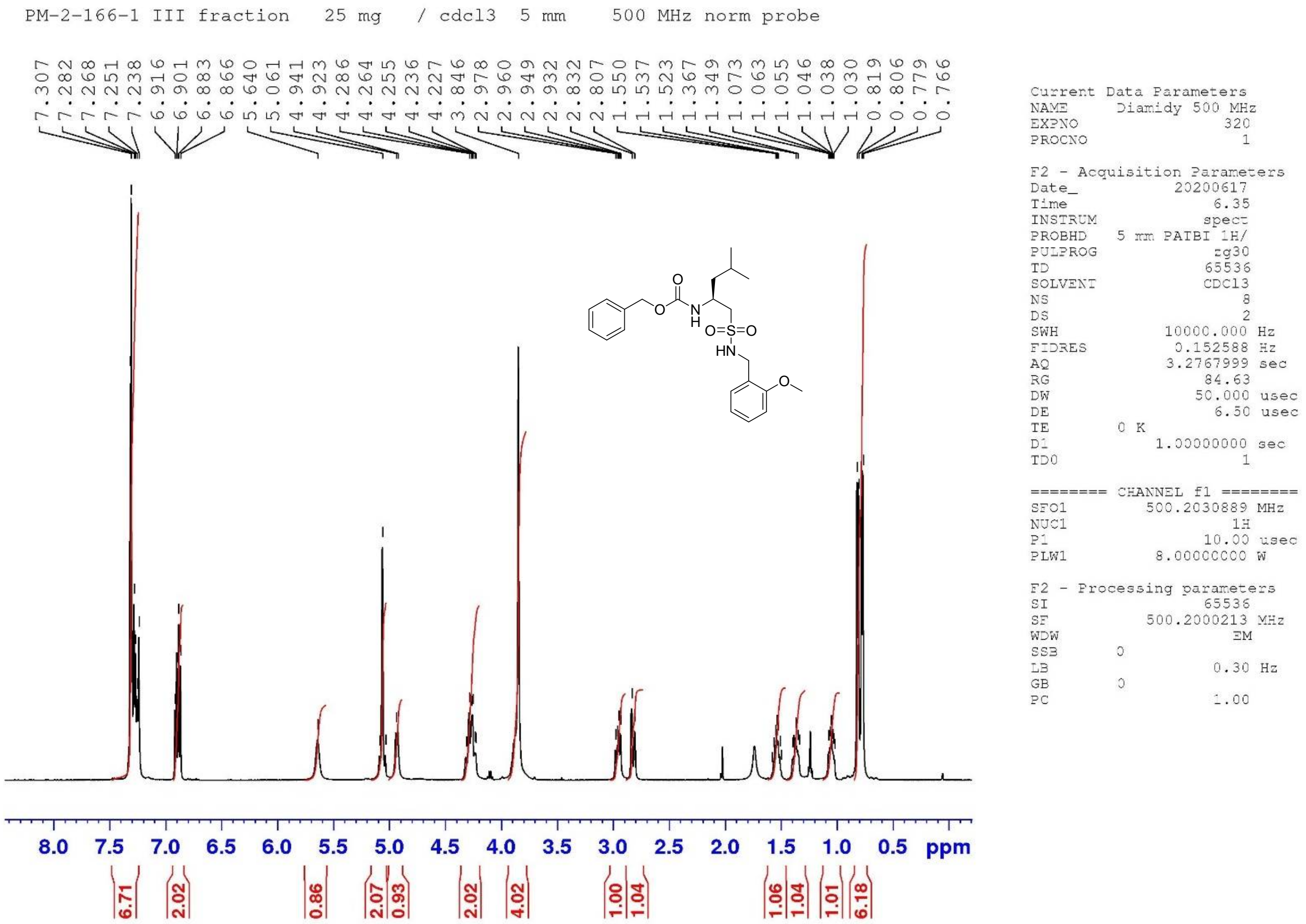

1.40. Copy of ^{13}C NMR of **5k**

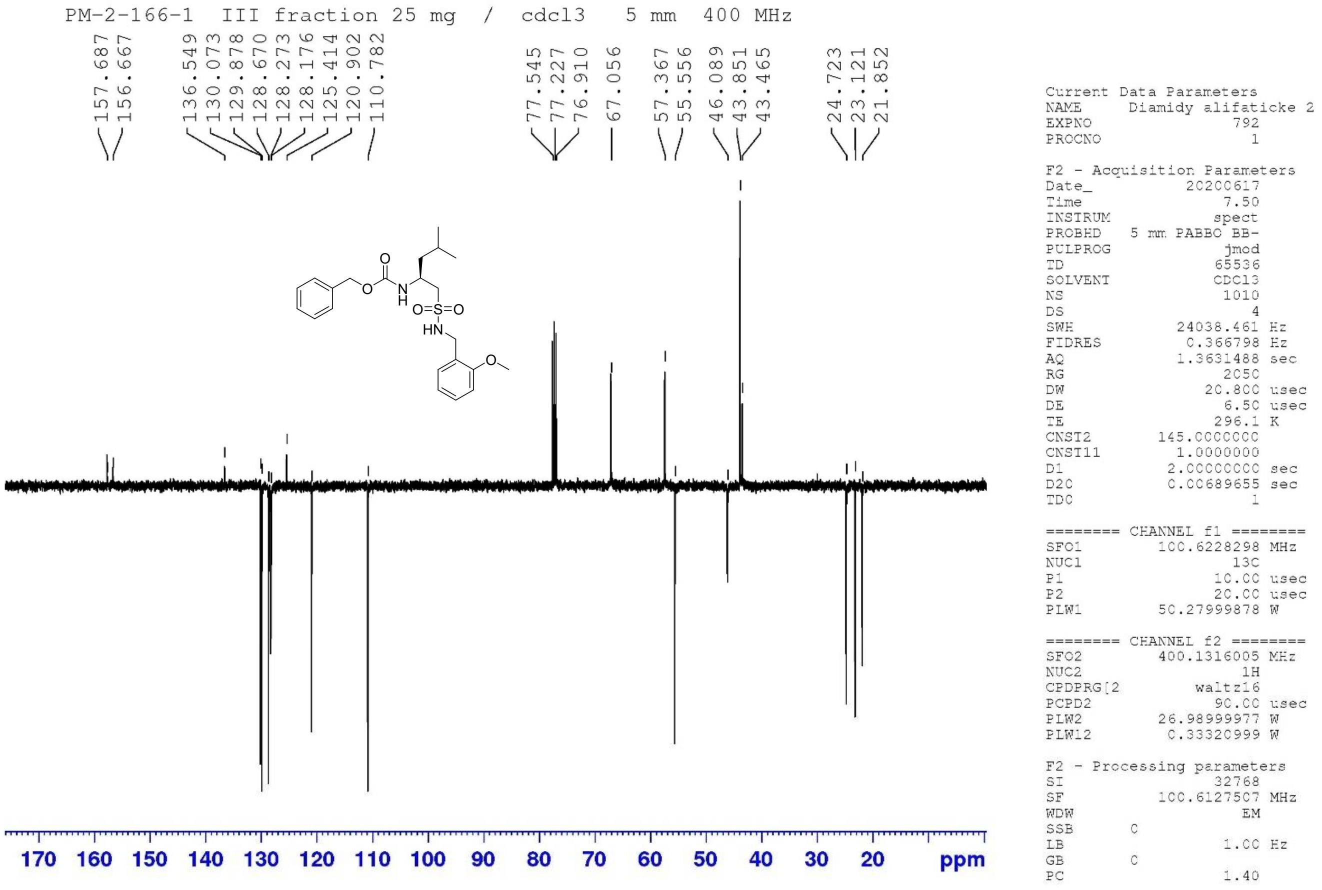

1.41. Copy of ^{1}H NMR of **5l**

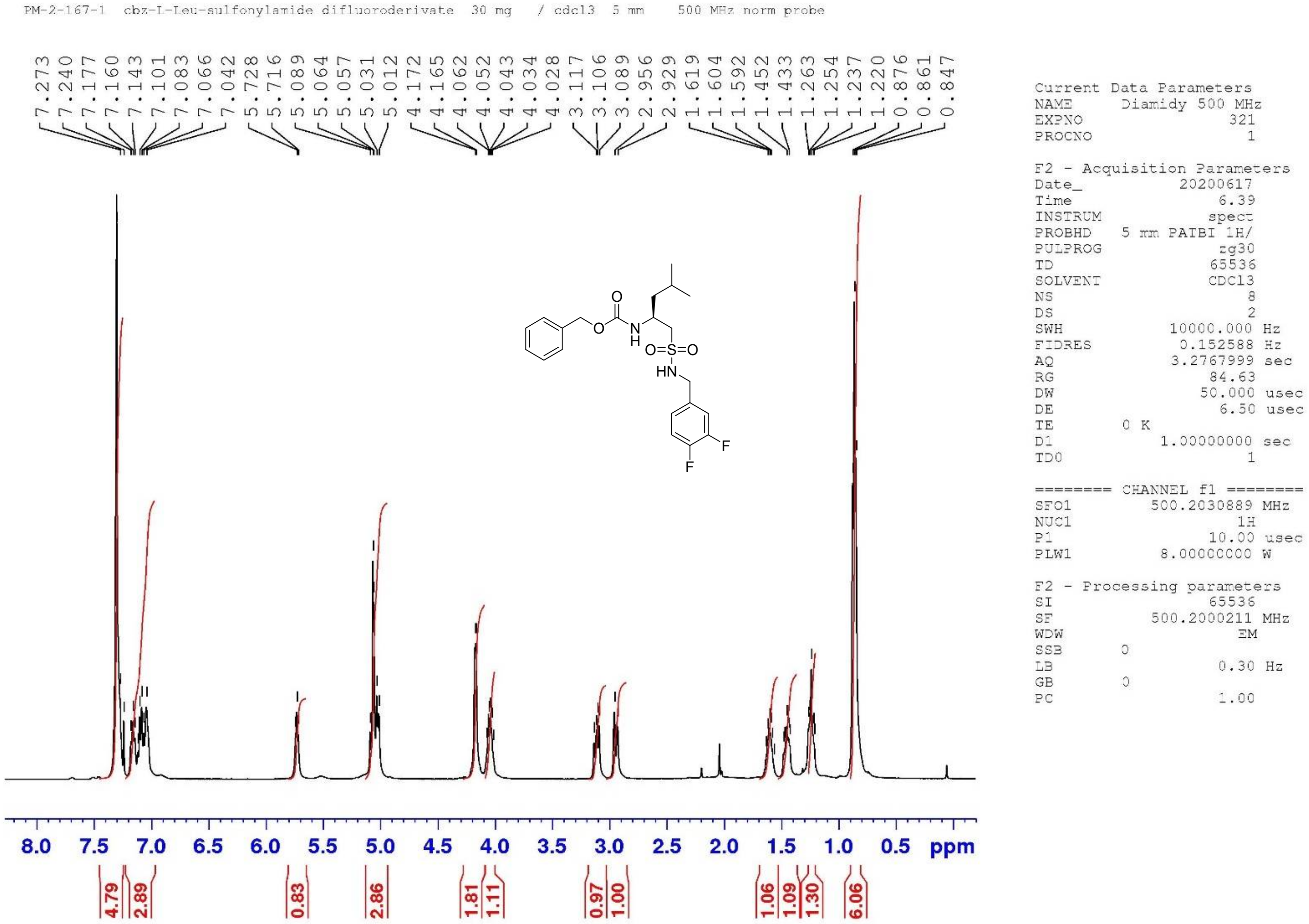

1.42. Copy of ^{13}C NMR of **5l**

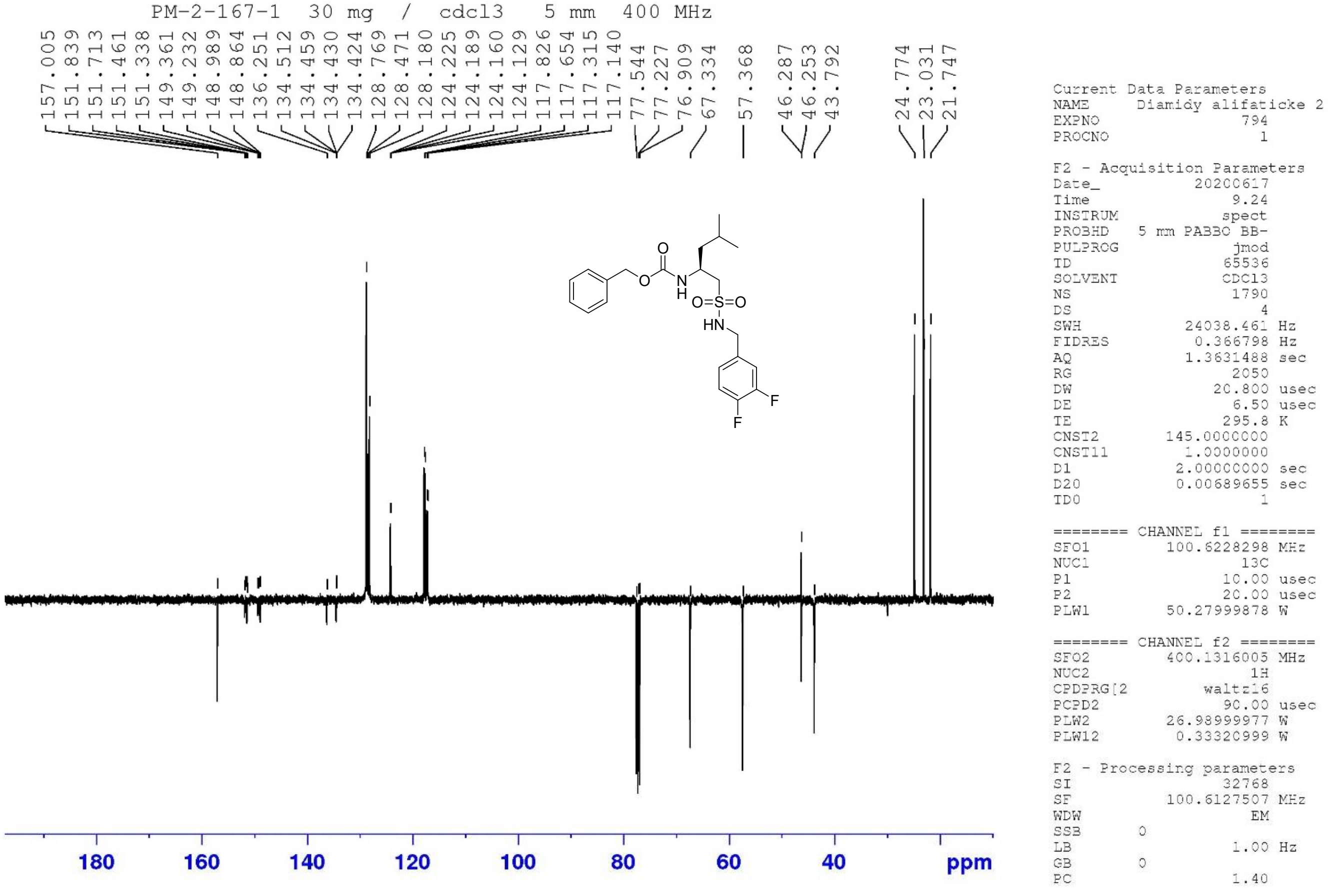

1.43. Copy of ^{19}F NMR of **5l**

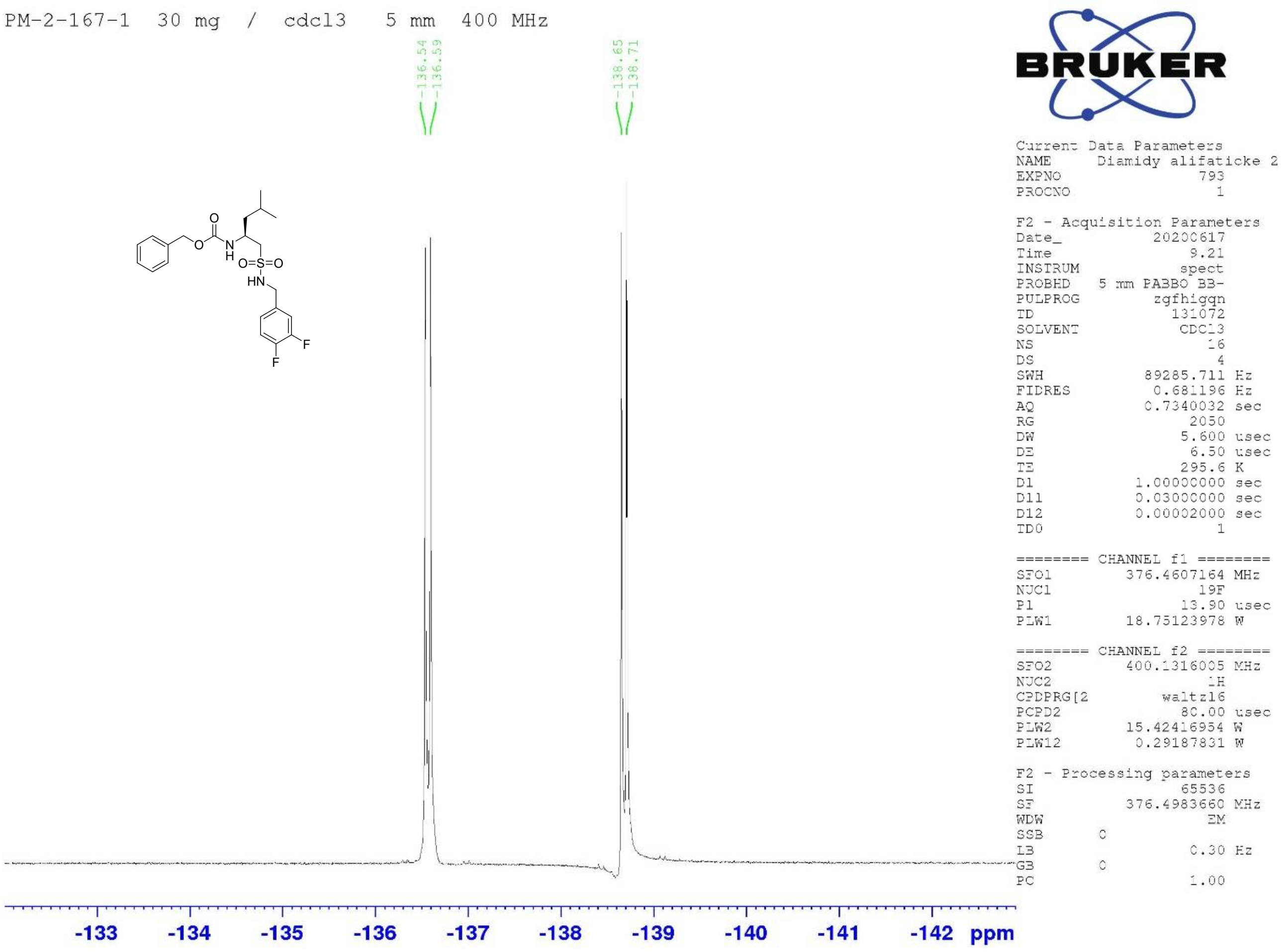

1.44. Copy of ^{1}H NMR of **5m**

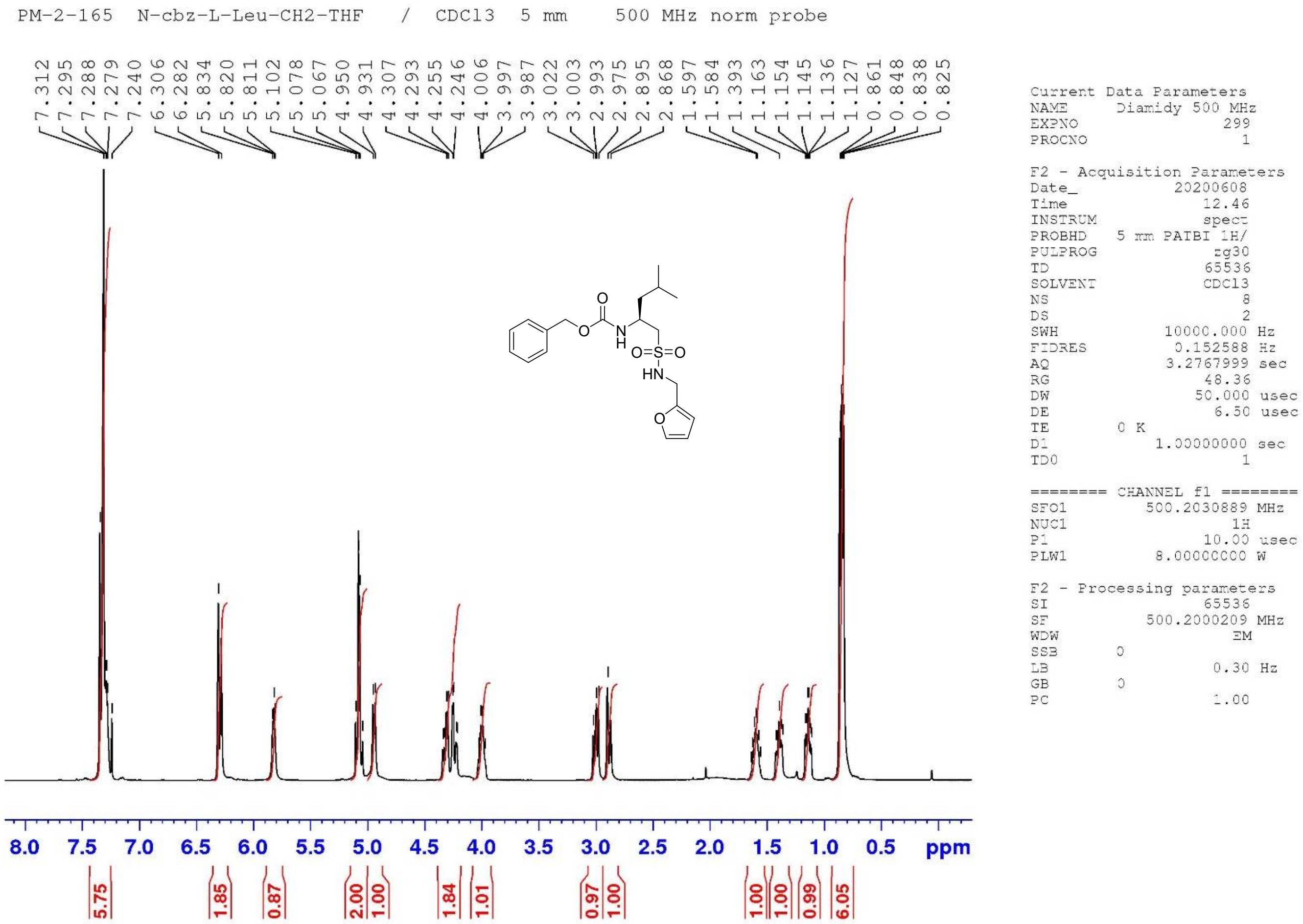

1.45. Copy of ^{13}C NMR of **5m**

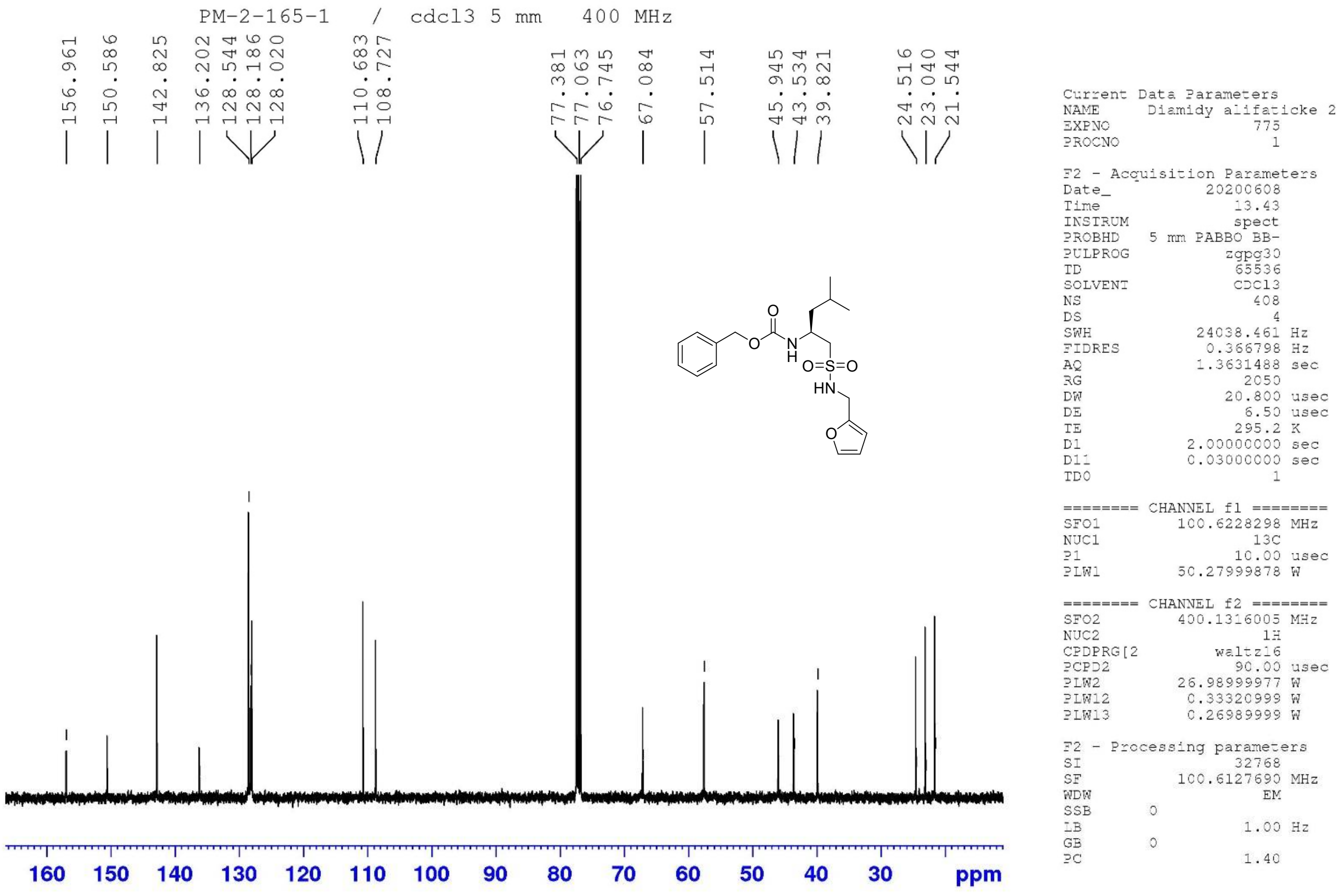

1.46. Copy of ^{1}H NMR of **5n**

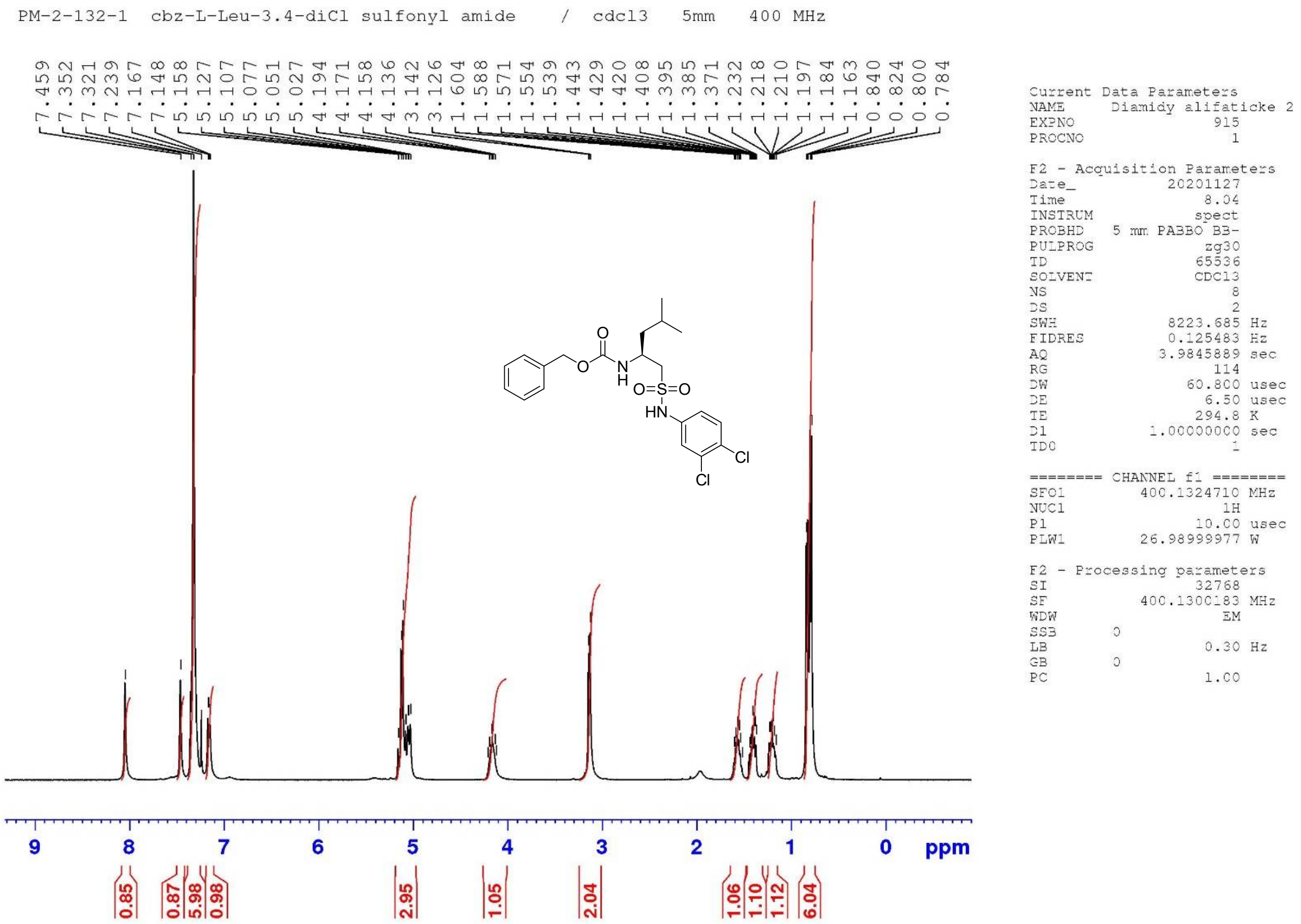

1.47. Copy of ^{13}C NMR of **5n**

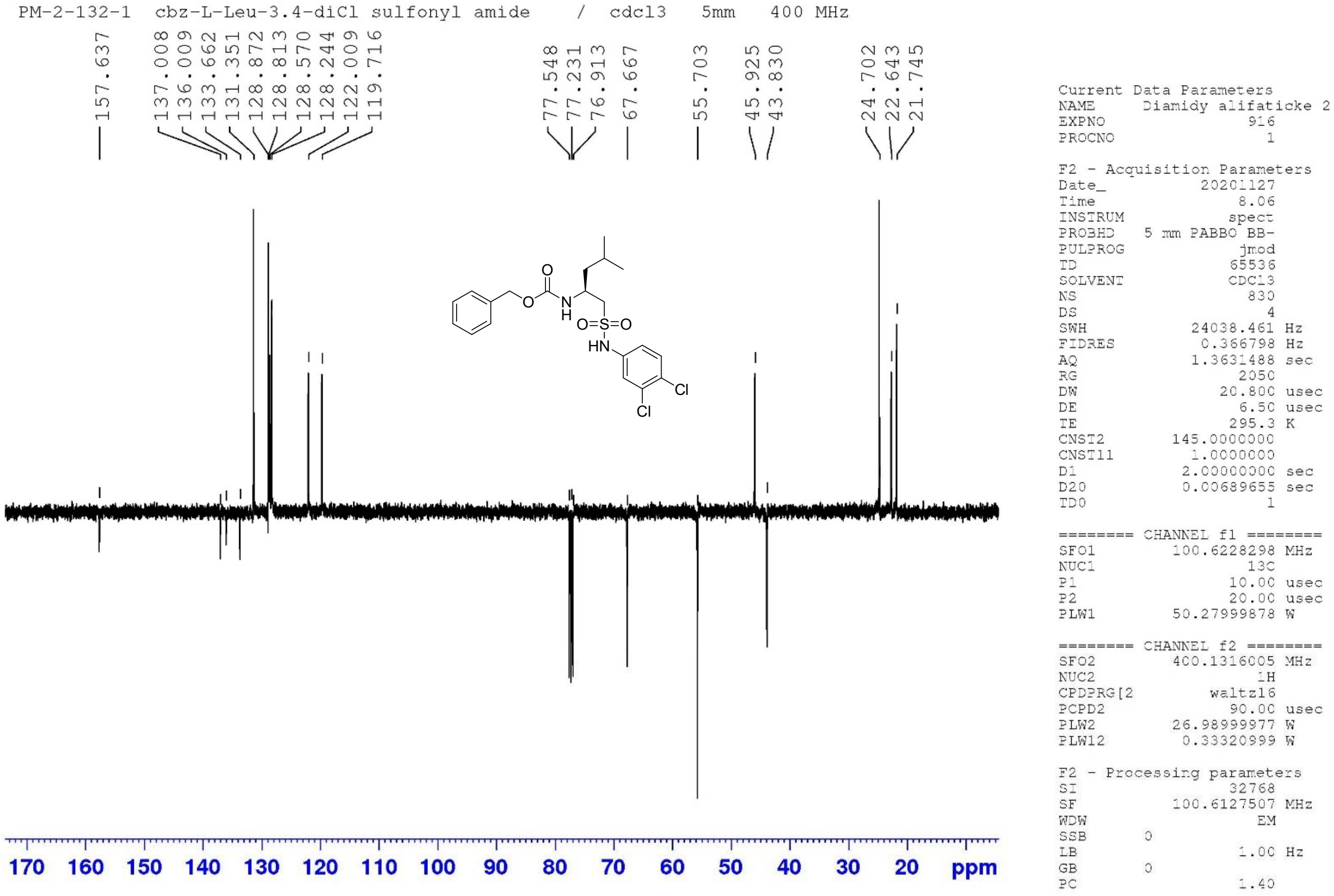

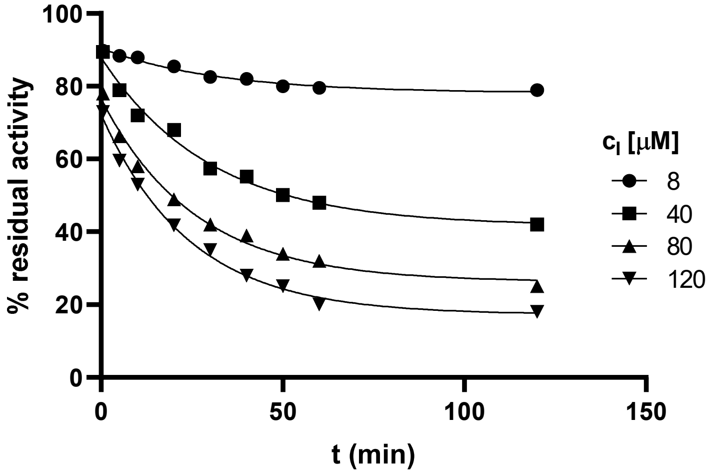

Figure S2 The dependence % residual BChE activity vs. time in the presence of **5k**. The points are expressed as the mean of at least two separate experiments.

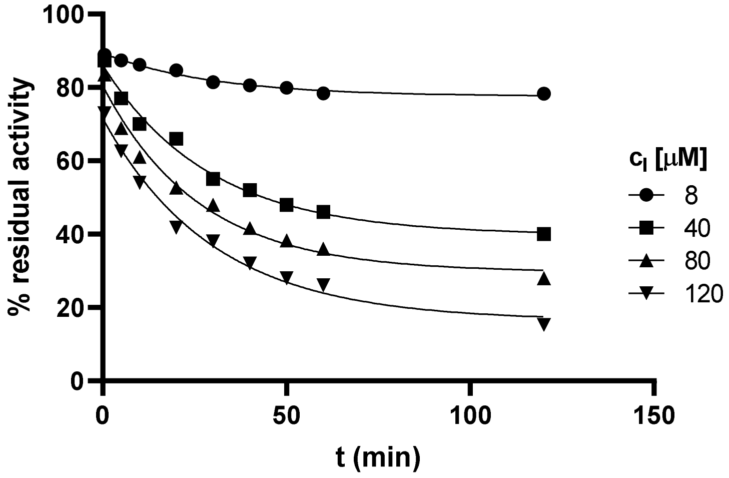

Figure S3 The dependence % residual BChE activity vs. time in the presence of **5j**. The points are expressed as the mean of at least two separate experiments.

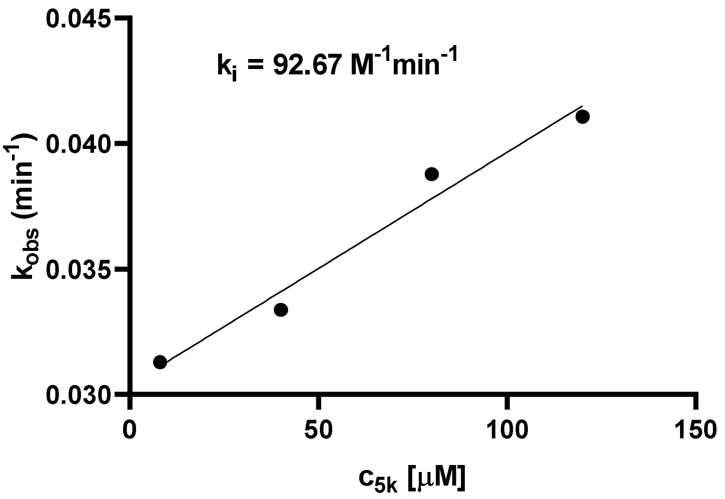

Figure S4 The dependence pseudo-first-order inhibition rates vs. concentration of **5k.**

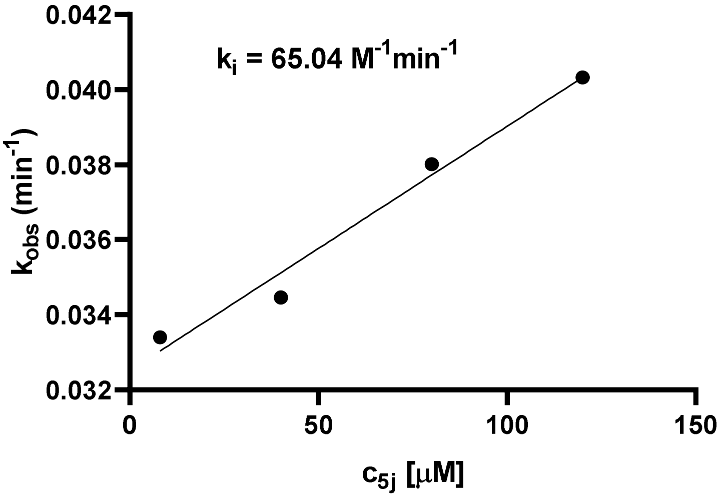

Figure S5 The dependence pseudo-first-order inhibition rates vs. concentration of **5j.**

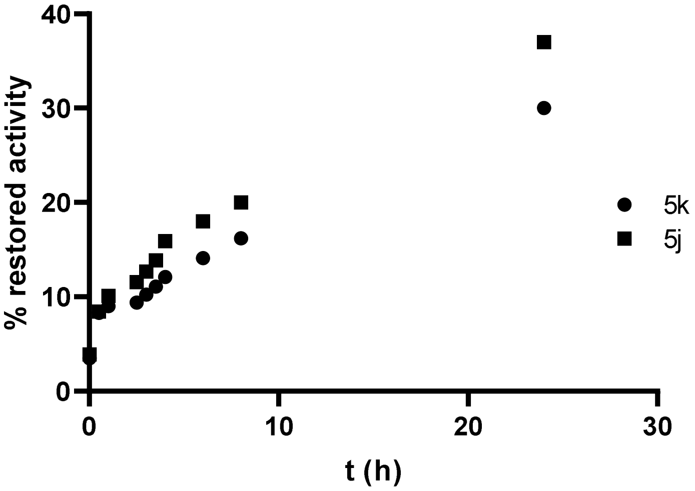

Figure S6 Reactivation of BChE inhibited by **5k** and **5j.** The points are expressed as the mean of at least two separate experiments.

Preliminary stability test of compound **5k** (HPLC results)

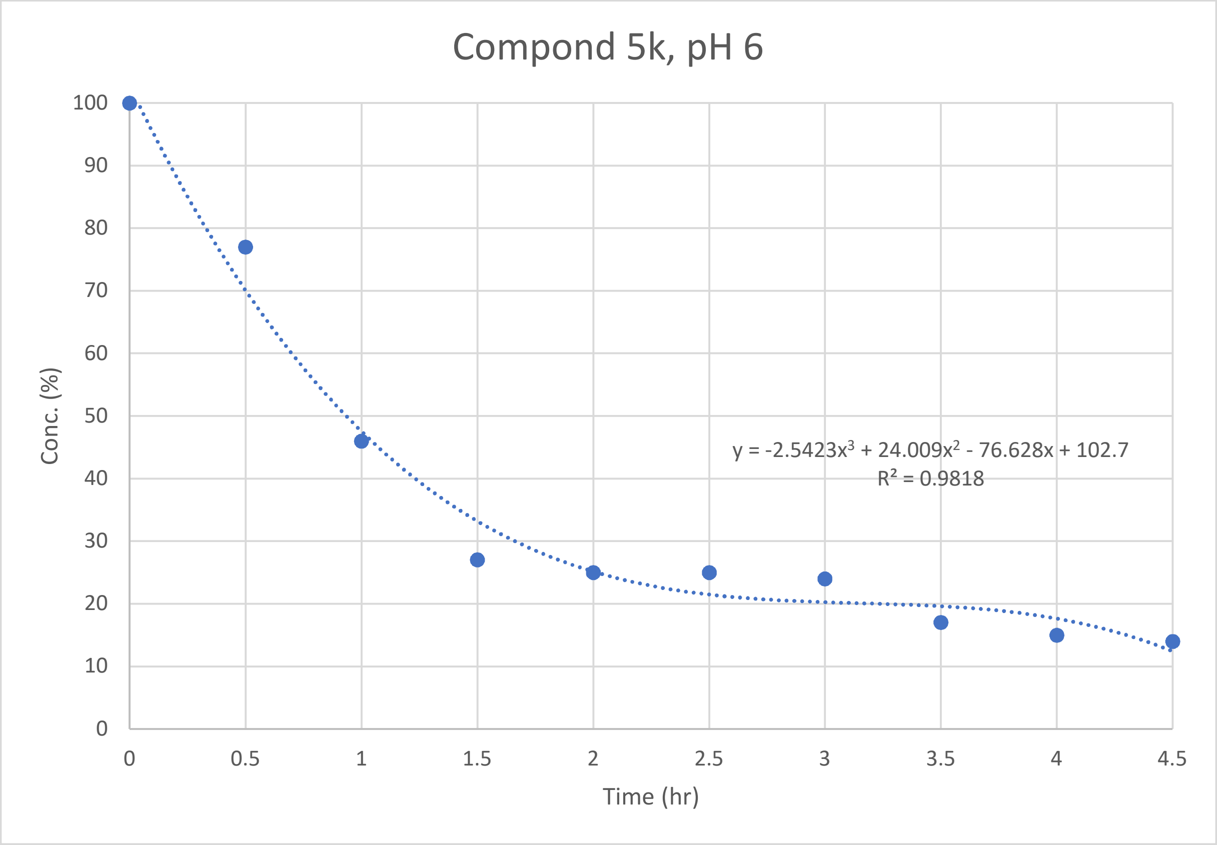

Fig. 1. Decomposition study of compound **5k** at pH 6

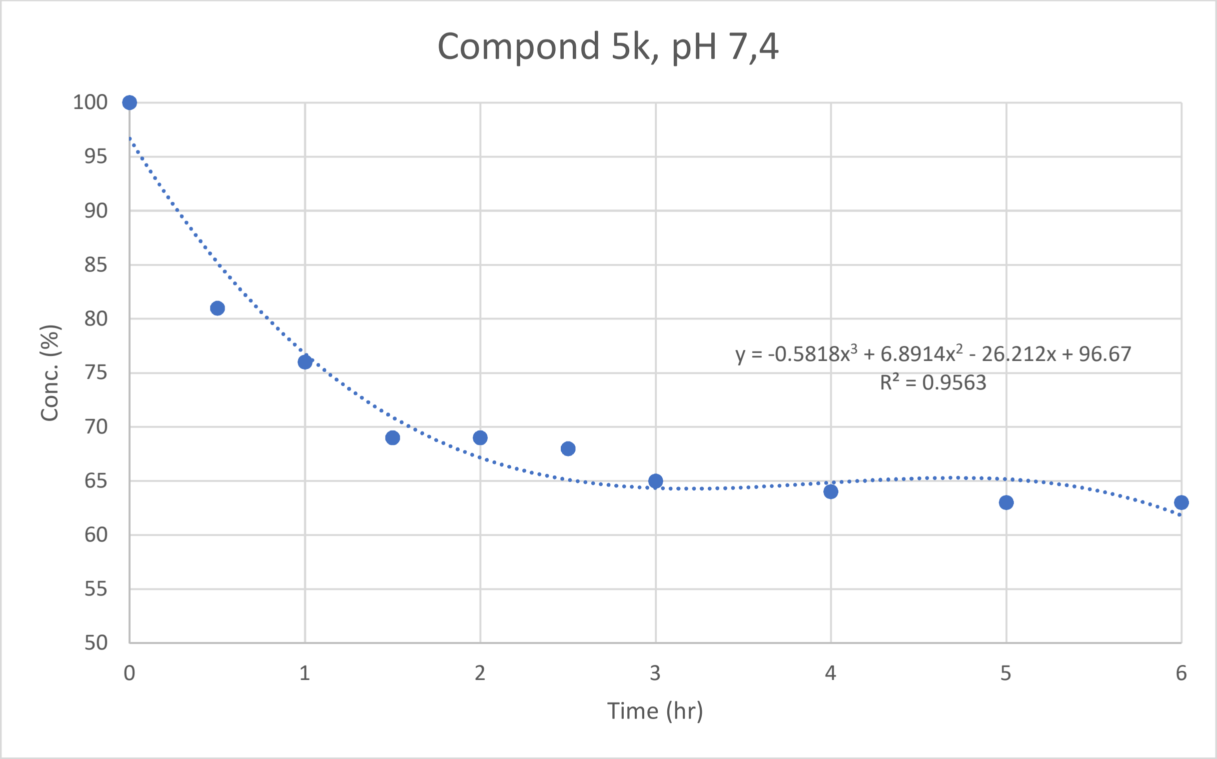

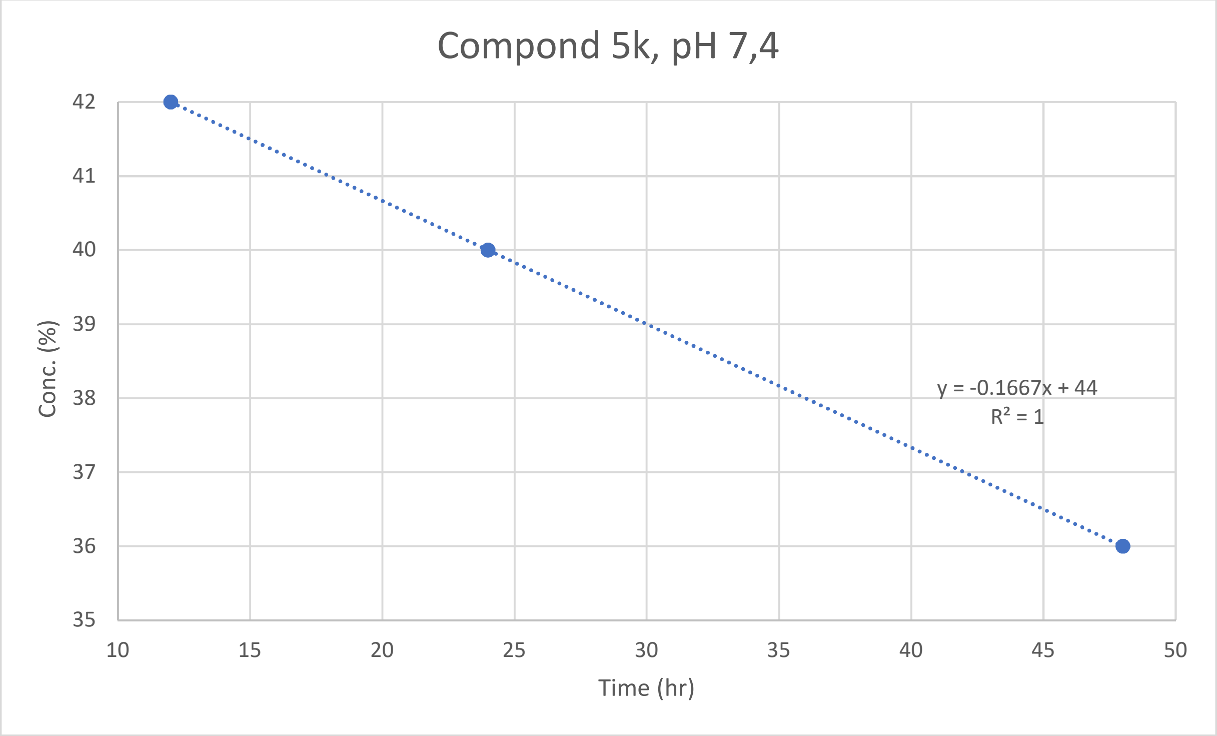

Fig. 2. Decomposition study of compound **5k** at pH 7,4.

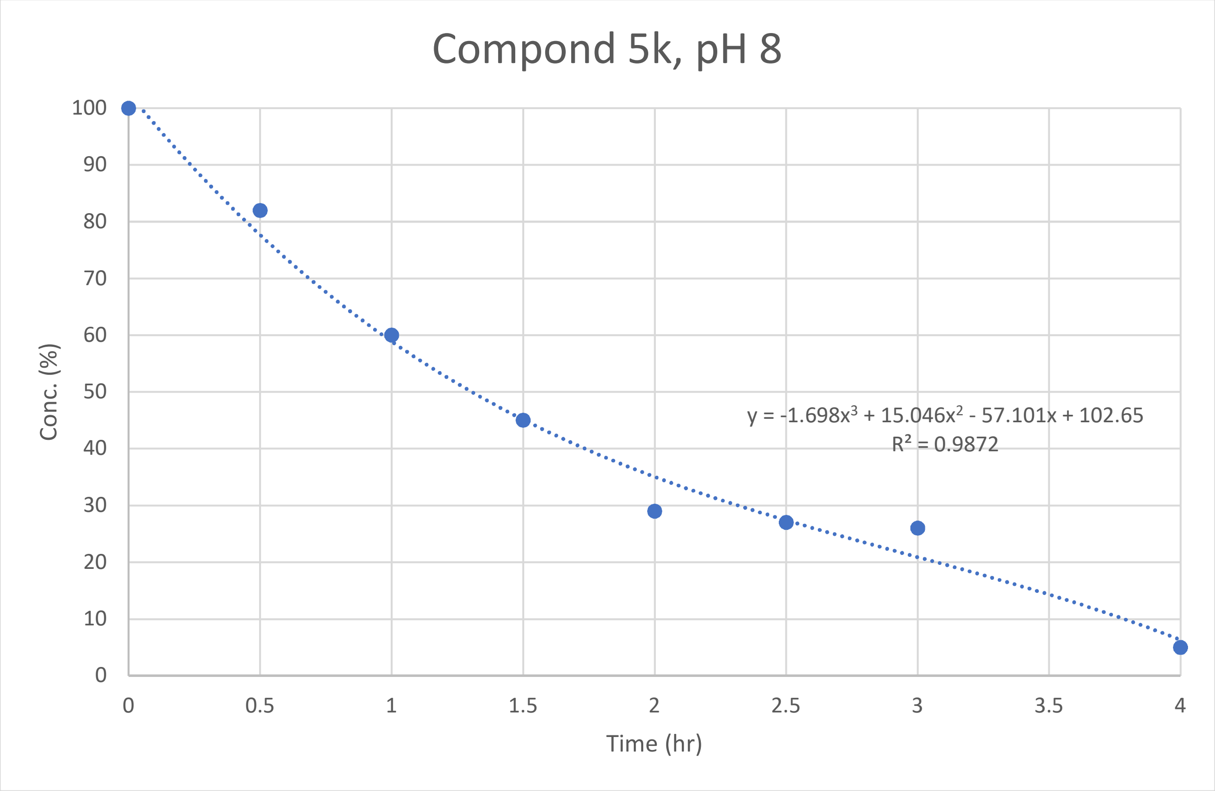

Fig. 3. Decomposition study of compound **5k** at pH 8.

The compound **5k** was chosen for the preliminary study of degradation in three different pH (6, 7.4, 8). Measurement of these degradations was performed by HPLC in reverse phase acetonitrile/water (98:2). The compound was stirred in a tempered buffer (37 °C) of appropriate pH and a sample was taken for HPLC analysis at a specified time. The individual graphs show a clear decomposition of the studied substance as a function of time. In all cases, it is always a pseudo-first order, where one component was in excess and its concentration does not change in time.

Figure 1 represents decomposition study of compound **5k** at pH **6**. The graph shows a significant decomposition in the first hour, when 75% of the substance is rapidly decomposed. However after 6 hrs remains only 13% of starting material in a sample.

In second case (**Figure 1**) If the 5k substance was stirred in a pH 7.4 buffer, its decomposition took twice as long as in the case of pH 6 as it can be seen from the graph. Then there is a gradual decomposition, when only 36% of the starting material remains in 48 hours.

A more interesting situation occurs when the compound 5k is stirred in a buffer of pH 8. Very rapid decomposition occurs as early as 4 hours, when only 5% of the starting material is present in the mixture.

As already mentioned, this is a preliminary study, which was repeated only twice. This is statistically low and it will require further follow-up experiments.

Procedure:

The substance (5k) was dissolved in a buffer of desired pH in Erlenmayer flask and it was placed to tempered water bath (37 °C). At zero time, a sample is taken immediately from solution (1 ml) and analyzed by HPLC (column C18 for reverse phase, mobil phase AcCN/Water 98/2, column temp. 40 °C, flow rate 1 m l/min, injection 10 μl, detection at 214 and 254 nm). Another samples which were taken from solution were at times: 0,5h, 1h, 1,5h, 2h, 2,5h, 3h, 4h, 5h, 6h, 12h, 24h, 48h, 72h and 96h.
